# Supplementary material for: Wood-Inhabiting Nematode, Bursaphelenchus ussuriensis sp. n. (Nematoda: Aphelenchoididae) from David Elm, with Molecular Phylogeny of the Genus Based on Partial Mitochondrial Genomes
Source: Plants (Basel). 2024 Dec 31;14(1):93. doi: 10.3390/plants14010093 (PMC11722724; doi:10.3390/plants14010093)
Supplement: Supplementary file 1 [file plants-14-00093-s001.zip › Bursaphelenchus ussuriensis Suppl. Materials_Table S1.pdf]

Supplementary materials

Table S1. List of *Bursaphelenchus* species used for the mitochondrial genome study.

Species	Location	Sample code	GenBank accession number											
			cox1	*cox2*	*cox3*	*atp6*	*cytb*	*nad1*	*nad2*	*nad3*	*nad4*	*nad4L*	*nad5*	*nad6*
B. cocophilus	Mexico, state of Tabasco	CD3531	PQ528010	PQ474993	PQ474999	PQ474987	PQ475005	PQ475011	PQ475017	PQ475023	PQ590178	PQ590184	PQ590190	PQ590196
B. cocophilus	Mexico, state of Guerrero	CD3548	PQ528011	PQ474994	PQ475000	PQ474988	PQ475006	PQ475012	PQ475018	PQ475024	PQ590179	PQ590185	PQ590191	PQ590197
B. fraudulentus	Russia, Novosibirsk	CD4140	PQ528013	PQ474996	PQ475002	PQ474990	PQ475008	PQ475014	PQ475020	PQ475026	PQ590181	PQ590187	PQ590193	PQ590199
B. michalskii	Russia, Republic of Dagestan	CD3643	PQ528012	PQ474995	PQ475001	PQ474989	PQ475007	PQ475013	PQ475019	PQ475025	PQ590180	PQ590186	PQ590192	PQ590198
B. ussurensis sp. n.	Russia, Primorsky Krai	CD4139	PQ528008	PQ474991	PQ474997	PQ474985	PQ475003	PQ475009	PQ475015	PQ475021	PQ590176	PQ590182	PQ590188	PQ590194
B. willibaldi	Russia, Nizhny Novgorod	CD3664	PQ528009	PQ474992	PQ474998	PQ474986	PQ475004	PQ475010	PQ475016	PQ475022	PQ590177	PQ590183	PQ590189	PQ590195
