# Supplementary material for: Wood-Inhabiting Nematode, Bursaphelenchus ussuriensis sp. n. (Nematoda: Aphelenchoididae) from David Elm, with Molecular Phylogeny of the Genus Based on Partial Mitochondrial Genomes
Source: Plants (Basel). 2024 Dec 31;14(1):93. doi: 10.3390/plants14010093 (PMC11722724; doi:10.3390/plants14010093)
Supplement: Supplementary file 1 [file plants-14-00093-s001.zip › Bursaphelenchus ussuriensis Suppl. Materials_Table S2.pdf]

Supplementary materials

Table S2. Nucleotide alignment of mtDNA sequences for the studied *Bursaphelenchus* species

```
>B_xylophilus_NC_023208
ATTTTAAAGTTTAAATTTCAAGAAAGTTATAAATATTGATTTGAGAGTTCAAATCATAAA
GATATTGGAATATTATATTTCTTATTTGGGTTTTGGTCAGGAATATTAGGTACTAGTTTA
TCAATGGTGATTCGTTTTGAGTTGGCTAAACCTGGTTATTTTTTAAGAAATGGGCAGTTA
TATAATAGTATAATTACTGCGCATGCTTTATTAATGATTTTTTTTATAGTTATACCTTCT
ATAGTTGGAGGTTTTGGTAATTGAATAGTACCTTTGATATTAGGTGCTCCTGATATAAGG
TTTCCGCGTTTAAATAATTTAAGATTTTGGTTATTACCTACTTCTTTATTATTACTCTTA
GATGCTTGCTTTGTTGATACTGGTGCAGGAACTAGATGAACTGTTTATCCTCCATTAAGA
ACTTTAGGGCATCCTGGTGGAAGAGTTGATTTAGCTATCTTTAGTTTACATTGTGCAGGG
GCAAGATCAATTTTAGGAGGAATTAATTTTATATGTACAACTAAAAATTTACGTAGAAGT
TCAATTTCTTTAGAGCATATAAGATTATTTGTATGAACTATTTTTGTTACAGTTTTTTTG
TTAGTTCTCTCTTTACCTGTATTGGCAGGAGCTATTACAATATTATTAACTGATCGTAAT
ATTAATACTTCTTTTTTTGACCCAAGAATAGGAGGGAACCCTTTAATTTATCAACATTTA
TTTTGATTTTTTGGTCATCCTGAAGTTTATATTTTGATTTTACCTGCATTCGGGATTGTA
AGTCAAAGAACTTTATATTTAACTGGTAAAAAAGAAGTTTTTGGTTCTTTAGGAATAGTT
TATGCTATTTTAAGAATTGGTTTAATTGGTTGTGTAGTATGAGCTCATCATATATACACA
GTAGGAATGGATTTAGACTCTCGAGCTTATTTTACTGCTGCTACTATAGTAATTGCTGTA
CCAACAGGAGTAAAAGTTTTTAGTTGGTTAGCAACTTTATTTGGTTCAGTAATAGTATTT
CAACCTTTATTATTATGGGTGTTAGGTTTTATTTTTTTATTTACTATTGGTGGTTTAACT
GGAGTTGTGTTATCTAATTCAAGACTAGACATTATTTTACATGATACTTATTATGTTGTA
AGGCATTTTCATTATGTTTTAAGGTTAGGAGCGGTATTTGGAATTTTTACTGGAGTTGCT
TTGTGATGAACTTTAATTATAGGTTTTGTGTATGATAAAATATTATTTGTGACTGTATTT
ATTTTAATGTTTATTGGAGTAAATTTAACATTTTTTCCATTACATTTTGCTGGTTTACAA
GGGTATCCTCGTAAATATTTAGATTATCCTGATATTTATTCAGTTTGAAATATTATTTCT
TCTTTTGGGAGAATACTAAGAGTTTTTTCTCTTTTATTATTTATTTATTTGATGTTAGAT
TCTTTTAATAGATTTCGTTTATTTATTACTGAGTATAGGCTAAATTATAGTCCTGAAAAT
ATATTATCTGGTTATATTTTTAATCATAGTTATCAATCTGAAATATTTTTTATTGTGAAA
TAAATTTTTAATTATTTTCAAGGTTATAATTTAAATTTTTCTAGTAGTATTTTTTCTTTT
TATATAGATTGATTTCATAGATTTAATTGTAGTTTATTATTAGGAGTATTAATTTTTGTA
AGTTTACTTTTTTTGTTTTTAATTTTTAATAATATTTTTTTTAAAAGTAGGAAAATTGAG
TATCAATTTGGAGAGTTACTTTGTAGTTTGTTTCCAACTTTAATTTTGTTATTTCAAATA
GTTCCTTCTTTAAGATTATTATATTATTATGGATTGATAAATATTGATTCTAATTTTACA
GTTAAGGTTATTGGTCATCAATGATATTGAAGTTATGACTATAGAGATTTTGAGAATTTA
GAATTTGATTCTTATATAAAAAGTGTTGATTCTCTTTTAGTAAGAGAATTTCGTTTGTTA
GATGTTGATAATCGTTGTGTAGTGCCAGTTGAAGTTAATATTCGATTTTGTATTACTTCT
GCTGATGTTATTCAGGCTTGAACTTTATCAAGGTTATCATTTAAATTAGATGCTATAAGA
GGTATTTTAAGTATTTTAAATTATAATTTTCCTTTAATTGGAGTTTTTTATGGTCAATGT
TCAGAAATTTGTGGAGCTAATCATAGGTTTATACCTATTGTTGTTGAAGTTACTTTATTT
GAATTATTTAAATCATGGTGTATACTAGTTTAG------------ATTTTTCATAATTTT
CATATTTTAACTTTATCTAGTTACCCTTATCAAGTGTTTTTATGTATTTTAGGTTTAACT
AGTTCTTTAGTTGTATTTTTAAAGTTTGGTTTATTATTTTCTCTTTTATTCAGATTTTTA
ATTTTATTTTATGTATCTTTTTTATGGGCTAAAGATATTAGTTTTGAGGGGTTATCAGGT
AGTCATAATTTTTATGTTATAGATGGTTTTAAATTTGGTGTAATTCTTTTTATTTTTAGT
GAGTTTATATTTTTTTTTAGAATTTTTTGAACTTTTTTTGATGCGTCATTAGTTCCTACT
CATGATTTAGGAGAAATGTGAAGTCCTTATGGTTTAGTTTTAGTTAATCCTTTTGGGGTT
CCTCTTTTAAATACTATTATTTTATTAAGAAGTGGGGTAACGGTTACTTGAGCTCATTAT
AATTTGTTAAGAAGTAAAGATAGCTTAGTTGGTCTTGTGGTAACTATTATTTTAGCTTTC
TACTTTATATTAATTCAGTTAATAGAGTATAAAGAAGCTAGATTTTCAATTTCTGATGGA
GTTTACGGTAGAATTTTTTATTTATCTACAGGATTTCATGGTTTTCATGTTTTATGTGGT
GGATTATTTTTAGCTTTTAATCTTTATCGTTTGATTTTAAATCATTTTAATTTTAATCAT
CATTTAGGTTTAGAATTTGCTATTATTTATTGACATTTTGTTGATGTGATTTGGTTATTC
TTATTTGTGTTTGTTTATTGATGATCTTTTTA-ATTAATAATGTAACTTTTTTAGATATT
ATTTTATTTATTTTTATTTTACAATTTATTTATTTTTTAGATATTAATTTATTATTTTGT
TTTGTGAAGAAGTTTCTTTTAAGGTTAAACGAAGTTTTTAGGTATTCTAAAAGTTTATTT
TTAAGTAGATTTATTTCTTGTTTTATTTTTTTAGTTTTACTAACTTGTTGTTTCGGAGGC
TACTTTAGGTATTCGTTTTGTCCTTGTGGAATACTTGAATTTACTTTAAGTTTAGCTTTG
ATTTCTTGGTTAAGAACTTTTTTGAGTTTTATTTCAAGAGAGAAAGTCTCAGTTTATTTT
AGTAAAGGAGGTGACACTTTTTTAAAAACTTTAAGAATATTAGTAGTAGAAATTGTAAGA
GAATTTTCTCGTCCTATTGCTTTGACTGTACGTTTAACTGTTAATATTATAGTTGGTCAT
ATAATCAGTATGTCTTTGTATATATCCGTAGAA---AGATTAGGTTACTCATATTTTTTT
TTAATTATTTTTGCTATTTTAATAGAATGTTTTGTTTTTTTTATTCAAAGTTATATTTTT
TCACGTTTAATTTTTTTATATTTAAATGAGT--ATAAAAAATTTATTTGTATTTGTAAAA
GGTTTATTGGTTAATTTACCCACTAGTAAAAGTTTAAGCCTTTATTGAAATTTTGGAAGG
CAGTTAGCTTTAGTATTATTTTTTCAAATTTTGAGAGGAACTTTTTTAGTTTTTTTACTAT
TCTAATGAAAGTGTAATGGCTTTTAGAAGAGTTCAATATATTATAATAGAGGTTAACTTT
GGATGGTTATTTCGTATTTTTCATTTTAATGGAGCTAGATTATTTTTTGTTTTTTTGTAT
```

TTACATTTTTTTAAAGCATTATTTTTTACAAGTTATCGTTTGTTTAAAGTTTGAATAAGA
GGACTATTAATTTTTTTATGTATTATAATAGAAGCTTTTATAGGTTATGTATTAGTTTGA
GCTCAAATAAGATTTTGAGCTTCTGTAGTAATTACAAGATTGTTAAGAGTTATTCCTATT
TGAGGTCCGTTAATTGTTTCTTGAATTTGAAGGGGTTTTAGTGTTTCAGGTGCTACTTTA
AAATTTTTTTTTGTTTTACATTTCCTTTTACCTTGGTTATTTTTAGTTTTAGTATTAGTT
CATTTAATTTTGTTGCATGATACAGGGAGAACTTCTAAAATTCTTACGTTGAGAGATTTT
GAGAAAATTAATTTTTATTATTTTTTTTGATGAAAAGATGGTTATAACGTATTAGTTTGA
ATCTTATTTTTTGTGTTTAGCTTATTTTTACCTTTTTTGTTGGGTGATCCTGAGATATTT
ATTGAAGCGGATCCTATGTTAAGTCCTGTTCATATTGTACCAGAATGATATTTTTTATTT
GCTTACGCTATTTTACGGGCTATCCCTAATAAAATTTTAGGTGTTTGTTTTTTATTATTT
AGAATTTTAATTTTTTTAATTTTTGTTATTAAAGTAAATTTTTTTAGTATTTTAAAAAAT
TTTAATTTTTTTTTGGTAAATATTTTCTTGTATGTAAGAATTTTTTTAAGATGATTAGGA
CAATGTGTTGTAGAAGTTCCATTTTTATTTTTAAGAGGGTTGTTCTCTTTTTTATATTTT
TTTTTAATTTCTTTAATTTTCTTAAATTATAATTTAAGAAATTATTTATTTAAAT-----
------------------------ATTCTAATATCTTTTTTAATAAGTTTGGTATTTGTT
TTGTTTATTATTTTAAGTATTGCTTTTATTACTTTGTATGAACGTCATTTGTTAGGTTTA
AGTCAAAATCGTTTAGGACCAAATAAAGTTTTTTTTTTAGGAATTTTGCAAGCAGGTTTA
GATGGTGTTAAATTAATATCTAAAGAGCAAGTATTACCAAAAAATTCTTCGGATATTTAT
TTTTTGTTTATTCCTGGTGTGTCTTTTTTTTTTATATTTTTAGAATGAAGAACATTACCT
TTTTTATTTAATTTTATTAATTTTCAGTTCTCTTTTTTATTTTTATTATGTCTAGTTGGG
GTTACAGTTTATTTTGTAATTATTAGTGGTTTAATAAGTAATTCTAAATATTCTTTTTTA
GGGGCTATTCGTTCTAGTAGACAAAGTGTCTCTTTTGAGATTGCTTTTTCTTTATATTTA
TTAAGTTTTATATTATTTTTAAATTCTTTTGATTTTGTTCCAATTTTTAATTTTTTATTA
TTTTATATTTTTTATCCTTTTTTATTAATAGTTTTAGCAGAATTAGCACGTGCTCCTTTT
GATTTTTCAGAAGGAGAAAGAGAATTAGTAAGAGGTTTTAATACAGAGCATTCTAGAATT
AGTTTTGTTTTTTTATTTTTAGGCGAATACGGAGTTTTAATTTTTTTTAGAGTAGTAGCG
AGAGTTTTTTTTTTTTTATTTTAATTTTTTGTTTGTTTTTTTTTATTTTACTTTATTGTTA
TTGATTCGTAGAGCTTTTCCACGTTTTCGTTATGATATATTAATAGATTTATTTTGAAAA
GTAATTTTACCACTTTCTTTATTATTTTTAATTTTTTATTGAATTATTTTATAT------
---------ATTTTTTTTGTTATTGTATATTTATTATTTTTTTTTCTTGTATTAAATTTT
TATAATTTTGTTGTGTTATGAAGAATTTTTTTATTAATGAGAATTACTTTTTTTTTTTG
AGTAAATTAATACTAAATGAAAGTATTATTATTTTTTTATTTTATTGTTCAAGAAAGTTTA
GGTCTTATATTTTTGTTATTTTTTATATTTGATTTTCAGTGATTAATTTTGATAATTAAA
ATAGGAGTTTCTCCTTTTATTTTTTGAATATTTTATTTGTTAGAGTTAATAAATAGCTTT
ATAATTTTTTGATTTTTGACTTTTCAAAAATTACCTTTTGTGCCTATAGTAAAAGATTT-
--TTTAGTAAATTTTTTTTTATTTTTTTTGTGTTTGGAATTTTATTTATTTATTTTTTTAATT
TTTTTTGTAAAAAAATCTAAATTTTTAATAATATTAAATTCTATTGAATCTTTTAATTGA
ATTTTATGTTTAATTTATTTTAATTATTTTAATTATTTTTATTTATTTTTTTTTTTATTTA
ATAAGATTTTTTTTATTATATTATTATTTCTCTTTTTTAGAT---TATACTTTTAGTTTA
GTTTTAATTTTATTTTTATTGAATTTTCCTTTTTTTTTTAGATTTTTTTTTAAAATTTTTT
AGGTTATCGTTTTTTAGAATAATAAATTTTTTTTCATATTTAATTTTAGTTTTAATAATT
TTTAATTTAATTTCTTTAGTATATTTATTTTTTATTTTTTTTTTTAAATTTTTTTTAATTTA
AAAAAAAATCAATTTAATTATTTTTTTTTTTTTTTTTTTTTTTTATTTGTTAATTTTGTTTTAA
---------------ATTATTTTTTTTTTTTGTTTTTTTTGGTTGTTTATTTTTATTTTTA
TTTTATGTTTTAAATATTATATTGTCTTTTAAATTTAATTTTTTTTAATAAGGTAAGTTCT
TTTGAGAGGGGGTTTGTAAATATTGGAAAATTATTTAAATCATTTAGAATTCATTTTTTT
TTAATTATAATTTTGTTTGTTATTTTTGATTTAGAAATTATTTTTATTTTAATATTTTTA
GTTAATGATTATGAAGGAGTTTATATTTTTTTTTATTATTTTTTTTTTTTTATATTATTTAGA
TTGTATTTAGAATGATATTTTATAAAATTAGTTTGAGTATTTTAA------ATAATTTTT
ATTTTTATAATATTAAATTTTATTTTTGATTTAAAATTTTTATTTTTTTTTTTTTTTATTT
TTTTTTTTTAAATTTAATTTTTTTTTTTTCTTTTTTAGGTCTATTTTTTTTTTTTTGAAAGT
ATTTTTATGTTTGTATTAGTTTTTATAAGACTTTTAATTTTAAGTTTAATTTTTTTTGAGA
GAAGTAATTTTTTAATTTGAAATTATTAAGGGGGTTTTTAGTAATTGTAAGTTTATTTTTT
TTTCTTAGAAGAAATTTTTTTTTTTTATTTATTTATTTTTTTGAGTTTTCTTTGTTTCCAATT
TTATTAATGTTATTGGGGTATGGTTATCAAATTGAGAAAATTAATGCTGCTTATTTTTTA
TTTATTTTTACTATATTTTTTTCTATACCTTTTTTTTTTTTTTTTGTTCAATAATTGATTT
TACTTTGATAACTTTTTTTTTGATTTTTTTTATAGATGAGAATTATTTTTGTTTTTATCT
TTAATGTTTATATTAAAATTCCCAGTTTATTTTTTACATTTTTGGTTACCTAAAGCTCAT
GTAGAAGCTCCTACAACAGCTAGAATACTTTTAGCAGGGTTATTACTTAAATTAGGTACT
GGTGGGTTTATTCGTTTATTATTTTTTTTTAAAATATTTTTTTTTAGGTAGATATTTTTTA
TTAGCTTTAATTGGAATAATTTTAAGAAATTTTATGTGTATTATTCAAAGAGATCTTAAA
GCATTAGCTGCTTTTTCTTCTATTAATCATATAAGATTAGTTTTATTAAGTTTATTGTTA
TTAAGATTTTCTAGAATTTTAAATAGGGTTGTAATTATGTTTTCTCATGGGTTAATTTCT
ACTCTAATATTTTATTTTATTGGTGAATTTTTTCATTTTACTGGAGTTCGGTTAGTTTAC
TATTATAATAGAATTTTTAATAGAAGTATTGTATTTTCTATTATAATCTTATTTGTTTGA
CTTTACAATAGAGGTATTCCATTTTCTTTAACATTTTTTTCAGAATTTATAATTTTTTTA
GTTGGTTTTAATAGAAGTTATTTTTTTTTTTTTTTTTATTTTTTTTTATATTTTTTTTTAACT
TTTTATTATTGTTTGTTTTTTATTTCTTTAAATTTTTTAGGAAAAAACTTTATTTTAATA
AATTTTAATAATAATTATGGTTTATCATTATTTATAATTGTTATAATATATAATATTTTT
TGGTTAACTATATTCTTTTAA---------------------------------------
--ATTAAT

TTGATATTTTTTTTAGTATTTTTGGGGTTTATATTTATATTTATTATTTTTTTTTTTTTT
TATTTTTATTTTATAGTTAATTTATTAAATTGAAGTTTTTTCATTTTGTTTTTTAATTTA
ACTTTCTTGAAGTTATTATTTTTTTTTTTTTTTGTTATTAGTTGTTATAAGTGTTTTATTT
TTTGTTAATTTTTATTTAGAGGGAGAGGTAAATTTTGTTTATTTTTTAGTAGTTTTATTA
ATTTTTATTTTTAGAATAGTTTTTTTAGCTTTTAGAAATAATTATTTAATTTTAATATTA
AGATGAGATGTTTTAGGTATTTCTAGTTATTTTTTAGTATTGTTTTATAATAATTGAGAT
AGTAGAGTAGGTTCAATAAATGTGGCTTTAACTAATCGTTTAGGTGATTTTTTTATGTTT
TTTTTTTTTCTTTTTTTATATTTTTAAATTTTTTTTTAAATTTTTCTAGGGTGATAGTA
TTTTTAGTATTATTATTTTTATTATTAATAGGTTTTACTAAAAGAGCACAATTTCCTTTT
AGTAGGTGATTACCGAAAGCTATAAGAGCTCCTACTCCTGTAAGAGCTTTAGTACATAGA
AGAACTTTAGTAACAGCTGGTTTAATTTTATTTTTAAATTTTAAATTTTATTTATTTTTT
AGTTTATTTTTGAAATTTATTTTTTTTTTTGGCTTATTTACTATATTTTTTGCTAGTATT
ATAGCTTTTTTGGAAGAAGATTTAAAAAAAGTAGTTGCTTTAAGTACTTTATCTCAAATA
GGTTTTAGTATATTAATTTTAGGGTTAAGTCTTTATTTTTTTAGTTTATTACATTTAGTA
AGACATGCTTTATTTAAAAGTTTTTTGTTTATTCAAGTGGGTATTTTAATTTATTTTTCA
TTTGGTTCTCAGGATAGACGGGGGTACAGTGGTTTACAGTTTTTTTTACTTTTAGTAAAA
TGAAATTTTATTTTAACTTTATTTTGTTTATGTGGTTTATTTTTTAGAAGAGGTTTAATA
AGTAAAGATATTGTTTTAGAGTTTTTTTTTTTTTAATAATTTTGTTTTTGTTTTAGTAATT
ATATTTTTTTTTTCTGTTTTTTTTACTTTTTTTTTATTCTTTTCGATTATTTAAAGGTTTG
AAGAAAATTTTTTTAAATTTTTATTTTTTAGTTTTAAATTTTAGATTTTGTTATATAGGT
ATTTTGCTTTTTTTAATATCTTTAGTTGGAATTACTTTTTTAGTGAATAATATTTTAGTA
TTTCCTAGTTTAATATTAATAGAAGATTTTTTTTCTCCTTTATTTTTTATTGTTATTTTT
TTTATTGTGTATTTTTTTGTTTAATTTAAAGTTAATTTATTTTAAGTATAGTTTTATAGTA
GATTATTATGCAAAAATATTTTCTTTTTTTTTATTTAATTTAAAATTTGTAGATTTATTT
TTTAATAGTAATTTTTTTTATTTAAATTTTAATTTTAAAAAGGTATTTTTATTTTTCTTA
GATTTAAATTATAACTTTTATTTTTTTTTTTATTTTTTTTATATTATTTTTTTATTATATTT
TAA---
------ATTATTTTTTATTATTTTTTTTTTTTTAATAATTTTAATTTGTTATTTTAGTTTA
ATAAGATTTGACCCTTTTAAGAGTTGTTTATTAATGATCTTAAGTTTAATATTAATATCT
ATTATTTTAAGATTTAGAAAATTTATTTGAGTTAGATACTTTGTATGTTTATTATTTTTG
AGAGGAATTTTTGTTATTTTGGTATATTTTTCTAGGTTAAGAAGTTTTTTTTTTTTTTT
AGAAATTATTTTTTAGTAAGATTTTTATTAATTATTAATTTATTTTTTATTATTAGTTTT
AACTTTTTAAATTATAATTTAAGTTATTTTTTTTTATAATATTTATTTATTATTTTTTATT
TTTTTTATTATTGTTTTATTTTATTTTTTAGTATTTTTAAGAATATTTTTAAGTTTTAAT
GGAGCTTTACGTAAATTTTAA------------------ATATTTTTTTTTATTTTTAGT
TTATTTTTTTTTATTATAAAATTTTTTCGTTTTATTTTTATTTTATTAAGTTTTGAGTTT
TTTGTTCTAGGTTTATTTTATTATTTTTCATTTAATTTTAGAAGTTTTTATTTTTTTTTAT
TTTTTATTATTTAGGGTAGTTTCAAGAGTTTTAGGAATTTCTTTAATAATTTATTTTATT
AAGAGTTATGGTAAAGAAAAGGTTTATTTTTAA
>B_mucronatus_kolymensis_sample1

----------TATTATACTTTTTATTTGGTTTTTGATCTGGTATATTAGGTACTAGTTTA
TCTATAGTAATTCGTTTTGAGTTAGCTAAACCAGGATATTTTTTAAGTAATGGACAATTA
TATAATAGTATAATTACTGCTCATGCTTTGTTAATGATTTTTTTTTATAGTAATACCTTCT
ATAGTTGGTGGTTTTGGAAACTGAATAGTTCCTTTAATATTAGGAGCTCCTGATATAAGA
TTTCCTCGTTTAAATAATTTAAGATTTTGGTTATTACCTACTTCATTATTATTACTTTTG
GATGCATGTTTTGTTGATACTGGAGCTGGAACTAGGTGAACTGTGTATCCTCCTTTAAGA
ACTTTAGGTCATCCAGGTGGTAGAGTAGATTTAGCTATTTTTAGATTACATTGTGCAGGA
GCTAGATCAATTTTAGGTGGAATTAATTTTATATGTACTACTAAAAATTTACGTAGTAGA
TCTATTTCTTTAGAACATATAAGATTATTTGTATGAACTATCTTTGTTACAGTATTTTTA
TTAGTTCTTTCTTTACCTGTATTAGCTGGAGCTATTACTATATTATTAACTGATCGAAAT
ATTAATACTTCTTTTTTTGATCCTAGAATAGGAGGAAACCCTTTAATTTATCAACATTTA
TTTTGATTTTTTGGTCATCCTGAAGTGTATATTTTAATTTTACCAGCTTTTGGTATTGTA
AGTCAAAGTACTTTATATTTAACTGGTAAAAAAGAGGTCTTTGGTTCTTTAGGAATAGTA
TACGCTATCTTAAGAATTGGTTTAATTGGTTGTGTAGTTTGAGCTCATCATATATACACA
GTTGGTATAGATCTAGATTCACGAGCTTATTTTACTGCTGCTACTATAGTAATTGCTGTT
CCTACTGGGGTAAAAGTTTTTAGTTGGTTAGCTACTTTATTTGGTTCTGTAATAGTATTT
CAACCTTTATTATTATGAGTTTTAGGTTTTATTTTTTTTATTTACTATTGGTGGTTTAACT
GGAGTTATTTTATCAAATTCTAGTTTAGATATTATTTTACATGATACTTATTATGTAGTA
AGTCATTTTCATTATGTTTTAAGTTTAGGAGCTGTGTTTGGTATTTTTACTGGAGTTGCT
TTGTGATGAACTTTGATTATAGGTTTTGTATATGATAAATTGTTATTTATTTCAGTTTTT
ATTTTAATATTTATTGGAGTAAATTTAACTTTTTTTCCTTTACATTTTGCTGGTTTACAA
GGGTATCCTCGTAAATATTTAGATTATCCTGATGTATATTCTGTTTGAAATATTATTTCT
TCTTTTGGAAGAATATTAAGAGTTTTTTCTTTGTTATTATTTATTTATTTAATATTAGAT
TCTTTTAACAATTTTCGTTTATTTATTACTGATTATACTTTAAATTATAGTCCAGAAAAT
ATATTGTCTGGTTATATTTTTAATCATAGATATCAATCTGATATATTCTTTGTTGTTAAA
TAA---
----TAGATTGATTTCATAGTTTTAATTGTAGTTTATTATTAGGAGTTTTAATTTTTGTA
AGATTACTTTTTTTTTTTCTTATTTTTAATACAGTTTTTTTTAAAAGAAGTAAAATTGAA
TATCAGTTTGGTGAATTATTATGTAGTTTATTTCCAACTTTAATTTTATTATTTCAAATA
ATTCCTTCTTTAAGTTTATTATATTATTATGGTTTAATAAATATTGATTCTAATTTTACT

GTAAAAGTTATTGGGCATCAATGATATTGAAGTTATGATTACAGAGATTTTGAAAATTTA
GAATTTGATTCTTATATAAAAAGAGTAGATTCTCTTTTATTAAGAGAATCTCGTTTATTA
GATGTTGATAATCGATGTGTAGTTCCAGTGGAGGTTAATATTCGTTTTTGTATTACTTCT
GCTGATGTTATTCATGCTTGAACTTTGTCCAGTTTATCTTTTAAATTAGATGCAATAAGT
GGTATTTTAAGAATTTTAAATTATAATTTTCCTTTAGTTGGTGTTTACTATGGGCAATGT
TCAGAAATTTGTGGAGCAAATCATAGTTTTATACCTATTGTGGTAGAAGTAACTTTATTT
GAATTATTTAAATCATGATGTATATTAGTATAG-------------TATTTCATAATTTT
CATATTTTAACTTTATCTAGATACCCTTATCAAATTTTTTTATGTTCTTTAGGTTTGACT
AGTTCTTTAGTAGTTTTTTTAAAATTTGGTTTACTTTTACCTTTGATTATAAGATTTTTA
GTTTTATTCTATATTTCTTTTTTATGAGCTAAAGATATTAGTTTTGAAGGTTTGTCTGGT
AGACATAATTTTTTTGTTATAGATGGTTTTAAATTTGGTGTAATTTTATTTATTTTTAGA
GAGTTTATATTTTTTTTTAGAATTTTTTGAACATTTTTTGATGCTTCATTAGTGCCTACA
CATGATTTGGGTGAAATGTGAAGTCCTTATGGTTTAGTTTTAGTAAATCCTTTTGGAGTA
CCTTTATTAAACACTATTATTTTATTAAGAAGAGGTGTTACAGTTACTTGGGCTCATTAT
AATTTATTAAGAAGAAAAGATAGTTTAGTAAGTCTTTTAATTACTATTATTTTAGCTTTT
TATTTTATGTTAATTCAATTGATAGAATATAAAGAGGCTAGATTTTCTATTTCTGATGGT
GTTTATGGGAGGATTTTTTATTTATCAACTGGTTTTCATGGTTTTCATGTTTTATGTGGT
GGTCTTTTTTTAGCTTTTAATTTGTATCGTCTAATTTTAAATCATTTTAATTTTAATCAT
CATTTAGGTTTGGAATTTGCTATTATTTATTGACATTTTGTTGATGTTGTATGACTATTT
TTATTTGTTTTTGTTTATTGATGATCTTTTTA----------------------------

-------------------------------TATTAGAGTTTACTTTAAGTTTAGCTTTA
ATTTCTTGGTTAAGAACTTTTTTAAGTTTTATTTCAAGAGAGAAAATTTCTATTTATTTT
AGAAAAGGAGGAGACACTTTTTTAAAAACTTTAAGAATATTAGTTGTTGAAATTGTAAGA
GAGTTTTCTCGTCCTATTGCTTTGACTGTTCGTTTAACTGTAAATATTATAGTTGGTCAT
ATAATTAGAATATCTTTATTTTTTATTAGTTGAA---AATTTAGGTTATTTGAATTTTTTT
TTTATTATTTTTGCTATTTTAATAGAATGTTTCGTATTTTTTATTCAAAGTTATATTTTT
TCACGTTTAATTTTTTTATATTTAAATGAGTAT-TAAAAGGTTTATTTTTATTTGTTAAA
GGTTTATTAGTAAATTTACCTACAAGAAAAAGTTTGAGTTTATATTGAAATTTTGGAAGT
CAATTAGCTTTAATTTTAGTTTTTCAAATTTTTAGTGGTACATTTTTAGTTTTTTATTAT
TCTGCTGATAGAATAATTGCTTTTAGAAGTGTTCAGTATATTATGACAGAAGTTAATTTT
GGTTGGTTGTTTCGTATTTTTCATTTTAATGGAGCTAGACTTTTTTTTGTTTTTTTGTAT
TTGCATTTTTTTAAAGCTTTGTTTTTTAATAGATATCGTTTATTTAAAGTATGATCTAGA
GGTTTAGTAATTTTTTTGTGTATTATAATAGAAGCTTTTATAGGTTATGTTTTGGTTTGA
GCTCAAATAAGATTTTGAGCTTCTGTGGTTATTACCAGTTTGTTAAGTGTAATTCCAATT
TGGGGTCCTATAATTGTTTCTTGAATTTGGAGAGGGTTTAGAGTTTCTGGAGCTACTTTA
AAATTTTTTTTTGTTTTACATTTTATTTTGCCTTGATTATTTTTAGTTTTAGTTTTAGTA
CATTTAATTTTATTGCATGAAACTGGAAGAACTTCTAAATTACTTACTTTAAGAGATTTT
GAGAAAATTAATTTTTATTATTTTTTTTGATGAAAAGATGGTTTAAATTTATTAATTTGA
TTATTTTTTTTTATTTTTAGTTTATTTTTTCCTTTTTCTTTAGGAGACCCAGAAATGTTT
ATTGAGGCAGATCCTATACTAAGTCCTGTACATATTGTACCAGAGTGATATTTTTTATTT
GCATACGCAATTTTACGAGCTATTCCTAATAAAATTTTAGGGGTTTGTTTTTTGTTATTA
AGTATTATAATTTTTTTTTTGTTTATTTTAAAAGTTAATTTTTTTAGAATTTTTAAAAAT
TATAATTTTTTTATTGTAAATTTTTTTATTTTTGTTAGAATTTACCTAAGTTGGTTAGGT
CAATGTGTAGTGGAAACTCCATTTTTATTTTTAAGTGGGTTATTTTCTTTCTTATATTTT
TTTTTTATTATTTTATTAATTATTAATTATAATTTAAGAAAATTTTTATTTAAATTTGTG
TTTATTATAATTTA--
----TAGTTATTTTAAGTATTGCTTTTACTACTTTATATGAGCGTCATTTACTTGGTTTG
AGTCAAAATCGGTTAGGTCCTAATAAAGTTTTTTTTTTAGGTGTTCTTCAAGCTGGTTTG
GATGGAGTAAAGCTTATATCTAAAGAGCAAATTTTACCTAAAAATTCTTCGGATATTTAT
TTTTTATTAATTCCAGGAGTATCTTTTTTTTTTTATATTTTTAGAATGGAGAACTTTACCT
TTTATATTTAATTTTATCAATTTTCAGTTTTCTTTTTTTATTTCTTTTATGTTTGGTGGGA
GTAACTGTTTATTTTATTATTATTAGTGGGTTAATAAGGAATTCTAAATATTCTTTTTTA
GGTGCTATTCGTTCTAGAAGTCAAAGTGTTTCTTTTGAAATTGCTTTTTCTTTATATTTA
TTAAGTTTTATATTGTTTTTAAATTCTTTTGAGTTTATTTCTATTTTTAATTTTTTATTA
TTTTTTATTTTTTATCCATTTTTATTAATAGTCTTAGCAGAATTAGCTCGAGCACCTTTT
GATTTTTCTGAGGGTGAAAGAGAGTTAGTTAGTGGTTTTAATACTGAGCATTCTAGAATT
AGGTTTGTATTTCTTTTTTTAGGTGAGTATGGGGTTTTAATTTTTTTTAGTGTAGTAGCT
AGTATTTTTTTTTTTGATTTTAGTTATTTCTTTGTTTTTTTTTTATTTCACTTTGTTATTA
CTTATTCGTAGAGCTTTTCCTCGTTTTCGATATGATTTTTTGATAGATATGTTTTGGAAA
GTAATTTTACCTTTATCTTTATTTTTTTTTATTTTTTTTATTGAATTATTTTGTAT------
----
-------------------------------------TAAGAATTTCTTTTTTTTTTTTA
AGAAAAATTATGTTTAATGAAAATATTATTATTTTTTATTTTATTATTCAAGAAAGTCTA
GGTCTTGTTTTTTTATTATTTTTTATATTTGATTTTCAATGATTAATTTTGATAATTAAG
ATTGGAGTTTCACCTTTTATTTTTTGGTTATTTTATTTAGTTGAGGTTATAAATAGTTTT
TTGTTATTTTGATTTTTAACTTTTCAAAAATTACCTTTTGTACCTATAATTAAAGATTT-
--TTTAGTTAATTTTTTTTATTTTTTTGTTGTTGGTATTCTATTAATTTATTTTATTATT
TTTTTGTTAAAAAAAATAAATTTTTATTGATATTAAATTCTGTAGAATCTTTTAATTGA

ATTTTGTGTGTAATTTATTTTAATTATTTTAACTACTTTTATTTATTTTTTTTTTTATTTA
ATAAGTTTTTTTTTAATATATTTTTATTTTTTATATTTAGAC---TTAACTTATAGACTT
TTAGTTATTTTATTTTTATTGAATTTCCCTTTTTTGTTTAGTTTTTTTTTAAAGTTTTTT
AGTTTATCTTTTTTTAGATATTTGAATTTTAATTTATATATTATTTTGGTTTTAATATTT
TTGAATTTGGTTTCTTTATTTTATTTATTGTTTATTTTTTTTTTAAATTTTATAAATTTT
AAAAAAAATCAATTAAATTATTTTTTTTTTTTTTTTTTATATTTATTAA------------
----
-------------------------------------TAAATTTAAGTAAAATTAGTTCT
TTTGAAAGTGGATTTTTGAATGTTGGTAAATTATTTAAATCTTTTAGAATTCATTTTTTT
ATAATTTTGATTTTATTTGTAATTTTTGATTTAGAAATTATTTTTATTTTAATATTTTTG
GTTAATGATTTTGAGGGTATTTATATTTTTATTTTAATTTTTTTTTTTTATATTGTTTAGT
CTTTACTTAGAGTGATATTTTATAAAATTAGTTTGAGTTTTTTAA---------------

-------TGTTTGTTTTAGTTTTTATAAGTTTAATTATTTTAAGATTAATTTTTATAAGT
GAAGTAGTATTTAATTTAAAATTTTTAAGAAGAGTTTTAGTAATTGTGAGTTTATTTTTT
TTTTTTAGTACAAATTTTTTTTATATTTATTTATTTTTTGAGTTTTCTCTGTTTCCTATT
TTATTAATATTATTAGGTTATGGTTATCAAATTGAGAAGATTAATGCTGCTTATTTTTTA
TTTATTTTTACTATATTTTTTTCTATACCTTTTTTTTTTTTTTTTTTTTAATATTGACTTT
TCTATTTTTAATTATTTTTTTTTATAATTTTTATAGTTGAGAATTATTTTTTTTTTTGTCT
TTAATATTTATACTAAAATTTCCAGTTTATTTTTTACATTTCTGATTACCTAAAGCTCAT
GTGGAAGCCCCTACTACTGCTAGAATACTTTTAGCGGGATTGTTATTAAAGTTAGGTACA
GGTGGTTTTATTCGTTTATTATTTTCTTTCAAATATTTTTTTTTAGGAGGATATTTTTTA
CTATCTTTAATTGGAATAATTTTAAGAAATTTTATATGTATTTTTCAAAGTGATTTAAAA
GCTTTAGCTGCTTTTTCTTCTATTAATCATATAAGTTTAGTTTTATTAAGTTTATTATTT
TTAAGTTATGTAAGAATTTTTAATAGAGTAGTAATTATATTTTCTCATGGTCTAATCTCA
ACTTTGATATTTTATTTTATTGGTGAATTTTTTCATTTTAGTGGTGTACGTTTAATTTAT
TATTATAATAGAATTTTTAGAAGAAGTCTTGTTTTTTCTATTTTAATTTTATTAGTTTGA
CTTTACAATAGAGGAGTACCTTTTTCTTTAACATTTTTTTCTGAGTTTAATATTTTTTTA
GTTGGTTTTAATAGTAATTATTTTTTTTTTTTTTTTTATTTTTTTTTATATTTTTTTTTAACT
TTTTATTATTGTTTATATTTTATTTCATTAAATTTTTTAGGTAAAAATTTTATTTTGTTA
AATTATAATTTAAATTTGTATTCTGCTTATTTTATTTTATTAAATTCTAATATTTTTTTT
TTTATTTTTTTTTGTTTAG--

----------------------TAAGATTTTTTATTGTTTTTATTATGTTTTTTTTTTTG
TATTTTTATTTTATAATTATTATATTAAATTGAACTTTTTTTATTTTTTTTTTTTTAATTTT
ACTTTTTTAAAATTATTATTTTTTTTTTTTTTTTATTAGTTGTAGTAATAAGAGTTTTATTT
TTTGTTAATTTTTATTTGGAGGGTGAAATTTATTTTGTTTATTTTTTTGTTGATTCTTTTG
ATTTTTATTTTTAGAATGATTTTTTTTAGCTTTTAGTAATAATTATTTGATTCTTATATTA
AGTTGAGATATTTTAGGAATCTCTAGTTATTTTTTAGTTTTATTTTATAATAATTGAGAT
AGAAACATTGGTTCTATGAATGTTTCTTTGACTAATCGTTTAGGTGATTTTTTTTATATTT
TTTTTTTTTTCTTTTTTTTATATTTTTAAATTATTTTTTGAGTTTTTCTAGACTTATATCT
TTAATTGTAATATTTTTTTTTATTGGTTATAGGTTTTACTAAAAGTGCGCAATTTCCTTTT
AGAAGTTGATTACCTAAAGCTATAAGTGCTCCAACTCCTGTTAGGGCCTTAGTACATAGA
AGAACATTAGTAACTGCTGGTTTAATTTTATTTTTAAATTTTAAATTTTATTTGTTTTTT
GGTATTTTTTTAAAAATAATTTTTTTTTTTGGTTTGTTTACTATATTTTTTGCTAGAGTG
ATAGCTTTTTTAGAAGAAGATTTAAAAAAAGTAGTGGCTTTAAGTACTTTATCGCAGATG
GGTTTTAGAATATTAATTTTAGGTTTAAGACTTTATTTTTTTTAGTTTATTACATTTAGTT
AGTCATGCTTTATTTAAAAGATTTTTGTTTATTCAAGTTGGAATTTTAATTTATTTTTCT
TTTGGAGCACAAGATAGTCGGGGTTACAGAGGTTTACAATTTTTTTTAATTTTAGTAAAA
TGAAATTTTATTTTAACGTTATTTTGTTTATGTGGTTTATTTTTTAGAAGAGGTTTATTA
AGAAAAGATATTGTTTTGGAGTTTTTTTTTTTTTAATAATTATATTTTTATTTTAATTGTA
ATATTTTTTTTTTCAATTTTTTTTTACGTTTTTTTATTCTTTTCGTTTGTTTTTAGGTTTT
AAGAAAATTTTTTTAAGTTTTTTTTTTGTTATTTTAAATTTTAGTTTTTGTTACATAGGA
ATTTTTCTTTTTTTTATTTTCTTTAGTTGGAATTTCTTTTTTGGCTAATAATGTTTTGTTA
TTTCCTAGAATTACATTAATAGAAGATTTTTTCTCTCCTTTGTTTTTTTATTATTATATTT
TTTTTTATTTTTTTTCTTTTTAAATTTTAAATTTATTTTGTTTAAATTTAGTTTTATAGTT
GATTATTTTGCTAAGATATTTTCTTTTTTTTTTATTTAATTTTAAATTTATTGATTTATTT
TTTAATAATAATTTTATTTATATAAATTTTATATTAAAATTTTTTAGTTTTAAATTATTA
AGACTAAATTATAATTTTTATTTTTTTTTTTATATTTTTTTATGATTTTTTTTTATTTTTTTTT
TAA---

-TAAGTTTTGACCCATTTAAAAGTTGTTTGCTTATAATTTTAAGATTAATATTAATATCT
CTGATTTTGAGTTTTAGAAAATTTGTCTGGGTAAGTTATTTTGTATGTTTATTGTTTCTA
AGTGGAATTTTTGTTATTTTAATTTATTTTTCTAGATTAAGAAGGTTTTTTTTTTTTTT
AGTAATTACATAATTGTTAGTTTATTATTATTTTTTTAATTTAATTTTTATTTTTTATTTTT
AATTCTTTAAATTATAGTCTTAGATATTTTTTTTATAATATTTATTTGATATTTTTTTTT
TTTTTTATTTTAGTTTTGTTTTATTTTTTAGTTTTTTTAAGATCATTTTTTAAGTTTTAAT
GGAGCTTTACGTAAATATTAA-------------TATTAATTTTTTTTTTTTTATTTTTTAGT
TTATTTTTTTTTTATTTTAAAATTTTATCGTTTTATTTTTATTTTGTTGAGTTTTGAATTT
TTTGTTTTAGGTTTATTTTATTATTTTTCTTTTAATTTTAATAGTTTTTACTTTTTTTAT

TTTTTATTATTTAGAGTAGTCTCTAGAGTTTTAGGTATTTCTTTAATAATTTTTTTTATT
AAAAGTTATGGAAAAGAAAAAGTTTATTTTTAG
>B_mucronatus_mucronatus_NC_021120
ATTTTAAAATTTAAATTTCAAGAAAGATATAAATATTGATTTGAAAGTTCAAATCATAAA
GATATTGGAATATTATATTTTTTATTTGGTTTTTGATCTGGTATATTAGGTACTAGTTTA
TCTATAGTAATTCGTTTTGAGTTAGCTAAACCAGGATATTTTTTAAGTAATGGTCAATTA
TATAATAGAATAATTACAGCTCATGCTTTATTAATAATTTTTTTTATAGTAATACCTTCT
ATAGTTGGTGGTTTTGGTAATTGAATAGTTCCTTTAATATTAGGTGCTCCAGATATAAGT
TTTCCTCGTTTAAATAATTTAAGTTTTTGATTATTACCTACTTCATTATTGTTACTTTTA
GATGCATGTTTTGTTGATACTGGTGCTGGAACTAGTTGAACAGTTTATCCTCCTTTAAGT
ACATTAGGTCATCCAGGTGGAAGTGTTGATTTAGCTATTTTTAGATTACATTGTGCAGGA
GCAAGATCAATTTTAGGTGGAATTAATTTTATATGTACTACTAAAAATTTACGTAGTAGT
TCTATTTCTTTAGAACATATAAGATTATTTGTATGAACTATTTTTGTTACAGTATTTTTA
TTAGTTTTGTCTTTACCTGTTTTAGCTGGAGCTATTACTATATTATTAACTGATCGAAAT
ATTAATACTTCTTTTTTTGACCCTAGAATAGGTGGAAATCCTTTAATTTATCAACATTTA
TTTTGATTTTTTGGTCATCCTGAAGTTTATATTTTAATTTTACCAGCTTTTGGAATTGTA
AGTCAAAGTACTTTGTATTTGACTGGTAAAAAAGAAGTTTTTGGTTCTTTAGGAATAGTT
TATGCTATTTTAAGAATTGGTTTAATTGGTTGTGTAGTATGAGCTCATCATATATATACA
GTTGGTATAGATTTAGATTCACGAGCTTATTTTACTGCTGCAACTATAGTAATTGCTGTT
CCTACTGGTGTAAAAGTTTTTAGATGATTAGCTACTTTATTTGGATCTGTAATAGTATTT
CAACCTTTATTATTATGAGTTTTAGGTTTTATTTTTTTATTTACTATTGGTGGTTTAACT
GGTGTAATTTTATCTAATTCTAGTTTAGATATTATTTTGCATGATACTTATTATGTAGTA
AGTCATTTTCATTATGTTTTAAGTTTAGGTGCTGTATTTGGTATTTTTACTGGTGTAGCT
TTATGATGAACTTTAATTATAGGTTTTGTATATGATAAATTATTATTTATTTCAGTTTTT
ATTTTAATATTTATTGGTGTAAATTTAACTTTTTTTCCCTTACATTTTGCTGGGTTACAA
GGTTATCCTCGAAAATATTTAGATTATCCTGATATTTATTCTGTTTGAAATATTATTTCT
TCTTTTGGAAGAATATTAAGAGTTTTTTCTTTATTATTATTTATTTATTTAATATTAGAT
TCTTTTAATAATTTTCGTTTATTTATTACTGATTATACTTTAAATTATAGTCCAGAA---

---ATTTTTAATTATTTTCCAGGATATAATTTAAATTTTTCTAGTAGAATTTTTTCTTTT
TATATAGATTGATTTCATAGTTTTAATTGTAGATTACTTTTAGGAGTTTTAATTTTTGTT
AGATTACTTTTTTTTTTTCTTATTTTTAATAAAATTTTTTTTAAAAGTAGAAAAAATTGAG
TATCAATTTGGTGAATTATTATGTAGATTATTTCCAACTTTAATTTTATTATTTCAAATA
GTACCTTCTTTAAGGTTATTATATTATTATGGTTTAATAAATATTGATTCTAATTTTACT
ATTAAAGTTATTGGACATCAATGATATTGAAGTTATGATTATAGTGATTTTGAAAATTTA
GAATTTGATTCTTATATAAAAAGAGTAGATTCTCTTTTATTAAGAGAATCTCGTTTATTA
GATGTAGATAATCGATGTGTAGTTCCAACAGAAGTTAATATTCGTTTTTGTATTACTTCT
GCTGATGTTATTCAGGCTTGAACAATCACTAGTTTATCATTTAAATTAGATGCTATAAGA
GGTATTTTAAGTATTTTATCCCAACGCGTGAGCTGCGTTGGAGTTTATTATGGTCAATGT
TCAGAAATTTGTGGTGCTAATCATAGATTTATACCTATTGTAGTAGAAGTTACTTTATTT
GAATTATTTAAATCTTGATGTTTATTAATTTAG------------ATTTTTCATAATTTT
CATATTTTAACTTTATCAAGATATCCTTATCAAGTTTTTTTATGTTCTTTAAGTTTAACT
AGATCTTTAGTAGTATTTTTAAAATTTGGTTTAACATTTTCTTTATTTTTAAGTTTAATT
ATTTTAATTTATGTTTCTTTTTTTATGAGCTAAAGATATTAGTTTTGAAGGTTTATCAGGT
AGTCATAATTTTTTTGTTATAGATGGTTTTAAATTTGGAGTAATTTTATTTATTTTTAGT
GAATTTATTTTTTTTTTTAGAATTTTTTGAACATTTTTTGATGCCTCCTTAGTACCTACT
CAAGATTTAGGGGGAAAATGGAGCCCCTATGGGTTAGTATTAGTAAATCCTTTTGGAGTA
CCTTTACTTAATACTATTATTTTATTAAGAAGTGGTGTTACTGTTACTTGGGCTCATTAT
AATTTATTAAGAAGTAAAGATAGTTTAGTAAGTCTTTTTATTACTATTATTTTAGCTTTT
TATTTTATATTAATTCAATTAATAGAATATAAAGAAGCTAGTTTTTCTATTTCAGATGGT
GTTTATGGTAGAATTTTTTATTTATCAACTGGTTTTCATGGTTTTCATGTTTTATGTGGT
GGACTTCTTTTAACTTTTAATTTATATCGTTTAATATTGAATCATTTTAATTTTAATCAT
CATTTGGGATTAGAATTTGCTATTATTTATTGACATTTTGTAGATGTTGTATGATTATTT
TTATTTGTATTTGTTTATTGATGATCATTTTA-ATAAATAATGTTACTTTTCTAGATATT
TTTTTGTTTGTTTTTATTTTACAATTTATTTATTTTTTTTAATATTAATTTATTATTTTGT
TTTATTAAAAAATTTTTATTTAGTTTAAATGATGTTTTTAGATATTCTAAAAATTTATTT
TTAAGTAGAATTATTTCTTGTTTTATTTTTTTAGTTTTGTTAACTTGTTGTTTTGGTGGA
TATTTTAGATATTCTTTTTGTCCATGTGGAATATTAGAGTTTACTTTAAGTTTAGCTTTA
GTTTCTTGATTAAGAACTTTTTTAAGATTTTTATCAAGAGAAAAAATTTCTATTTATTTT
AGAAAAAGAGGTGATACTTATTTAAAAACTTTAAGAATATTAGTTGTAGAAATTGTAAGA
GAGTTTTCTCGACCTATTGCTTTAACTGTTCGTTTAACAGTTAATATTATAGTTGGTCAT
ATAATTAGAATATCTTTATTTTTATTACCAGAA---AATTTAGGATATTATAATTTTTTT
TTTATTATTTTTGCTATTTTAATAGAATGTTTTGTTTTTTTTTATTCAAAGTTATATTTTT
TCACGTTTAATTTTTTTATATTTAAATGAATAGATAAAAGGTTTATTATTGTTTATTAAA
GGTTTATTAATTAATTTACCAACAAGAAAAAGTTTAAGTCTTTATTGAAATTTTGGTAGT
CAATTAGCTTTAATTTTAATTTTTCAAATTTTTAGTGGTACATTTTTAGTATTTTATTAT
TCTGCTGATAGAATAATTGCTTTTAGAAGTGTTCAATATATTATAACTGAAGTTAATTTT
GGTTGATTATTTCGTATTTTTCATTTTAATGGGGCAAGTTTATTTTTTATTTTTTTGTAT
TTACATTTTTTTAAGGCTTTATTTTTTAATAGATATCGTTTATTTAAAGTATGATCTAGA
GGTTTAGTAATTTTTTTATGTATTATAATAGAAGCTTTTATAGGATATGTTTTAGTATGA
GCTCAAATAAGTTTTTGAGCTTCAGTTGTTATTACTAGTTTATTAAGTGTTATTCCAGTT

TGAGGACCTATAATTGTTTCTTGAATTTGAAGTGGTTTTAGTGTTTCTGGTGCTACTTTA
AAATTTTTTTTTGTTTTGCATTTTATTTTACCTTGATTATTTTTAGTATTAGTTTTATTA
CATTTAATTTTCTTACATGATACTGGAAGAACTTCTAAAATTTTAACTTTAAGAGATTTT
GAAAAAATTAATTTTTATTATTTTTTTTGATGAAAAGATGGATTAAATTTATTAATTTGA
ATATTATTTTTTGTTTTTAGATTATTTTTTCCTTTTTTTTTAGGTGATCCAGAAATATTT
ATTGAAGCAGATCCTAATACTTATCCTGTACATATTGTACCAGAATGATATTTTTTATTT
GCATATGCAATTTTACGAGCTATTCCTAATAAAATTTTAGGGGTTTGTTTTTTATTATTA
AGAATTTTAGTTTTTTTTTTTATTATTGTTAAAGTTAATTTTTTTAGAATTTTTAAAAAT
TTTAATTTTTTTATTGTATTTATTTTTATTTTTACTAGAATTTATTTAAGATGATTAGGT
CAATGTGTAGTAGAAACTCCTTTTTTATTTTTAAGAGGTTTATTTTCTTTTTTATATTTT
TTTTTAATTTTTTTAATTATTTTAAATTATAATTTAAGAAATTTTTTATTTAAAT-----
------------------------ATAATTCTATCTTTAATTTTAAGTTTATTTTTTGTT
TTAATAGTAATTTTAAGAATTGCTTTTATTACTTTGTATGAACGTCATTTATTAGGTTTA
AGACAAAATCGTTTAGGGCCAAATAAAGTTTTTTTTTTAGGGGTTCTTCAAGCTGGTTTA
AATGGAGTTAAACTTATGTCAAAAGAACAAATTTTACCTAAAAATTCTTCTGATATTTAT
TTTTTGTTAATTCCTGGAGTTTCTTTTTTTTTTTATATTTTTAGAATGAAGAACTTTACCT
TTTAAATTTAATTTTATTAATTTTCAATTTTCTTTTTTATTTCTTTTATGCTTAGTAGGA
GTAACAGTTTATTTTATTATTATTAGAGGATTAATAAGTAATTCCAAATATTCTTTTTTA
GGTGCTATTCGCTCTAGAAGTCAAAGTGTTTCTTTTGAAATTGCTTTTTCTTTATATTTG
TTAAGTTTTATATTATTTTTAAATTCTTTCGAATTTACTTCTATTTTTAATTTTTTATTA
TTTTTTATTTTTTATCCTTTTTTGTTAATAGTTTTAGCTGAATTAGCTCGTGCTCCTTTT
GATTTTTCAGAAGGAGAAAGAGAATTAGTAAGTGGTTTTAATACTGAACATTCTAGAATT
AGTTTTGTATTTCTTTTTTTAGGTGAATATGGTGTATTAATTTTTTTTTAGTGTAGTTTCA
AGTATTTTTTTTTTTGATTTTAGTTTTTTTTTTGTTTTTTTATATTTTTCTTTATTATTA
TTAATTCGAAGAGCTTTTCCTCGTTTTCGGTATGATTTTTTAATAAATATATTTTGAAAA
ATTGTTTTACCTTTATCTTTATTTTTTTTATTTTTTTTATTGAATTGTTTTATAT------
---------ATTTTTTTTATTTGTATATACTTATTTTTATTTTTTTTAACTTTAAATTTT
TTTAATTTTGTTATTTTATGAAGTGTATTTTTATTAATAAGAATTTCTTTTTTTTTTTTA
AGTAAAGTTTTATTAAATGAAAGTATTATTATTTTTTTATTTTATTATTCAAGAAAGATTG
GGTCTTGTTTTTTTATTATTTTTTTTTATTTGATTTTCAATGATTAATTTTAATATTAAAA
ATTGGAATTTCACCTTTTATTTTTTGATTATTTTATTTAGTTGAAATTATAAATAGTTTT
TTATTATTTTGATTTTTAACTTTTCAAAAATTACCTTTTATTCCTATAATTAAAGATTT-
--TTTAGTTAATTTTTTTTTATTTTTTTTGTTATTGGTATTTTATTAATTTATTTTATTATT
TTTTTTGTTAAAAAAAAATAAATTTTTATTAATATTAAATTCTGTAGAATCTTTTAACTGA
ATTTTATGTTTAATTTATTTTAATTATTTTAATTATTTTTATTTATTTTTTTTTTTATTTA
TTAAGATTTTTTTTAATATATTTTTATTTTTTTTTATTTAGAC---ACTAATTATAGATTT
ATTATAATTTTATTTTTATTAAATTTTCCTTTTTTATTTAGTTTTTTTTTAAAATTTTTT
AGTTTATCTTTTTTTAGTTTTATAAATTTTAATTTATATATTATTTTAATTTTAATATTT
TTAAATTTGATTTCTTTATTTTATTTATTATTTATTTTTTTTTTTAAATTTTTTTAATTTA
AAAAAAAATCAAATGAATTATTTTTTTTTTTTTTTTTTTTTTTTTTATTTACTAATTTTGTTT
TAA------------ATATTATTTTTTTTTTATTTTTTTTTGGGTTATTGTTTTTATTTTTA
TTTTATTTAATTAATATTATTTTATCTTTTAAATTTATAAGTTTAAATAAAATTAGTTCT
TTTGAAAGTGGATTTTTAAATGTTGGAAAATTATTTAAATCTTTTAGTATTCATTTTTTT
ATGATTTTAATTTTATTTGTAATTTTTGATTTAGAAATTATTTTTATTTTGATATTTTTG
GTTAATGATTTTGAAGGAATTTTTATTTTTATTTTAATTTTTTTTTTTATATTATTTAGA
CTTTATTTAGAATGGTATTTTATAAAATTAGTTTGAATTTTTTAA------ATTATTTTT
ATTTTTGTATTAATAAATTTTATTTTTGATTTAAAAATTTTATTTTTTTTTTTTTTATTT
TTTTTTTTTAAATTTAATTTTTTTATTTCTTTTTTAGGTATATTTTTATTTTTTGAAAGA
TTATTTATATTTGTCTTAGTTTTTATAAGTTTAATAATTTTAAGTTTAATTTTTTTAAGT
GAATTTTTATTTAATTTAAAATTTTTAAGAAGTTTATTAGTAATTGTAAGATTATTTTTT
TTTTTAAGAAGAAATTTTTTTTTTTATTTATTTATTTTTTGAATTTTCTTTATTTCCTATT
TTATTAATATTATTAGGTTATGGATATCAAATTGAAAAAATTAATGCTGCTTATTTTTTA
TTTATTTTTACTATATTTTTTTCTATACCATTTTTTTTTTTTTTTTATTAATAATTGATTT
TACTTTGATAACTTTTTTTTTTGATTTTTTTTATAGATGAGAATTATTTTTTTTTTTTTAACT
TTGATATTTATATTAAAATTTCCTGTATATTTTTTTACATTTTTGATTACCTAAAGCTCAT
GTAGAAGCTCCTACTACTGCTAGTATATTATTGGCTGGTTTATTATTAAAATTAGGTACA
GGAGGTTTTATTCGATTATTATTTTCATTTAAATATTTTTTTTTTAGGTAGTTATTTTTTA
TTATCTTTAATTGGAATAATTTTAAGTAATTTTATATGTATTTTTTCAAAGTGATTTAAAA
GCTTTAGCAGCTTTTTCTTCTATTAATCATATAAGATTAGTTTTATTAAGTTTATTATTT
TTAAGTTATGTAAGAATTTTAAATAGGGTAGTTATTATATTCTCTCATGGTTTAATTTCA
ACTTTAATATTTTATTTTATTGGTGAATTTTTTCATTTTAGAGGAGTACGTTTAATTTAT
TATTATAATAGAATTTTTAGAAGAAGTCTTATTTACTCTATTTTAATTTTATTAGTACGA
CTTTATAATAGAGGAGTACCTTTTTCTTTAACTTTTTTTTCTGAATTTAATATTTTTTTA
GTTGGTTTTAATAGTAGTTATTTTTTTTTTTTTTTATATTTTTTATATTTTTTTTTTTAACT
TTTTATTATTGTTTATATTTTATTTCATTAAATTTTTTAGGTAAAAATTTTATTTTAATA
AATTATAATTTAAATATTGGTTTATCATTATTTATAATTGTTATAATATATATAATATTTTT
TGGTTAACTATATTCTTTTAA---------------------------------------
--ATTAAT
TTAATATTATTTTTAGTTTTAATAATGTTTTTTATAATTTTTTTTTTTTATATTTTTTTTTT
TATTTTTATTTTATAGTAATTATTATTAATTGATCTTTTTTTATTTTTTTTTTTTTAATTTT
ACTTTTCTAAAATTAATATTTTTTTTTTTTTTTATTAATTGTTGTTTTAAGAGTTTTATTT

```
TTTGTTAATTTTTATTTAGAGGGAGAGATTTATTTTGTTTATTTTATAATGATTTTATTA
ATTTTTATTTTTAGAATAATTTTTTTAGCTTTTAGAAATAATTATTTAGTACTTATATTA
AGTTGAGATATTTTAGGTATTTCTAGTTATTTTTTGGTATTATTTTATAATAATTGAGAT
AGAAATATTGGTTCTATAAATGTATCTTTAACTAATCGTTTAGGTGATTTTTTTATGTTT
TTTTTTTTTCTTTTTTTATATTTTTAAATTATTTTTTAAGATTTTCTAGATTGATGTCT
TTTATTATTATATTTTTTTTATTAATTATAGGATTTACTAAAAGTGCTCAGTTTCCTTTT
AGAAGTTGGTTACCTAAAGCTATAAGTGCTCCTACTCCTGTAAGAGCTTTAGTACATAGA
AGGACATTAGTAACTGCTGGTTTAATTTTATTTTTAAATTTTAAATTTTATTTATTTTTT
GGAATTTTTTTAAAAATAATTTTTTTTTTTGGTTTATTTACTATATTCTTTGCTAGAATT
ATAGCTTTTTTAGAAGAGGATTTAAAAAAAGTAGTGGCTTTAAGAACTTTATCTCAAATA
GGATTTATAATATTAATTTTAGGTTTAAGTCTTTATTTTTTTAGTTTATTACATTTAGTA
AGTCATGCTTTATTTAAAAGATTTTTATTTATTCAAGTTGGAATTTTGATTTATTTTTCT
TTTGGATCTCAAGATAGTCGTGGTTATAGTGGTTTACAGTTTTTTTTAATTTTATTAAAA
TGAAATTTTATTTTAACTTTATTTTGTTTATGTGGTTTGTTTTTTAGAAGAGGATTATTA
AGTAAAGATATTGTTTTAGAATTTTTTTTTTTTAATAATTTTTTTTTTTATTTTAATTTA
ATATTTTTTTTTTCAGTTTTTTTTACTTTTTTTTTATTCTTTTCGTTTATTTTTAGGATTT
AAAAAAATTTTTTTAAGTTTTATATTTGTAATTTTGAATATTAGTTTTTGTTATACTGGT
TTATTTCTTTATTTATTTTCTTTAGTTGGAATTTCTTTTTTAACTAATAATATTTTAGTA
CTTCCTAGTTTAATTTTAATAGAAGATTTTTTTTCTCCTATTTTTTTTTTGTTTTATATTT
TTTATTATTTTTTTTTTTTAAATTTTAAATTTATTGTGTTTAAATTTAGTTTTATAGTT
GATTATTTTGCTAAAATATTTTCTTTTTTTTTAATAAATTTTAAATTTATTGATTTATTT
TTTAATAATAATTTTATTTATTTTAATTTTATTTTTAAGATTTTTAGTTTTAAATTATTT
AATTTAAATTATAATTTTTATTTTTTTTTTTATATGTTTTATAATTTTTTTTTATTTTATTT
TAA---------------------------------------------------------
------TTGATTTTTATATATTTTTTTATTTTTATTTTATTAGTTTGTTATTTTAGTTTG
ATAAGTTTTGATCCATTTAAAAGTTGTTTATTAATAATTTTAAGTTTAATATTAATGTCT
TTTATTTTAAGTTTTAGTAAGTTTATTTGAGTAAGTTATTTTGTTTGTTTATTGTTTTTA
AGTGGAATTTTTGTTATTTTAATTTATTTTTCTAGTTTAAGGAGATTTTTTTTTTTTTTT
AGAAATTATTTTATTGTAAGTTTAATTTTATTAATAAATTTTGTTTTTATTTTTTATTTTT
AATTTTTTAAATTTTAGATTAAGTTATTTTTTTTTATAATATTTATTTAATATTTTTTTTT
TTTTTTATTTTAGTTTTATTTTATTTTTTAATTTTTTTAAGTTTTTTTTTAAGTTTTAAT
GGAGCTTTACGTAAATATTAA------------------ATTTTTTTTTTTATTTTTAGT
TTATTTTTTTTTTATTTTAAAATTTTATCGTTTTATTTTTATTTTATTAAGTTTTGAGTTT
TTTGTTTTAGGTTTATTTTATTATTTTTCTTTTAATTTTAATAGGTTTTATTTTTTTTTAT
TTTTTATTATTTAGAGTTATTTCTAGAGTTTTAGGAATTACATTAATAATTTTTTTTTATT
AAAAGATATGGAAAAGAAAAAGTTTATTTTTTAA
>B_hofmanni_sample14
---------------------------------TATTGGTTAGAAAGATCTAACCATAAA
GATATTGGAATATTATATTTTATCTTCGGATTTTGATCAGGGTTAGTAGGTACTAGTTTA
TCTATAATTATTCGGTTTGAATTAGCTAAACCAGGAGTTTTTTTGAATAATGGACAACTT
TATAATAGAATTATTACAGCACATGCTTTATTAATGATTTTTTTTATAGTTATACCATCA
ATAGTAGGGGGGTTTGGTAATTGAATATTACCTTTAATGTTAGGTGCTCCTGATATAAGA
TTTCCTCGTTTAAATAATTTAAGGTTTTGACTTCTTCCTGCTTCTTTAATATTACTAATT
GATTCTTGTTTTGTTGATATAGGGTGTGGTACTAGTTGGACTGTTTACCCTCCTTTAAGA
ACTCTTGGGCATCCAGGAGCGAGAGTAGATTTAGCTATTTTTAGTTTACATTGTGCTGGT
GCAAGATCTATTCTTGGTGGTATTAACTTTATATGTACAACAAAAAATTTACGTAGAAGT
TGTATTTCTTTAGAACACTTAAGTTTATTTGTATGAACAATTTTTGTTACAGTTTTTTTG
CTAGTTTTATCTTTACCTGTTTTAGCTGGTGCTATCACTATACTATTAACTGATCGAAAT
TTAAATACTTCTTTTTTTGATCCTAGAATAGGGGGGAACCCTTTAATTTATCAACATTTA
TTTTGATTTTTTGGACACCCCGAAGTTTATATTTTAATTCTTCCTGCTTTTGGGGTTATT
AGGCAAAGAGCTTTGTATTTAACTGGTAAAAAAGAAGTCTTTGGAAATTTAGGAATAGTA
TATGCAATTTTAAGTATTGGTTTAATTGGATGTGTTGTTTGAGCTCATCATATATATACT
GTTGGTATAGATTTAGATTCACGTGCTTATTTTACTGCTGCTACAATGGTTATTGCTGTT
CCAACAGGAGTAAAAGTATTTAGTTGATTAGCTACTTTATTTGGGACTTTAATAATTTTT
CAACCTTTATTATTATGAGTTTTAGGATTTATTTTCTTATTTACTATTGGAGGGCTAACT
GGGGTTATTCTGTCAAATTCTAGTTTGGACATTATTTTACATGATACTTACTATGTAGTT
AGTCATTTTCATTATGTTTTAAGTTTAGGGGCAGTTTTTGGTATTTTTACAGGAGTGTCT
TTATGATGACCTTTATTAGTTGGATGTTTATATGATAAATTATTATTTTCTAGGGTATTT
ATTTTAATATTTATAGGAGTAAATTTAACTTTTTTTCCTCTTCATTTTGCTGGTCTTCAA
GGATACCCACGTAAATATATTGATTATCCAGATATTTATAGTGTTTGAAACATTATTTCT
TCTTTAGGAAGTATATTAAGTGTTTTTTCATTATTTTTATTTTTTTATATACTTATAGAC
TCTATTAGAAGGTTTCGTTTATTAATAAGAGAGGTTTTTAGTAATAATAGTCCTGAGAAT
AGTTTATCAAATTATATTTTCTCTCATGGTTACCAAAGAGAGATTTATTTTATTGTTAAA
TA----------------------------------------------------------
----TAGATTGATTCCATAGTTTTAATTGTAGATTATTATTAGGTGTTCTTATTTTTGTA
AGATTACTATTTATTTTTTTAATTTTTAATTTTAGATTTTTTAAAGGGGAAAAAATAGAA
TATCAATTTGGTGAGCTTCTTTGTAGAGTTTTTCCTACTTTAATTTTATTTTTACAAATA
TTCCCTTCATTAGGGTTGTTGTATTATTATGGTTTAATAAATTTAGAAGCTAATTTAACT
ATTAAAGTTGTTGGGCATCAATGATACTGGAGATACGATTATAGAGGAATTGAGGGGTTA
GATTATGATTCTTATATACTTCCTTCAGATTCCTTAACATTAGGGGATTCACGATTATTA
GAAGTTGATAATCGATGTGTTTTACCTGTTGATTCAAATGTTCGGTTTTGTATTACCTCT
```

TCAGATGTTATTCATGCTTGATCTTTGTCCAGACTTTCTGTAAAATTAGATGCTATGAGA
GGGGTTTTAAGAATTATTAATTATAATTTCCCTGTGATTGGTCTATTTTATGGTCAATGT
TCAGAAATTTGTGGGGCTAATCATAGTTTTATACCTATTGTGGTTGAAGTAACTCTTTTT
GATCTTTTTAAGTTTTGATGTA-TAAATTTTTA---------------------------
----------------------------------TATTTTTTGTGAGTTTAAGGTTAACT
TCTTCTATTGTAGTATTTTTTAAATTTGGTTTATTATTTGGGTTTTTATTAAATTTGATT
ATTATATTTTTTATTTCTTTTCTTTGAGGGAAAGATATTAGTTTTGAAGGAATAAGAGGG
TTTCATAATTTTTTTGTTATAGATGGCTTTAAATTTGGTGTTATTTTGTTTATTTTTAGG
GAATTTATGTTTTTTTTTAGAATTTTTTGAACATTTTTTGATGCTGCTTTAGTCCCTGTC
CATGATCTAGGGGAAATGTGAAGTCCTTATGGATTATTATTAGTTAATCCTTTTGGGGTC
CCTTTACTTAATACTATTATTTTGTTAAGAAGAGGAATCACTGTGACTTGATGTCATTAT
AGGTTATTAAGAAATAATGAAGGAAGAGTAAGTTTACTATTTACTTGTTTTCTTGCTCTT
TATTTTCTTTTAATTCAATTAATAGAATATAAAGAAGCTAGTTTTTCTATCTCTGACGGG
GTATACGGGAGTTTATTTTTTTTATCAACTGGTTTTCATGGTTTTCATGTATTATGTGGA
GGGGTCTTTTTAATTGTTAATTATTTACGTCTTCTGTTATCTCATTTTAGATTTAATCAT
CATTTAGGGCTTGAATTTGCTATTATTTATTGACATTTTGTTGATGTAGTATGATTGTTT
TTGTTTGTTTTTGTTTATTGATGATCATTTTA----------------------------

--TATGTTTTACTGGT
TATTTTTCTTATTCGTTTAGACCTTGTGGTATAATTGAATTTACTTTAATTTTTTCATTA
ATTTTTTGAGGAGCTACTTTTTTATGTTTTTTGAGAAGAGAAAAGCTTTCAATTTATTTT
AGTAAATCAGGAGACATATATTTAAAAACTATGAGTATATTATTAGTTGAGATTGTTAGA
GAGTTTTCACGTCCTGTAGCTTTAACTGTTCGTTTAACTGTTAATTTAATAGTTGGTCAT
ATAATTTGTTCTTCTTTATTTTTACTTTCAATT---AATTTTTTTTTTAGAAGAGTTCTT
TTTTCATTTTTAGCTATTATATTAGAATGTTTTGTTTTTTTTATTCAAAGGTATATTTTT
ACTCGTTTAATTTTTTTATATCTTAATGAATA-ATAAAAACATTAGTATTTTTTGTTAAA
GGGTTACTAGTAAATTTACCATCTAGTAAAAGATTAAGTTTAAATTGAAATTATGGTAGT
CAATTAGCTTTTGTTTTAATATTTCAATTGATTAGTGGATTTTTTTTAGTATTTTTTTTT
AATCCTTCTGGTGGGGAGGCGTTTAGAAGAGTTCAGTATATTATAACAGAGGTTAATTTA
GGGTATATTTTTCGTATTTTACATTTTAATGGTGCAAGACTATTTTTTATATTTCTCTAT
TTACATTTTTTTAAAGGTTTGTTTTTTTTTAGATATCGTTTAAATAAAGTTTGAATGAGG
GGAGTCACACTATTTTTATTGTTTATAATAGAAGCTTTTATGGGGTATGTTTTAGTATGA
GCTCAAATAAGATTTTGAGCTTCAGTAGTAATTACTAGTTTATTAAGGGTTATCCCTATT
TATGGAAGAAAATTAGTTTCTTGAATTTGAGGAGGATTTAGAGTTAATAGAGCTACTTTA
AAATTTTTTTTTGTTTTACATTTTTTGTTACCATGAATATTTTTAATTTTAGTTTTAGCT
CATTTAATTTTACTTCATGATTATGGAAGAACTAATAAACTTTATTGTCACGGAAGGGAA
GGTAAAATTACTTTTTATTCTTTTTATTGATGAAAAGATGGGTTTAATTTATTTATTTGA
TTATTTTTTTTTACTTTTTGTTTTTTATCTCCTTTTGTGTTAGGGGACCCTGAAATATTT
ATTGAAGCTGATCCTATATTAAGTCCAGTACATATTATTCCAGAATGATATTTCTTGTTT
GCTTACGCTATTTTGCGGGCTATCCCTAATAAAGTTTTAGGGGTTGTATTTTTATTATTA
AGTATTATAGTTTTTTTTTTTTTTTTATTTTTAAATATTTCTAATTCTGTTTTTAAAAAA
AGTAATTATTTATTAGTTTTTATTTTTATTTTTATTAGAATTTTTTTGAGTTGGTTAGGG
CAATGTTTAGTAGAACCTCCTTTTTTATTTTTAAGAGGAAGCATTTCTTTTTTTATATTTT
TTTTTTATTCTTTTACTTATATTAAATTTTGTTTTAAGAGATACCCTCTTTAAATTTGTA
GTTAGTATAAATTA--
----TAGTTATTTTAGGGGTAGCTTTTTCAACTCTTTTTGAACGTCATTTTTTTAAGTTTA
AGACAAAATCGTTTGGGCCCTAATAAGTTAAGTTTTATAGGAGTTCTTCAAGCAGGGGTG
GATGGAGTAAAATTATTATCAAAAGAACAATTGTATCCAATTTATTCTTCTGACTTTTTT
TTTGTTTTTACTCCCGCTTGTTTTTTTTTATTTATATTTTTTGAATGAAGATGTCTTCCT
TTTGTTTTTTTTTTTTTTTAATTTTCAATTTTCTTTTTTTATTTTTTTCTTTGTATAGTGGGG
GTCTCAGTTTATTTTATAATTGTGAGAGGAGTATTAAGAAATTCTAAATATTCTTTTTTA
GGGGCAGTTCGTTCTAGAAGACAGAGAGTATCTTTTGAGGTAGCTTTTTCTATTTTAATT
TTTAGTTTAATATTAGTATTTTCTTCAATATCTCTTATTTTTTTTTTTTAGTTTAAAATTA
TTTGTTTTAATTCCTTTATTAATATTTTTAATTTTAGCTGAGTTAAATCGTGCTCCTTTT
GATTTCTCTGAAGGGGAAAGAGAATTAGTTAGAGGATTTAATACTGAACACTCTAGTGTG
AGGTTTATTTTTTTATTTTTAGGAGAATATGGTGTTTTATTATTTTTTTGTACTTTTATA
AATAATTTATTATTCATAGGGAATTGTATAATTTGGGGGATACTTTTTTTTTTTTATTATT
ATTATTCGTAGTGCTTACCCTCGTTTTCGTTATGATTTATTAATAGAGGTTTTTTGAAAA
ATTATTCTTCCTGTTACAATTTTTTTGTTATTTTTTTATATTTTTTCAT-----------
-------------------------TATATTTTCTTTTATTTTTTTTTAAGACTAATAATT
AATAATTATATTTTATGATGGGGAATTTTTTTATTTTTGAGGGTTAGTTTTATTTTAATT
ATAAAATTTAATAATAATTTAAGAGCGAGGTTTATTTATTTTGTTATCCAAGAATGTTGT
GGCTTCTTATTTTTATTAATAAGTTTTATTAATTTACAATGAATTTTTTTATTAATTAAA
ATTGGGGTATCCCCATTTATTTTTTGATTATTTTTATTTTTAGAAAATCTTAATTTGTTT
TGTATCTTTTGATTTCTTACTTTTCAAAAACTTCCTTTTTTACCTATTGTAATAAATTT-
--TTTTTATTCTTTTTTAAAAATTTTTTTTTTTGGAGTATTTTATTTGTATATTTTATTT
TATTTTTTGAAATCATTTAAATTTATTTTAAGGTTAAATTCTGTTGAAAGATTTAGTTGA
ATATTAGTCTTATTTTATTTTTTTTATTTTTCTTTTATTTTTTTGTTTCTTTTTTATATA
ATAAGATTTTTATGTTTATTTTATTCTCCTTCTATTGAAGTTTCATTGTTTATTCTTAGG
GTCCCTTTCACTCCAAGGTTTTTTATTAAGTTTTTTAGGATTTCTTTTATGAGTCCCTTT

ATTGTCTTTTTTTGTTTAATTTTAATTTTAATATTATTAAGATTATTATCTTTATTATAT
CAATTTGCTTGAATCTCTTTATTATTTAAAAATAAATTTATAATAAATAGTTTTTTTTTT
TTTTTTTTTATTATTATTTTTTTTTTTTTGTATTATTA----------------------

----TAGTTTTTCTTTTAGTTTTTTAAGAATATTAATTTTTAGGAGTTATCATTATAATT
GAGAATAACCCCCTTTTAAAATTAGGGGCGTATTTTTTAGTATTAGTGTGTATAATATTC
TTTTTGAGTAGAAGTCTTCTTTTTATATACATATTCTTTGAATTATCTTTTGTTCCTGTA
ATTATTATAATTTTAGGGTTTGGGTACCAAATTGAGAAAATTAATTCTTCTTTTTATCTA
ATATTTTATACAGTTTTTTGTTCTATACCTTTTTTATTTTTTATTTTTAATGTAGATTAC
ACTTTAATTTTTTTTTTTATTGACGGTGAATGAAGATGAGAAATAATAGTTTTAATTATA
TTAATATTTTTAATAAAATTTCCTGTTTATTTTTTACATTTGTGGCTTCCTAAAGCACAT
GTAGAGGCTCCTACAGTCGCTAGTATACTTTTAGCAGGATTACTTTTAAAATTAGGCACA
GGTGGAGTTTTACGTTGTTTAAATAGATTAAATTTTACTTTTATAGGAAGTTTAATAATT
ATTTCTTTTTTAAGAATTATTTTAAGGAACATTATGTGTTTAATTCAAAGAGATTTAAAA
GCATTAGCTGCTTTTTCTTCAATTAATCATATAAGTTTTGTATTATTATTAATTATTTTT
CTTAATAATTCTAGCCTTTGAAGAAGTCTTGTAATTATATTTTCTCATGGTATTATTTCT
ATATTAATATTTTTATTTATTGGAGATTTTTTTCATTTTTCTGGAACTCGTATAGCATAT
TTTTATTCGGGGGGTTTTTTAACTTCTTTAATTATTTGTTTAATAATTAGTACAGTTTGA
TTATTTAATGGTGGAGTCCCTTTTTCTTTATCATTTTTTTCTGAGTTTGTTGGTTTTTCT
AGTCTATTTTTGTGTAATACTTTTTTAATATTTTTTGGAAGTTTATATTTTTTTTTGTCT
TTTTATTATAATTTATATTTTGTTATACTAAATTACTTAGGAGTTGAGGAAAATTTATTT
AATATCTATTTAACTTTAATTAGAGGAAGAATTCTTTTAATAAATGTTAATTTGTTAATT
TTTTTATTTATTTTTTA---

--TAATTAGAAGTTTGAGGTTT
ATTTCGTTTGACCCATTCAAAAGTTGTTTATTAATAATTTTATCTTTTCTTTTATTTTCT
TTTTTTTTAAGGTTAAATTTAGGAGTTTGATGTAGTTATTATATTAGTTTATTATTTTTA
AGTGGGGTTTTTGTTTTATTAATTTATTTTTCTAGTTTAAGAAGGTTTTATTTTTTTTTT
TTAAATTTTTTATTTTTATTATTTTTAATTTGTGGAGGGGTGTTTTTATTTCTAAAGATT
TTTTCTAAGTTAGGAATTTCTTATTATTTTATATCTTTTTTTTTTATAGGGGTTGTTATT
ATTTTAGGAAGTTTATTTTTTTATTTATTTTTTTTAAGTTATATAATAAATTTTCAAGGT
GCTTTACGTAAGTTTTTATTTTTTTTTTTT------------------------------
----TATTTTTTTTTCTAAAATTTTTTCGGTTTATTTTTATTTTACTATCTTTTGAATTT
TTTTTAATAAGAATTTTTTATTGAATTTTGTCTTTTTCTTCTTTTTATTTATTTTTTCTA
TTTTTAAGTTTAGGGGTAGTTTCTAGTTTATTGGGGACAGTTCTTTTAGTTTTATTTTTA
AAAAGATTTGGAAGAGATAAAGTTTTGTTTTTT
>B_seani_sample19

```
------------------------------------------------------------
----------TACTTTATTTTATTTTTGGTTTTTGATCCGGTATAGTTGGTACAAGATTA
TCTTTAATTATTCGTTTAGAATTATCTAAACCTGGTTATTTGTTAAGAAATGGTCAATTA
TATAATAGTGTTATTACAGCTCATGCCTTATTAATAATTTTTTTTATAGTTATACCTTCT
ATAGTGGGAGGTTTTGGTAATTGATTAGTACCATTAATATTAGGTTCCCCGGATATAAGT
TTTCCTCGTCTTAATAATTTAAGATTTTGACTTTTACCTATTTCTTTAATGTTGTTATTA
GATGCTTGTTTTGTTGATATAGGTGCTGGTACAAGTTGGACTGTTTATCCTCCTTTAAGT
ACATTAGGACATCCTGGTAGTAGAGTAGATTTAGCTATTTTTAGTCTTCATTGTGCTGGG
GTAAGTTCAATTTTAGGTGGTATTAATTTTATATGTACTACCAAAAATTTACGTAGTAGC
TCTATTTCTTTAGAACATATAGGTTTATTTGTTTGAACGATTTTTATTACAGTTTTTTTA
TTAGTTTTATCTTTACCAGTTTTGGCTGGGGCTATTACAATATTATTAACAGATCGTAAT
ATTAATACTTCTTTTTTTGATCCTAGAATGGGGGGTAATCCTCTAATTTATCAACATTTA
TTTTGATTTTTTGGCCATCCGGAGGTTTATATTTTAATTCTTCCAGCTTTCGGTATTATT
AGTCATAGTATTTTGTATTTAACTGGTAAAAAGGAAGTTTTTGGTTCTTTGGGGATAGTA
TATGCTATTTTAAGAATTGGTTTAATTGGTTGTGTTGTTTGGGCTCATCATATATATACT
GTTGGTATAGATTTAGATTCACGTGCTTATTTTACTGCGGCTACAATAGTTATTGCTGTT
CCAACTGGTGTAAAAGTATTTAGTTGATTAGCTACTATTTTTGGTTCTGTTATTAAATTT
CAGCCTCTTCTTTTATGAGTTTTAGGATTTATTTTTTTATTTACTATTGGAGGCTTAACT
GGTGTGATTTTATCTAATTCTAGTTTAGATATTATTTTACATGATACATATTATGTTGTT
AGTCATTTTCATTATGTGTTGAGACTAGGTGCAGTGTTCGGAATTTTTACAGGTGTAGCT
CTTTGATGAACTTTAATTATAGGGTTAATTTATGATAAAATTATTTTTAGGGTAGTATTT
ATTTTGATATTTTTAGGTGTAAATTTAACTTTCTTTCCTTTGCATTTTGCCGGTTTACAA
GGTTACCCTCGTAAATATATAGATTATCCAGATATTTATTCTTTATGAAATTTAATTTCT
TCTTATGGGAGGATATTAAGAGTATTTTCTTTATTTTTATTTATTTATATAATTATTGAT
TCTTTTATTCAATTTAAATTATTTATTTATGAGAATAGATCTAATTATAGATCAGAGGGT
TCTTACTCTTCTTATATTTTTAGTCATAGTTATCAAACAGAGATATTTTTTATTGTTAAA
TA----------------------------------------------------------
----TAGATTGATTTCATAGTTTTAATTGTAGTTTAATATTAGGTATTTTAATTTTTGTT
AGTTTACTTTTTTTATTTTTGATTTTTAATTTAAATTTTTTTAAAAGTAAAAAAATTGAA
TATCAATTTGGTGAATTATTATGTAGTCTATTACCTATTTTGATTTTATTATTTCAAATA
ATTCCTTCTTTAAGATTATTATATTTTTATGGTTTAATAAATATCAATTCCAATCTTACT
GTTAAAGTTATTGGGCATCAATGATATTGAAGTTATGATTATAGAGATTTTGATGAATTA
GAGTTTGATTCATATATAAAATCTTTAGATTCTTTAGAATTAGGGGATAATCGTTTATTA
GATGTCGATAATCGTTGTGTGTTACCTTTTAATACAAATATTCGTTTTTGTATTACTTCT
TCAGATGTTATTCATGCTTGAGCTCTTTCTAGTTTATCTATTAAGTTGGATGCTATAAGT
GGTCTTTTGAGAATTTTGAATTATAATTTTCCTTCAATTGGGCTATATTATGGTCAATGT
TCTGAAATTTGTGGTGCTAACCATAGTTTTATACCAATTGTTTTAGAAATTACTTTATTT
AATTTATTTAATAATTGATGTTTGTTATTTA-----------------------------
------------------------------------------------------------
------------------------------------------------------------
------------------------------------------------------------
-------------------TAGATGGATTTAAATTTGGAGTAATTTTATTTATTTTTAGG
GAATTTATATTTTTTTTTAGTATTTTTTGAACATTTTTTGATGCTTCTTTGGTTCCTACA
CATGATTTAGGGGAAATATGAAGTCCTTATGGATTAGTTTTAGTTAACCCATTCGGTGTT
CCTCTATTGAATACTATTATTTTATTAAGAAGCGGAGTTACTGTTACATGATCTCATTAT
AGTTTGTTAAGTAATAAAGATAGATTAATAAGAATATTATTAACTTGCATTTTAGCTATT
TATTTTATTTTAATTCAGCTAATAGAATATAGTGAAGCTAGATTTTCTATTTCTGATGGT
ATTTTTGGTAGAATTTTTTTTTTGTCTACTGGTTTTCATGGTTTTCATGTTTTATGTGGT
GGTTTATTTTTAATATTTAATATATATCGTCTTTATCTTAATCATTTTAATTTTAACCAT
CATTTGGGTTTAGAATTTGCTATCATTTATTGACATTTTGTTGATGTAGTTTGATTATTT
TTATTTGTTTTTGTTTATTGATGATCATTTTA----------------------------
------------------------------------------------------------
------------------------------------------------------------
------------------------------------------------------------
-------------------------------TAATTGAATTTACTTTAAGTTTAGCTCTT
ATTTCTTGATTAAGTACATTTTTATGCTTTATTTCTAGAGAGAAGATTTCTATTTATTTT
AGAAAGGAAGGAGATTCTTTTTTAAAAACTTTAAGAATATTGTTAGTAGAAATTGTTAGA
GAATTTTCTCGTCCTATTGCTCTAACTGTGCGTTTGACAGTTAACATTATTGTTGGTCAT
ATAATTAGCATATCTATTTATTTATTAGTTGAA---AGTTTAGGTGTTAAATATTTTTTT
TTTACTATTTTTGCTATTTTAATAGAATGTTTTGTTTTTATTATTCAGAGTTATATTTTT
TCTCGTTTAATTTTTCTTTATTTAAATGAATA------------------TATTTGTTAAA
AATTTGGTTATTAATTTACCAACAAGTAAAAATTTGAGTTTAAATTGAAATTTTGGTAGA
CAGCTTGGTTTAATTCTTGTATTTCAGATTTTTACTGGTACTTTTTTAGTTTTTTATTAT
TCTTCCGATAGTTTAACTGCTTTTAATAGTGTTCAATATATTATGAATGAAGTTAATTTT
GGATGAATTTTTCGTATTTTTCATTTTAATGGAGCTAGTTTATTTTTTTTTTTTTTTGTAC
ATTCATTTTTTTAAAGGTTTATTTTTTTTTTAGTTATCGTTTAATAAAAGTTTGAGGTAGA
GGATTAATTATTTTTCTTTGTATTATAATAGAAGCTTTTATAGGTTATGTTTTAGTTTGA
GCTCAAATAAGTTTTTGAGCTTCTGTAGTTATTACTAGTTTATTAACAGTTATTCCAATT
TGAGGACCTAAAATTGTAGTTTGAATTTGAAGTGGTTTTAGTGTTTCTGGTGCTACTTTA
AAATTTTTTTTTGTTCTTCATTTTTTGTTACCATGAATATTTTTATTATTGGTAATAATT
CATTTAATTTTTTTACATGATACTGGTAGTACTTCTAAAGTTTTATGTCATGGAGATTTT
```

GATAAAATTAGATTTTTTTCTTTTTATTGATGAAAAGATGGATTTAATATTTTTATTTGA
ATAATATTTTTTATTTTTAGTTTAGTTTATCCATTTATTTTAGGAGATCCAGAAATATTT
ATTGAAGCTGATCCTATAATAAGTCCAGTTCATATTATTCCAGAATGATATTTTTTATTT
GCTTATGCTATTTTACGTGCCATTCCTAATAAAATTTTGGGAGTTATTTTTCTTCTTTTA
AGAATTTTAATTTTTTATTTGTTTTTAGTTAATTTGAATTTTTTAAGTTTAATAAGAATA
TTTAATTATTTTTTAGTGAATTTTTTTATTTTTTTTAGAATTATTTTAAGTTGGCTAGGT
CAATGTATAGTTGAACCACCATTTTTATTTTTGAGTGGTTTGTTTTCTTCTTTATATTTT
ATTTTTATTTTAATAATTTTTTTTAATTATAATTTGTCATATTTATTTTTTAAATTTATG
TATATTATAAATTA--------------------TATTTTTTAGAAGTTTAATTATAATT
TTATTAGTAATTTTAAGTATTGCTTTCACTACTCTTTATGAACGACATTTATTAGGTTTA
AGACAAAATCGGTTAGGTCCCAATAAGGTTTCTTTTTTAGGAATTTTACAGGCAGCTTTA
GATGGTGTTAAATTAATATCTAAAGAGCAAATTTTACCTGTTAATTCTTCTGATATATAT
TTTTTGTTAGTTCCTGGAATTTCTTTTATTTTCATAATTTTAGAATGATTAAGATTACCA
TTTTATTTTTATTTTTTAAATTTTCAATTTCTTTTTTTTTTTTTAATAGTTTTAATTGGG
TTTTCTGTTTATTTTACTATTATTAGTGGTTTAATAAGGAATTCTAAGTATTCTTTTATT
GGTGCTATTCGGTCAAGTAGTCAAAGAGTTTCTTTTGAAATTATATTCTCAATTTATATT
GTAATTTTTATAAATTTTTTAAATTCTTTAAGTTTATTTTATATATTAAATTTAAGTTTT
ATTTTATTATTTTATCCTTATTTATTAATAGTATTAGCTGAATTGAATCGGGCGCCTTTT
GATTTTTCAGAAGGAGAGAGAGAATTAGTAAGAGGTTTTAATACTGAACATTCTAGAGTT
AGTTTTATTTTTTTATTTTTAAGTGAGTATGGTGTTTTAATTTTTTTTAGTATTATAGGA
AGTTTATTATTTTTTAATATGAATTATTTTATATTTTTTTTTATATTTACTTTATTTTTA
TTATTACGTAGAGCTTACCCTCGTTATCGTTATGATAAACTTATAAATTTATTTTGAAAA
TATATTTTACCTGTTATAATTTTTATATTTTATTTTTATTTTTTTTCTTTA---------
----------------------------------TAATTTTTATATTATCTGTTGTTTTT
AATAATTATATTATATGATGAAGTATCTTTTTAATTTTAAATATTAGGTTTTTTTTTTTA
AATAAAATTTTTATAAATGATCTAAGGAATTTATATTATTTTATTGTTCAAGAATTATTA
GGTTTCCTATTTTTATTTTTTGTATTTTTAAATTTTCAATGATTAATTTTAATATTAAAA
ATTGGTATTTCTCCATTTTTTTTTTGGGTTTTTTTTATTGTAGATAATTTAAGAGGTATT
ATACTGTTTTGGTTTTTGACTTTTCAAAAGTTCCTTATTTACCTTTAATTAAGGATTT-
--TTTTATAAATTTTTTATTTTTTTTTATTTTTGGTATTTTGATTATTTATTTTCTATTG
TTTTTTATAAAAAAAATTAAGTTTTTAGTTATATTAAATTCTTTTGAGTCTTTTAGTTGA
ATTTTAATTAATTTATATTTTAGTAGTTTTAATTTTTTAACATTATTTTTTTTTTATAGT
TTTGTATTTATTATATTTATTTTTTTGATAAGAAAAAAAAAT---AAATTATTAAAGTTT
GAATTTATTTTATTTGTATTAAATTTACCACTTATACCTAGATTCTTTTTAAAGTTTTTT
AGTTTAAATTTTATTAGTTATAATTTTTGTTTATTTTTTTTATTTTATTTTTTTATATTT
TTTAATATAATATCTTTTATTTATTATTATTTTATATTTTTTATAAAAAATTTTAATTAC
AAGAAAGATTTAAACTATTGATGAATAATTATAATATTTATTTTTATTTTTATTTTTTGT
ATT---

-------------------------------TATTTAAGTCTTTAAGTTTACATTTTTTT
ATAATTTTAATTATGTTTGTTATTTTTGATTTAGAAATTATTTTTATTTTTGGTTTTCTG
ATAAGTGATATAGAAAGTATTTATTTATTTTTTTTTATTTTTTTTTTTATACTCATTAGT
CTATATTTAGAGTGATTTATAGGTAAATTAATTTGATTATTATAT---------------

-------TACTACTTTTGATTTATATAAGAATTTTAATTTTAAGTCTTATTTTATTAAGA
GAAATTAATTTTAATTTGAAGATATTAAGATTTTTTTTAGTTATTATTAGTTTAATATTT
TTTTTTAGTTCAAATATCTTTTATATTTATTTAATATTTGAGCTTTCTCTGTTTCCTATT
TTATCTATAATTTTAGGTTATGGTTATCAAATTGAAAAAATTAATGCTTCTTATTATTTA
TTAATATTTACTGTTATTTTATCTATGCCCTTTTTTTTTTTTATTTATAATTTGGATTTA
AATAAAAATTTTTTAAATTTTATCTATTTTTTTTCTTGAGAGATAGAATTAATTTTAGTA
TTAATATTTATAGTAAAATTTCCAGTTTATTTTTTACATTTTTGATTACCTAAAGCTCAT
GTAGAGGCTCCTACTAGTGCTAGAATGTTATTAGCAGGGTTATTATTAAAGTTAGGTACT
GGTGGTTTTTTACGTTTGTTATATTCTTTAAATTATCATTTTTTGGGTTTATTTATATTT
ATTTCATTTTTGGGTATAATTTTGAGTAATTTTCATTGTTTAATACAAAGTGATCTTAAA
TCTTTAGCTGCTTTTTCTTCTGTTAATCATATAAGTTTTGTTTTGTTGATTTTAATTTTA
ATAAATATTTTTAGTGTAAAAAGAGGAGTTATTATTATATTATCACATGGGTTTATTTCT
ACATTAATATTTTATTTTATTGGTGAATTTTATCACTTCTCTATTACACGATTAATTTAT
TATTATTCAAGTTTATTTTTAAATTCATTATTTTTTTGTTTAATAATTAGTTTTACTTGA
TTATTTAATTCTGGTATTCCTTTTTCTATTACTTTTTATTCAGAATTTATTTCTTTTTTA
GTTATTTTTAATTTTAATATAGCTTTTTTTTTTTTATCTTTTTTATATTTTTTTATTTCT
TTTTACTATAGTTTATATTTTATTGTGTTAAATTTTGTTGGTAAAGAAAAATTGAATATA
AATATAAATTTGGTTATTTATAGATATTTTTTTTTACTTTTAAATGTTAACATTTTTTTT
TTTGTATTTTTTTTTTA---

----------------TATTCTTTTTTTTGTTGTTAATTGTTGTTGTTAGAATTTATTTT
TTTTTAAATTTTTATTTGGATAAGGAAGTATATTTTAATTATTTTTTAATAATTTTATTA
ATTTTTATTTTTAGTATAATTTTTTTAAATTTTAGTAATAATTATTTAATTATAATATTG
AGATGAGATACACTTGGTATTTCTAGATATTTTTTAGTTTATTTTATAATAATTGAGAT

AGTAATATTGGTTCTATAAATGTTTCTTTAACTAATCGTTTGGGAGATTTTTTTATATTT
TTTTTTTTTAGTTTTTTTTTATTTTTAAATTTTAATTTTAAGTTTATAAATTTTTATTTT
TTTAGTTTAATATTAATGTTAATTATTATAGGTTTTACTAAAAGAGCACAGTTTCCTTTT
AGAAGTTGATTACCGAAAGCTATAAGAGCACCTACTCCTGTTAGAGCATTAGTTCATAGA
AGTACCTTAGTTACGGCTGGTTTAATTTTATTTTTGAATTTTAGTATATTTATAATTAGT
TATGTATTTATTTTAGTTATGTTATTTTTTGGTTTATTAACTATATTAATAGCTAGTGTT
ATATCTTTGAAAGAAAAGGATTTAAAAAAGGTTGTAGCGTTAAGAACTTTATCTCAAATA
GGTTTTAGAGTTATAATTTTAGGTTTAAATTTAAATTATTTTTGTTTAATACATTTAATT
AGTCATGCTTTATTTAAAAGTTGTTTATTTATTCAACTTGGTTTGATTATTTATTTTATA
TATGGTCAGCAAGATAATCGTTTTTATAGTGGTTTAAATAAATTTTTTAATTTTATTAAA
TGACAGTTTTTATTAACTTTGTTTTGTTTATGTGGATTATTTTTTAGAAGTGGCATAATA
AGGAAAGATATAATTTTAGAATTTTTTTTTTTTTTTTAATAAAAAAAATTTTTTTAATATTT
ATATTTTTTTTTAGTGTAATGATGACTTTTTATTATTCTTATGTTTTGTTTTTGGGGTTA
ATAAAAAAATAACAACATTATTTTTGTTTTTTATTTAAAAAACTATAATATTTTTATATCT
TTTATTTTATTTTTATTGTCTTTAGTTGGATTAATATATATTAATTTAAACATAATTATT
TTTCCTTCATTAGTTTTAATGATTGATTTTTATGTTCCTTTATTTTTTTTTATATTTTTT
TTTTTTCTATTTTTTTATTTTAAGATAAAAAATATAATTATTTTAAAATATAGTTTTATA
AATGATTATTACGCCAAGGTTTATTCTTTTTTTATTTTTAATTTAAAATTTCTTGATAAT
TTTTTCAATAAAATTTTTTTATTTTTATTTTTAAAAAATTTAATGAATAAATTAAGTTTT
ATTTTTGTTAATAATTCTTTTTTTGTTTTTTTTTATTTTATTGATTATTTTTATATTTTA-
----

---TATTTTTTAGA
CTAATATTTTTCATTTTTAAATATTATCGTTTTATATTGATTTTAATTTCTTTTGAATTT
TTTATAATAGGTTTATTTTTTTTATTTCTCTTTTAAAACTAATTTGTTTTTATTTTTTTTAT
TTTTTATTTTTTAGTGTTTTATCAAGTGTATTGGGTATAGTTTTAATAATTTATTTAATT
AAAAGTTTTGGTAAAGATAAAATTTTATTTTA-
>B_penai_sample40A

----------TATTATATTTTATTTTTGGTTTTTGGTCAGGTATATTGGGTACAAGTTTA
TCTTTAATTATTCGTTTTGAATTATCAAATCCTGGTTATTTTTTAAATAATGGTCAATTA
TATAATTCTGTTATTACTGCTCATGCTTTATTAATAATTTTTTTTATAGTTATACCTTCT
ATAGTTGGAGGTTTTGGAAATTGACTTGTTCCTTTAATATTAGGTTCTCCTGATATAAGG
TTTCCTCGTTTAAATAATTTGAGATTTTGATTATTACCAGTTTCTTTAATATTATTATTA
GATGCTTGTTTTGTTGATATAGGTGCTGGTACTAGTTGAACTGTCTATCCTCCTTTAAGA
ACTTTAGGGCATCCTGGGAGAAGAGTGGATTTAGCTATTTTTAGGCTTCATTGTGCAGGT
GTAAGTTCTATTCTTGGTGGTATTAATTTTATATGTACAACAAAAAATTTACGTAGTAGT
TCTATTTCTTTAGAACATATAGGTTTATTTATTTGAACTATTTTTATTACTGTTTTTTTA
TTAGTTTTATCTCTTCCTGTTTTAGCTGGTGCAATTACTATATTATTAACTGATCGTAAT
ATTAATACTTCTTTTTTTGATCCTAGAATAGGTGGTAATCCTTTAATTTATCAACATTTA
TTTTGATTTTTTGGGCATCCAGAAGTTTATATTCTAATTTTACCAGCTTTTGGTATTATT
AGACATAGAATTTTATATTTAACTGGTAAAAAAGAAGTTTTTGGTTCTTTAGGTATAGTT
TATGCTATTTTAAGAATTGGTTTAATTGGTTGTGTGGTGTGGGCTCACCATATATATACT
GTTGGGATAGATTTAGATTCTCGTGCTTATTTTTCTGCTGCTACTATAGTTATTGCTGTA
CCCACAGGAGTAAAAGTTTTTAGTTGATTAGCAACTTTATTTGGTTCTGTTATAATTTTT
CAACCTATTTTATTATGAGTTTTGGGTTTTATTTTTTTTATTTACTGTGGGTGGTATTACT
GGTGTTATTTTATCTAATTCTAGTTTAGATATTATTTTACATGATACATATTATGTAGTT
AGTCATTTTCATTATGTATTAAGTTTAGGGGCTGTTTTTGGTATTTTTACAGGAGTTTCT
TTATGATGAACTTTTATTTTAGGTTTAGTTTATAATAAATTTATTTATTCTTCTGTTTTT
TTTCTTATATTTATTGGGGTAAACTTAACTTTTTTTCCTTTACATTTTGCTGGTCTACAA
GGTTATCCTCGTAAATATATAGATTACCCTGATATTTATTCTTTATGAAATATTGTTTCT
TCTTTTGGAAGTATAATCAGTGTTTTTTCTTTATTTTTATTTATTTATTTAATAATTGAT
TCTTTTCTAAATTTTAAATTATTTTTAGCTGATTATAGAACTAATTATAGTGCTGAGAAT
TCTTTGTCTTCTTATGTTTATAGTCATAGTTTTCAAAGTGAAATATTTTTTATTGTTAAA
TA--
----TAGATTGGTTTCATAGATTTAATTGTAGTCTGATATTAGGTGTTTTATTATTTGTA
ATTTTTATTTTTTTATTTTTAATTTTTAATTCTTTAATTTTTAAAAGAAAAAAAATTGAA
TATCAATTTGGGGAATTACTTTGTAGTCTCTTTCCTACTTTAATTCTTATTTTTCAAATA
ATTCCTTCTCTTAGTTTATTATATTTTTATGGTTTAATAAATATTAATCCAGAAATTTCT
GTTAAAGTTATTGGACATCAATGATATTGAAGTTATGATTATAGAGATTTAGATAGTGTT
GAATTTGATTCTTATATAAAATCTGTAGATAATTTAGAATTAGGAGATTTACGTTTATTA
GATGTTGATAATCGTTGTATCATTCCTTTTGATATTAATATTCGTTTTTGTGTAACTTCT
GCTGATGTTATTCATGCTTGAGCTCTTTCTAGTTTATCTATTAAATTAGATGCTATAAGT
GGTATCTTGAGAATTTTTAATTATAATTTTAATTCTGTTGGTTTATTTTATGGACAATGT
TCAGAGATTTGTGGAGCGAATCACAGTTTTATACCTATTGTTCTTGAATTAACTCTTTTT

GATTTATTTAAAAATTGATGTTTATTATTTTA----------------------------

-------------------TAGATGGATTTAAATTCGGTGTAATTTTATTTATTTTTAGT
GAATTTATATTTTTTTTTAGTATTTTTTGAACTTTTTTTGATGCTTCTTTAGTTCCTACT
CAAGAATTAGGTGAAATATGAAGTCCCTATGGTGTAATTTTAGTTAGTCCTTTTGGTGTG
CCTTTTTTAAATACTATTATTTTATTGAGTAGGGGGGTGACTGTTACTTGGTCACATTAT
AATTTATTAAGTAATAAAGATAGTTTTATTAGTTTATTATTTACATGTTTATTAGCGTTA
TATTTTATTTTGATTCAAGGCATAGAATATAAAGAAGCAAGATTTTCTATATCTGATGGA
ATTTTTGGTAGAATTTTTTATTTATCTACTGGATTCCATGGGTTTCATGTTATTTGTGGT
GGTATTTTTTTAGGTTTTAATCTTTATCGTTTAATACTTAATCATTTTAACTTTAATCAT
CACTTAGGGTTAGAATTTGCGATTCTTTATTGACATTTTGTTGATGTAGTTTGATTATTT
TTATTTGTATTTGTTTATTGATGATCTTTTTA----------------------------

-------------------------------TATTAGAATTTACTTTTTTTATTTCTTTA
ATTTTTTGGTTAAGAACTTTTTTATGTTTTTTAACAAGTGAAAAAATTTCTGTTTATTTT
AGAAAAGGAGGAGATAATTTTTTAAAGACTTTTAGTATATTTTTGATTGAAGTAGTAAGA
GAATTCTCTCGTCCTATTGCTTTAACTGTGCGTTTAACTGTGAATATTATGGTTGGGCAT
ATAATTAGGATATCTTTATATTTATTAGTAGAA---TCTAGTGGTTTTAAATTTTATATT
TTAACTGTATTTGCTATTTTAATAGAGTGTTTTGTTTTTATTATCCAAAGTTATATTTTT
TCTCGTTTAGTTTTTTTATATTTAAATGAATAT---------------------------

----------------TAATTTTACAAATTTTTACTGGTACTTTTTTAGTTTTTTATTAT
ACATCTGATAGGAATTTAGCTTTTGATAGAGTACAATATATTATAACTGAAGTTAATTTT
GGTTGAATTTTTCGTATTTTTCATTTTAATGGGGCTAGATTATTTTTTATTTTTCTGTAT
TTACATTTTTTTAAAGGTTTATTTTTTTCAAGGTATCGTTTATTTAAAGTTTGAGGTAGA
GGATTAATTATTTTTTTTATGTATTATAATAGAAGCTTTTATAGGGTATGTTTTAGTTTGA
GCTCAAATAAGATTTTGAGCTTCCGTAGTTATTACTAGTTTATTGAGTGTTATTCCTATT
TGAGGAATAAAAATTGTTGTTTGAATTTGAAGAGGGTTCAGAGTTTCAGGTGCTACATTG
AAGTTTTTTTTTGTTTTACATTTTCTATTACCTTGATTATTTTTAATTATTATTATACTT
CATTTAATTTTTCTTCATGATTATGGTAGAACTTCTAAAGTTTATTGTCATGGTGATTAT
GATAAAATTAGGTTTTATGTTTTTTATTGATGAAAAGATAGATATAATTTTTTATTTTTT
TTTTTTTTTTTTATTTTTAGTTTAGTGTATCCTTTTATTTTAGGCGATCCAGAAATATTT
ATTGAGGCTGATCCTATAATAAGTCCTGTACATATTATTCCTGAATGATATTTTTTGTTT
GCTTATGCAATTTTACGTGCTATCCCTAATAAAATTTTAGGAGTAATTTTTTTATTATTA
AGTATTTTAGTATTTTATTTATTTTTATTAAAATTGAATTTTTTTTCTTTGATAAAAAAA
ATTAATATTTATTTAGTAAATTTTTTTATTTTTGTAAGTTTAATTTTAAGATGATTAGGT
CAGTGTTTAGTTGAACCTCCATTTTTATTTTTAAGTGTTTTATTTTCTTTTTTTTATTTT
TTTGTAATTTTTATAATTTTATTTTTTTTATTTATTAAGAATATTTTTATTTGAGTATATA
ATTATTATAAATTA--

--------------------------TATATTTTTTTATTTTTTTTTTAGTTTATTAGTG
AATAATTATATTTTTTGATGAAGTATATTTTTAATATTAAAT-TTATGTTTTTTTTTTTA
AATAAAATTTTTTTAAATGATTTAGTAAATTTATATTATTTTATTTTACAAGAGTTTCTT
GGTTTAATTTTTTTATTCTTTATTTTTATAAATTTTCAGTGATTAATTTTAATAATTAAA
ATAGGTGTTTCACCTTTTATTTTTTGGTTGTTTTTTATTATTGAGAATTTAAATAATTTT
ATGTTGTTTTGATTTTTAACTTTTCAAAAATTACCTTTTATTCCTTTAATTAAGGATTT-
--TTTACTAAATTTTTTATTTTTTTTTT--TTTTGGTTTATTATTAATTTATTTTTTAATT
TTTTATTTAAAAAAAATTAAATTTTTAATTATAATAAATTCTATAGAATCTTTTAGATGA
ATTATTTTATTAATTTTTTATAGAAGTTTAAATTATTTTTTTATTTTTTTTTTTTTATTTT
TTTAGATTTTTATTTTTATTTTTATTTATCAATTATTCTAAA---AGTATTTATAGTTTG
GAATTAATTTTATTTTTTTCTAAATTTTCCTTTGATACCGAGATTTTTTATAAAATTTTTG
ATTTTGTGTTTTTTTATTAAAAAGTTTTTTTATTATTATTTTATTATTAATTTTAATAATT
TTTAGAGTCTATACTTTTTTTATTATTTTTTTGT--------------------------

```
----------------------TAATTGTTTTTTTTTTAAGTTTTTTTTTTGTTTTTTTG
TTTTTTTTTATTAATATATTTTTATCTTTTAAATTTTTTACTTTTAATAAAATTTCAGTT
TTTGAAAGTGGTTTTGTTAGGATTGGAAAAATTTTTAAATCTTTTAGTTTACATTTTTTT
TTAATTATAATTATTTTTGTTATTTTTGATTTAGAGTTAATTTTTATTATTGGTTTTGTT
TTAAGAGATTTTGAAGGTGATTTATTATTTTTTATTTTTTTTTTTTTTATTATTTTAAGT
TTATTTATTGAATGATTTTTAAATAAATTAGTTTGATTAATTTTT---------------
------------------------------------------------------------
------------------------------------------------------------
-------------------------TAAGAGTTATAATTTTAGGGTTAGTTTTAATAAGG
GAATTAAATTTTAATATTAAGTTTTTAAGTTTTATTTTGGTTGTTATTAGAATTTTTTTT
TTTTTTAGTAATACATTATTTTTTTTATATTTTTTTTTTTGAAATTTCTATTTTTCCTATT
TTGATGATAATTTTAGGATATGGTTATCAAATTGAAAAAATTAGGGCTTCTTACTATCTT
TTAATATTTACTGTATTTTGTTCAATACCATTTTTTTTATTTATTTTAAATCTTGATTTT
AATAAAATTTTAAATTTTATTGATTTTTTTTTTTTCTTGAGAATTAGAATTAATTTTAATT
TTGATATTTTTAGTTAAATTTCCTGTTTATTTTTTACATTTTTGATTACCAAAAGCTCAT
GTAGAGGCTTCTTCTTCAGCAAGAATATTTTTAGCTGGTTTATTATTAAAATTAGGTACT
GGAGGATTTTTACGACTTTTAAAGAGTTTAAATTACAATTTCTTAGGTTATTTTATTTTT
ATTTCAATTTTAGGTATAATCTTTAGTAATTTTAATTGTTTATTTCAAAGTGATTTAAAA
TCTTTAGCTGCTTTTTCTTCTATTAATCATATAAGTTTAGTACTTTTTATATTAGTTATA
ATTAATTTAAATAGTGTTAAAAGTAGTGTTTTAGTAATATTATCTCATGGTTTTATTTCA
ACATTAATATTTTATTTTATTGGTGAAGTTTATCATTTTAATTTAACTCGTTTAAATTAT
TATTATTCTAGAATTTTTATTTTTAGAATTTTTTTTTGTTTATTAGTAATATTTACTTGA
TTATTTAATTCAGGGGTACCTTTTTCTGTTTCTTTCTATGCTGAATTTATTACTATAGTT
ATTATTTTTAATAAAAATTTAATATTTTTTTTTTTTTATTTTTATTTATTTTTTTGTTTCT
TTTTATTATTGTTTATATTATATTAGTATAACTTTTTTAGGTAGAAGTTTTTTCAGT---
------------------------------------------------------------
------------------------------------------------------------
------------------------------------------------------------
------------------------------------------------------------
------------------------------------------------------------
-------TAAAAATATTTTTTTTTTTTTTTTATTATTAATTGTGGTTTTAAGAGTATTTTTT
TTTTTGAATTTCTATTTAGAAAATGAGGTTATATTTAATTATTTTTTTAATTGTTTTATTA
ATTTTTATCTTAAGTATAATTTTTCTTAATTTTAGAATAAATTATTTAATATTAATATTA
AGTTGAGATGTTTTAGGTATTTCTAGATATTTTTTAGTTTTATTTTATAATAATTGGGAT
AGAAATATTGGTTCTATAAATGTTTCTTTAACTAATCGTTTGGGAGATTATTTAATATTT
TTTTTTTTTTTTTTTTTTTTTTTTTTTTAATTTAAGAATGAATTTTTTTTCTTATTATTTA
TTTTTTTATTTTTTTTTTTTTAATTTTTATAGGTTTTACAAAAAGAGCTCAGTTTCCTTTT
AGTAGATGATTACCTAAAGCTATAAGTGCTCCTACTCCTGTAAGGGCTTTAGTTCATAGT
AGAACTTTAGTTACTGCTGGTATAATTTTATTTTTAAATTTTTTTTTAATTTTAGAAAAT
TTTTTTTTTTTTTTTTTTTTTATTAATTTTTGGTTTATTTACTATATTAATAGCTAGTTTT
ATATCTTTAAAAGAAAAAGATTTAAAAAAAGTAGTTGCATTAAGAACTTTATCTCAAATA
GGGATAAGAGTTTTAATTTTAGGTTTAAATATAAATTATTTTTGTCTTTTACATTTAGTT
AGTCATGCTTTATTTAAAAGTTGTTTATTTATCCAAGTAGGTGTTTTTATTTATTTTTCT
TTAGGACAACAAGATAGACGTTTTTATGGAGGTTTATTAAGTTTCTTTAATTTTATTAAA
TGACAAATAATAATTACTTTATTTTGTCTTTGTGGTTTATTTTTTAGAAGTGGTTTAATT
TCTAAGGATTTAGTTTTAGAATTTTTTTTTAATTTAAGTAATTTAATAATTTTAAAAATT
TTTTTTTTTTTTTCTATTTTAATAACTTTTTTTTATTCTTTTATTTTATTTAAAGGTCTA
TTAATAAATTCTATATTTTATTTTATTTATTATTTAAATAATTTTAAATTATTTATATCT
TTTTTTTTATTTATTTTTTCATGTATAGGTTTAATATTTATTTATTTAAATTTAATTATT
TTTCCTTGTTTTATTTTATTTTTAGATTGTTTTTATCCTTTATTATTTATTTTTTTTTTT
TTTTTTTA----------------------------------------------------
------------------------------------------------------------
------------------------------------------------------------
------------------------------------------------------------
------------------------------------------------------------
------------------------------------------------------------
------------------------------------------------------------
------------------------------------------------------------
------------------------------------------------------------
------------------------------------------------------------
------------------------------------------------------------
------------------------------------------------------------
------------------------------------------------------------
------------------------------------------------------------
------------------------------------------------------------
------------------------------------------------------------
---------------------------------
>B_gerbari_sample12A
------------------------------------------------------------
----------TATTGTATTTTATTTTTGGTTTTTGATCAGGTATAGTAGGAACAAGATTA
TCTATAATTATTCGTTTAGAATTGTCAAAACCTGGATTTTTAATTAATAATGGTCAATTG
```

TATAATAGTATTATTACTGCACATGCTTTATTAATAATTTTTTTTATAGTTATACCTTCT
ATGGTAGGAGGATTTGGTAATTGATTGTTGCCTTTAATATTAGGCTCTCCTGATATAAGA
TTTCCACGTTTAAATAATTTAAGGTTTTGATTGTTACCTACGTCATTAATATTATTATTA
GATGCATGTTTTGTTGACATAGGTAGTGGGACAAGATGAACTGTTTATCCTCCTTTAAGA
ACATTAGGTCACCCTGGTAGAAGGGTTGATTTGGCTATTTTTAGTTTACATTGTGCAGGT
ATTAGATCAATTCTAGGAGGAATTAATTTTATATGTACTACTAAAAATTTACGTAGGAGT
TCTATTTCTTTAGAACATATAAGTTTATTTGTTTGAACAATTTTTGTTACAGTATTTCTT
TTAGTTTTATCTTTACCTGTATTAGCAGGAGCTATTACTATACTTCTTACTGATCGTAAT
ATTAATACTTCTTTTTTTGATCCTAGTATAGGAGGTAACCCATTAATTTATCAACATTTA
TTTTGATTTTTTGGTCATCCTGAAGTTTATATTTTGATTTTACCAGCTTTTGGTATTATT
AGTCAAAGTACTCTTTATTTAACTGGTAAAAAAGAGGTTTTTGGTTCTTTAGGTATAGTT
TATGCTATTTTAAGTATTGGTTTAATTGGTTGTGTAGTATGAGCTCATCATATGTATACA
GTAGGTATAGATTTAGATTCTCGAGCTTATTTTACTGCAGCAACTATAGTGATTGCTGTT
CCTACAGGAGTAAAAGTTTTTAGCTGATTAGCTACTCTATATGGTTCAATTTTAATTTCT
CAACCACTAATATTTTGAGTTTTAGGTTTTATTTTTCTTTTTACAATTGGTGGTTTATCT
GGTGTAATTTTATCTAATTCTAGACTTGATATTATTTTACACGATACTTATTATGTTGTT
AGTCATTTTCACTATGTTTTAAGTTTAGGTGCAGTTTTTGGTATTTTTACAGGTATTTCT
TTATGATGAACTTTAATTATAGGTCTTGTTTATGACAAAGTTTTATTTAATTCTATATTT
ATTTTAATATTTATTGGGGTAAATTTAACTTTTTTCCCTTTACATTTTGCTGGATTGCAA
GGGTACCCTCGTAAATATATAGATTATCCTGATATTTATTCATTATGAAATATTGTTTCT
TCTTTTGGTAGAATATTAAGGATGTTTTCTCTTTTTGTTTTTATTTATATTTTATTAGAT
TCATGTAATAATTATCGTGTTTTTTTAAATGATATAAGATTAAATTATAGTCCAGAAAAT
ATATTAACTGGTTATATTTTTAATCATAGTTATCAAAATGATGTTTATTTTATTGTAAAA
TTA---
----TAGATTGGTTTCATAGTTTTAATTATAGTTTGTTATTTGGGGTATTAATATTTGTT
AGGTTATTATTTTTTTTTTTAATTTTTAGTTTTAATTTTTTTAAAAGAAAAAAAATTGAA
TATCAGTTTGGTGAATTATTATGTAGAATTATACCTACTTTAATTTTAATTTTACAAATA
ATTCCTTCTTTAAGTTTATTATATTATTATGGTTTAATAAGAATTGATTCTAATTTAACA
ATTAAAGTTATTGGACATCAATGATATTGGAGATATGAATATAGAGATTTTGAAAATTTA
GAATTTGATTCTTACATAAAAACTTTAGATTCTTTAAATTTAGGAGATATACGGTTATTA
GATGTTGATAATCGTTGTATTTTGCCTTTAGATTTAAATATTCGTTTTTGTGTAACTTCA
GGAGATGTAATTCATGCTTGAACTTTAAATGGTTTGTCAATTAAACTTGATGCTATAAGT
GGGATTTTAAGTATTTTTAGATATAATTTTCCTTTAGTTGGTTTATATTATGGTCAATGT
TCTGAAATTTGTGGTGCTAATCATAGATTTATACCAATCGTAGTTGAAGTAACTTTATTT
AATTTATTTAAGAGTTGAAGTTTATTATA----------------------CATAATTTT
CATATTTTAAGATTATCTAGTTATCCTTTTATGGTTTTTTTAAATACTTTAGGTTTAACT
TCTTCACTTGTAGTGATGTTTAAATTTAATATTTTTATGAGTGTATTTTTAATACTCTTA
ATTTTATTTTTTATTATAATAATTTGAATGAAAGATATTTCTTATGAAGGTTTAAGAGGG
TATCATAATTTTTTTGTTATAGATGGTTTTAAATTTGGTGTAATTTTATTTATTTTTAGA
GAATTTATATTTTTTTTTAGAATTTTTTGAGTTTTTTTTGATTCTTCTTTAGTACCAAAT
TCTGATTTAGGTGAAGTTTGGAGACCTATAGGTTTAATTT--------------------

---------------------ATAGAGTATAAAGACGCTAGTTTTTCTATTTCTGATGGA
ATTTTTGGTAGAATTTTCTTTCTTTCTACAGGATTTCATGGTTTTCATGTTTTTTGTGGA
GGAGTATTTTTATTTTTGAATTTATTACGTATTATGAAAAGACATTTTAATTTTAATCAT
CATTTAGGTTTAGAATTTGGAATCATTTATTGACATTTTGTTGATGTAGTTTGATTATTT
TTATTTGTTTTTGTTTATTGATGG------------------------------------

-------------------------------TGATTGAGTTTACTTTATTTGTATCTTTG
ATTTCTTGGTTTAGAACCTTTTTATGTTTTATTAGAATAGAAAAAATTTCAATTTATTTT
AGAAAATCTGGTGATAGATTTTTAAAAACGTTAAGAATATTAATAGTAGAATTAGTAAGA
GAGTTTTCTCGTCCTATTGCTTTAACTGTGCGTTTAACTGTTAATATTATAGTAGGACAC
ATAATTAGAATATCAATTTATATATTTTTAGAA---AATGTTTCATATTTAGGTTTAATT
TTATATATTATAGCTATTTTAATAGAATGTTTTGTTTTTATTATTCAGAGTTATATTTTT
TCTCGTTTAATTTTTCTTTATTTAAATGAGTA-ATAAAAATTTTATTAAATTTTATAAAG
GGTTTACTGGTTAATTTACCTACAAGAAAAACTTTAAGTTTAAATTGAAATTATGGTAGA
CAATTAGCTTTAATTTTAATTTTTCAAATTCTAACAGGATTTTTTTTAGTTTTTTATTAT
ACTCCTGAGAGACTAATATCTTTTGATAGAGTTCAATATATTATAAATGAGGTTAATTTA
GGTTGAATACTACGTATTTTTCATTTTAATGGGGCAAGTTTATTTTTTATTTTTCTTTAT
TTACATTTTTTTAAAGGTTTATTTTTTTCAAGTTATCGTTTGAGGAAAGTTTGAATAAGA
GGAATTTTATTATTCTTATTAATTATGATAGAAGCTTTTATAGGTTATGTTTTAGTATGA
GCACAAATAAGGTTTTGAGCTTCAGTTGTAATTACTAGATTACTAAGAGTTATTCCTATT
TGAGGAAGTATAATTGTAACTTGAATTTGAAGAGGTTTCAGAGTAAGTGGTGCAACTTTA
AAATTTTTTTTTGTTTTACACTTTATTTTACCTTGAATATTTTTAATCTTAGTTTTATTA
CATTTAATTTTACTTCATGATTATGGTAGAACGTCAAAAATTTTAACTCATAGAGATTTA
GAAAAGATT---


```
------------------------------------------------------------
------------------------------------------------------------
------------------------------------------------------------
------------------------------------------------------------
------------------------------------------------------------
------------------------------------------------------------
------------------------------------------------------------
------------------------------------------------------------
------------------------------------------------------------
------------------------------------------------------------
------------------------------------------------------------
------------------------------------------------------------
------------------------------------------------------------
------------------------------------------------------------
------------------------------------------------------------
------------------------------------------------------------
------------------------------------------------------------
------------------------------------------------------------
------------------------------------------------------------
------------------------------------------------------------
------------------------------------------------------------
------------------------------------------------------------
------------------------------------------------------------
------------------------------------------------------------
------------------------------------------------------------
------------------------------------------------------------
------------------------------------------------------------
------------------------------------------------------------
------------------------------------------------------------
------------------------------------------------------------
------------------------------------------------------------
------------------------------------------------------------
------------------------------------------------------------
------------------------------------------------------------
-------------TATTATTATTTGTATTTTTAATTTTAAGATATCTGTTAATTATAATT
TTTTTTTTTATAAATTTAGTTTTAAGGTTTAAATTTGGAAATAAAAATAAAATTTCAACT
TTTGAAAGAGGGTTTATGAGAGTTAGAAAAGTTTTTAAATCTTTTAGTATTCATTTTTTT
TTAATTTTAGTTATATTTATTGTTTTTGATTTAGAAATTATTTTTATTTTGGGATTTTTA
ATTACTGATAGAGAAAGTTTAATTTTTTTTTTTTTTGTTTTTATTTATAATTTTTA----
------------------------------------------------------------
------------------------------------------------------------
------------------------------------------------------------
------------------------------------------------------------
---------------------------------------------------ATTTTTTTT
TTTACTAGTAATAATATTTTTTTTTTATATTTTTTATATGAATTAAGATTTTTACCCATT
ACTATTATAGTTTTGAGTTTTGGTTATCAGATTGAAAAAATTAGAGCATCTTATTATTTA
TTATTTTATACTTTAATTTGTTCCATACCATTTTTTTTTTTTATTTTAAATTTGTTTAGT
TTTAATTTAAGTAGATTAAATTTTTTTTTTTTTTCTTGAGAAATAATATTACTTTTAAGA
TTTTTATTTATAATAAAATTTCCTGTTTTTTTTTTTCATTTATGACTTCCAAAAGCTCAT
GTAGAAGCGTCTACTACAGCTAGAATATTACTAGCTGGATTGCTATTAAAATTAGGAACT
GGTGGTTTTTATCGGTTTATTAATTTGTTTAATTTTTTTTTTTTAAATTATTATATTATT
TTATCATTACTCGGTATTATTTTAAGAAATTTTTATTGTTTATTTCAAAGTGATTTAAAA
TCTCTTTTAAATTTTAAATTTATC------------------------------------
------------------------------------------------------------
------------------------------------------------------------
------------------------------------------------------------
------------------------------------------------------------
------------------------------------------------------------
------------------------------------------------------------
------------------------------------------------------------
------------------------------------------------------------
------------------------------------------------------------
------------------------------------------------------------
------------------------------------------------------------
----------------------------------------------TAAGAGTTTTATTT
TTTTCTAATTTTTATTTGGTGAAGGAAAATTTTATAGTTTATTTTTTAATAAATTTATTA
ATTTTTATTATAAGGATAATTTTTCTAAATTTTAGAGATAATTTTTTTTTTTTAATTTTA
AGTTGAGATTTTTTAGGTATCTCAAGTTATTTTCTTGTATTATTTTATATTAATTGAGAG
AGAAATTATAATTCTATAAATGTTTCTTTAACTAATCGTTTAGGTGATTATTTTATATTT
TTTTTTTTCTTTTTTTTTTTTTTTTTTTTTTA----------------------------
------------------------------------------------------------
```


---TATTAAGATTT
TTTTCTTTTGATCCTTTAAAGAGTTGTTTGTTAATGATTTTAAGTTTAATTTTAATAACT
TATTTTTTTAGTAATAGTATAAATATTTGATTTGGTTATTTTATTAGTTTGTTATTTTTA
AGTGGAATTTTTGTTATCTTTATTTATTTTTCTAGATTAAGAAGATATTATTTTTTTTTT
TTTAATAAATTTAATTTAGTTTTTTTTTTTTTTTTTTTATGTATTTTTAATGATAAATTT
AAAATTTA--

----------------------------------AATTTATATTTTTTTTTTTTTTTGGT
TTAATATTTTTTATTATTAAGTTTTATCGTTTTATTTTTATTTTAATTTCTTTTGAATTT
ATAATAATAGGTTTATTTTATACTTTTAGTTTATGTTTTTCTTCTTTTCTTTTTTTTTTT
TTTTTGAATTTTAGTGTAGTTTCAAGAGTTTTGGGGTTAATTTTTATAATTTATATAATA
AAAAGTTTTGGTAACGATAAAATTATATTTTAA
>B_platzeri_sample18A

----------TATTATATTTTATTTTTGGTTTTTGATCTGGTATAGTTGGTACTAGATTA
TCACTTATTATTCGTTTAGAATTAGCTAAACCAGGATTATTACTTAATAATGGTCAACTA
TACAATAGAATTATTACTGCTCATGCTTTATTAATAATTTTTTTTATAGTTATACCTTCT
ATAGTAGGAGGTTTTGGTAATTGAATAGTACCTTTAATATTAGGTTCTCCTGATATGAGG
TTTCCACGTTTAAATAATTTAAGGTTTTGATTATTACCAACAGCATTAATATTAATTTTA
GATGCTTGTTTTGTTGATATAGGAGCTGGTACTAGATGAACAGTTTACCCTCCTTTAAGA
ACGTTAGGTCACCCAGGTAGAAGTGTTGATTTAGCAATTTTTAGTTTACATTGTGCTGGT
GTAAGATCTATTTTAGGTGGTATTAATTTTGTATGTACTACTAAAAATTTACGTAGAAGT
TCTATTTCTTTAGAACATATGGGTTTATTTGTATGAACTATTTTTGTTACAGTATTTTTG
TTAGTTTTATCTTTACCAGTATTAGCAGGAGCTATTACTATATTATTAACAGATCGTAAT
ATTAATACTTCATTTTTTGATCCTAGAATAGGGGGTAATCCTTTAATTTATCAACATTTA
TTTTGATTTTTTGGACACCCAGAAGTATATATTTTAATTTTACCAGCATTTGGTATTATT
AGTCATAGAACTTTATATTTAACTGGTAAAAAAGAAGTATTTGGTTCTTTGGGTATAGTT
TATGCTATTTTAAGAATTGGTTTAATTGGGTGTGTAGTTTGAGCTCATCATATATATACT
GTTGGGATAGATTTAGACTCTCGTGCTTATTTTACTGCTGCAACTATAGTAATTGCTGTT
CCTACAGGAGTAAAAGTTTTTAGTTGATTAGCTACTTTATTTGGTTCTGTTATAATTTAT
CAACCTCTTTTATTGTGAGTTTTAGGTTTTATTTTTTTATTTACTATTGGTGGGTTAACT
GGAGTAGTTTTATCTAATTCTAGGTTAGATATTATTTTACATGATACTTACTATGTTGTT
AGTCATTTTCATTATGTTTTAAGTTTAGGTGCTGTGTTTGGTATTTTTTACTGGTGTAACT
TTATGATGAACTTTTATTTTAGGCTTAGTTTATGATAAAGTTATTTTTAGAGCTGTATTT
ATCTTAATATTTATTGGTGTTAATTTAACTTTTTTTCCTCTTCATTTTGCTGGTTTACAG
GGCTACCCTCGTAAATATACTGATTACCCAGATATTTATTCTATTTGAAATATTATTTCT
TCTTTTGGTAGAATATTAAGGGTATTCTCTTTATTATTATTTATTTATTTAATGATTGAT
TCTTTTATTAATTTCCGTGTTTATTTGACTGATTACAATTTAAATTATAGACCTGAAAAT
TCTCTTTCTGGTTATGTTTTTTGTCATAGTTATCAATCTGATATGTTTTTTATTGTTAAA
TAA---
----TAGATTGGTTTCACAGATTTAATTGTAGTTTACTTTTAGGAGTTTTAATTTTTGTT
AGATTATTATTTTTATTCTTGATTTTTAATTCTTTTTATTTTAAAAGTAAAAAGATTGAA
TATCAAGTAGGTGAACTTCTTTGTAGTTTATTTCCTACAATTATTTTAATTTTTCAAATA
ATTCCTTCTTTAAGATTATTATATTATTATGGATTAATAAATATTGATTCTAATTTAACT
ATTAAAGTTATTGGTCATCAATGATATTGAAGATACGATTATAGTGATTTTGAAGATTTA
GAATTTGATTCTTATATAAAATCTATTGATTCATTGGAGGTGGGAGATCTTCGTTTATTA
GATGTAGATAATCGTTGTGTAGTACCTGTAGATTTAAATATTCGTTTTTGTATTACTTCT
GCTGATGTAATTCATGCTTGGGCTTTGTCTAGTTTATCAGTAAAATTAGACGCTATAAGA
GGAATTTTAAGGATTTTAAATTATAATTTTCCTATAGTGGGATTATTTTATGGTCAATGT
TCAGAAATTTGTGGTGCAAACCATAGTTTTATACCTATTGTTGTTGAAGTAACTTTATTT
GATTTATTTAAATCTTGATGTTTATTAAATTAG---------------------------

-------------------TAGATGGTTTTAAATTTGGGGTTATTTTATTTATCTTTAGA
GAATTTATGTTTTTTTTTGGTATTTTTTGAACATTTTTTGATGCTTCTTTAGTTCCTAAT
CATGATTTGGGAGAGATGTGAAGACCTTTTGGTTTATCTTTAGTCAATCCTTTTGGTGTA
CCTTTATTAAATACAATTATTTTATTAAGAAGTGGTGTGACAGTTACATGAGCCCACTAT
AGTTTACTAAGTAATAAAGATAGTTTTAATAGTTTATTAATAACTTGTTTTTTAGCTTTG
TACTTTATATTAATTCAATTAATAGAGTACAAAGAAGCAGGATTCTCAATTTCTGATGGA
GTTTATGGTAGAATTTTTTATTTATCAACTGGTTTTCATGGATTTCATGTTTTTTGTGGT
GGGTTATTTTTATTGTTTAATTTGTATCGTTTAAAACTTTCTCATTTTACATTTAATCAC
CACTTAGGTTTAGAATTTGGTATTATTTACTGACATTTTGTAGATGTAGTTTGATTATTT
TTATTTGTTTTTGTTTATTGATG-------------------------------------

-------------------------------TAATTGAATTTACATTAATTTTAGCTCTA
GTTTCATGATTAAGAACTTTTCTTTGTTTTATTTCTAGAGAAAAAGTATCAATTTATTTT
AGAAAAGAGGGTGATGGTTATTTTAAAACTTTGTCAATGTTAATTGTTGAGATTGTAAGA
GAGTTTTCTCGTCCTATTGCTTTAACTGTTCGTTTAACAGTAAATATTATAGTTGGTCAT
ATAATTAGAATGTCAATTTTTATGATAGTTGAA---AGTTTAGGTTACAAATATTTTTTT
TTCACTATTTTTGCTATTTTAATAGAATGTTTTGTTTTTATTATTCAAAGTTATATTTTT
TCTCGTTTAATTTTTCTTTATTTAAATGAGTAA-------------TAAATTTTGTTAAA
GGATTAGTAATTAATTTACCAGCTAGAAAAAGTTTAAGTTTAAATTGAAATTTTGGAAGT
CAACTAGGTATGATTTTAGGATTTCAAATTATTACTGGAACTTTTTTAGTATTTTATTAT
TCTCCAGATAGGAGATTGGCTTTTAATAGAGTTCAGTATATTATAACTGAAGTAAATTTT
GGATGAATTTTTCGTATTTTTCATTTTAATGGAGCAAGATTATTTTTTATTTTTCTTTAT
TTACATTTTTTTAAAGGATTATTTTTTAGTAGTTACCGTTTAAAAAAAGTTTGAGGGAGT
GGTTTACTTTTATTTTTGTTAATTATGATAGAAGCTTTTATAGGCTATGTTTTAGTGTGG
GCTCAAATAAGATTTTGGGCATCAGTTGTTATTACTAGACTTTTAAGGGTTATTCCTATT
TGAGGTATAAAAATTGTTGTTTGAATTTGAAGTGGGTTTGGTGTTTCAGGAGCAACTTTA
AAATTTTTTTTTGTTTTACATTTTTTATTACCATGAATATTTTTAATTTTAGTTATTGTT
CATTTAATTTTTTTACATGATACTGGGAGAACATCTAAACTTTATTGTCATGGTGATTTT
GATAAAATTAATTTTTATACTTTTTATTGATGAAAAGATGGTTATAATGTTATTATTTGA
TTATTATTTTTTATTTTTAGTTTTCTTTACCCTTTTATTTTAGGAGACCCAGAAATATTT
ATTGAAGCAGATCCTATAATAAGTCCTGTTCATATTATTCCTGAGTGATATTTCTTATTT
GCGTATGCTATTTTACGTGCAATCCCTAACAAAATTTTAGGTGTATTGTTTTTACTTTTA
AGTATTCTAATTTTTTATTTATTTTTATTAAAATTAAATTATTTAAGGTTATTTAAAAAA
AATAATTTATTTTTAGTAAATTTATTTATTTTTTTAAGATTAATTTTAAGATGGTTAGGT
CAATGTTTAGTTGAACCTCCATTTTTATTCTTAAGTGGTTTAGTTTCTTTTTTATATTTT
TTCTTAATTATTTTAATTATATTAAATTATAATTTAAGATCATTAATTTTTAAATATATG
TTTATTATAAATTA--------------------TAATTATTATAGAAGTTTTAATAATT
CTTTTTATTGTTCTTAGAATTGCTTTTGTTACTTTATTTGAACGTCATTTATTAGGGTTA
AGTCAAAATCGTTTAGGGCCTAATAAAGTTTTTTTTATAGGAGTTGTACAAGCAGCATTA
GATGGTGTTAAATTAATATCTAAAGAACAAATTTTACCTATATATTCATCAGATTTATAT
TTTTTATTTGTTCCTGGTATAACTTTTATTTTTATATTTTTTGAATGATTAAGTTTACCA
TTTTTTTTTTATTTTTTAAACTTTCAGTTTTCTTTTTTATTTTTAATAAGTCTAATTGGG
TTTTCAGTTTATTTTACTATTATTAGAGGTTTATTAAGTAATTCTAAATATTCTTTTATT
GGTTCTATTCGTTCTAGAAGTCAAAGAGTTTCTTTCGAAATTGGTTTTTCTATTATAATT
TTAATTTTTATATTTTTTTTTAAATATTTTAAATTTAAAAAATTTGTTTAATTTTAGTTTA
TTTTTTCTTTTTTATTTTTTTATTCTTATAGTTTTAGCAGAGTTGAATCGTGCTCCTTTT
GATTTTGCTGAGGGAGAAAGAGAATTAGTAAGAGGTTTTAATACGGAATATTCAAGGGTG
GCATTTATTTTTTTATTTTTAGGGGAGTATGGAGTACTAATTTTTTTTTAGTGTTTTACTA
AGTGTTATATTTTTTAATTTTTCTTTTATTTTTGTTTTTATTTTTCTTACTTTAATATTG
TTGATTCGTAGAGCGTACCCACGTTATCGTTATGATAAATTAATAAATTTTTTTTGAAAA
TTTGTTTTACCTTTAATTATTATATTTTTTTATATTTTTTTTTTTTATTACTTTT------
----------TATTATTTTTTTTTATTTATTTTATAATGTTTTTTTTATGTATAATTTTT
AATAATTATATTTTTTTGATGAAGAATTTTTCTTTTAATAAGATTGAGATTTTTTTTTTA
AATAAATTTTATTTGAATGATTACAGGAATTTATTTTATTTTATTATTCAAGAAAGTCTA
GGTCTTTTTTTTCTTTTTTTTATTTTTTTAAATTTTCAATGATTGATTTTAATAATAAAA
ATTGGTGTTTCTCCATTCCTTTTTTGGGGGTTCTTTATTGTAGAAAATTTAATAAGTTAT
AGATTATTTTGATTTCTTACTTTCCAAAAGCTTCCTTTTTTACCTTTAGTAAAAGATTT-
--TTTAAATATATATTTTTTTTTTTTTTTTTTTTGGTTTAATATTAATTTATTTAATATTA
ATTAATTTAAAAAAAGTAAATTTTTTAATTTTTTTAAATTCTTCAGAATCTTTTAGATGA
GTTTTAATTAATTTAATTTTTAGAAATTTTAATTTTTTATTTTTATTTTTTTTTTTATTTA
GTTGTGTTTTTTTGTTTTATTTTTTTTTTTAAGTGGTAATTCA---AATAATTTAGATTTA
AATTTAATTTTGTTTATTTTGAATTTTCCTCTTTTTTTAAGATTTTTTTTAAAATTTTTT
AGATTAAATTTTATTTTAAATTTAAATTTTTTTTATTTATTTAATTTTGTTAATTATAGTA
GTTAGTCTTTTTTCTTTTATCTTTTATTTTTTTTATTTTTTTTTTTTATTTTTTTTTTATAA---
----
----------TATTTATTATTATTATGTTTTTTTTTTATTAGTCTTATTTTTGTTGTTTTA
TTTTATTTAATTAATGTAGTTTTATCTTTTAAATTTTTAAATTTTAATAAAATTACTTCT
TTTGAGAGGGGATTTTTAAATGTTGGTAAAATATTTAAATCTTTGAGTTTACATTTTTTT

ATTATTATAATTATGTTTGTTATTTTTGATTTAGAGGTTATTTTTATTTTAGGTTTATTA
GTAAGAGACATAGAAAATATTTTTATTTTTTTATTAATTTTTTTTTTTATGTTATTTAGT
CTTTATTTAGAATGATTTTTAAATAAATTATTATGAATTTTATAA---------------

-------------------------TAAGATTATTAATTTTAATGTTAATTTTTTTAGTA
GAATTAAATTTTAATTTAAAAATTTTAAGGTTTTTTTTAATTTTTGTTAGGTTAATATTT
TTTTTTAGAAGAAGGATTTTTTTTGTTTATTTATTTTTTGAGTTATCTTTATTTCCTATT
TTATTAATAATTTTAGGATACGGTTATCAAATTGAAAAAATTAACTCTTCTTACTATTTA
TTTTTTTTTACTTTAGTTTGTTCTATACCATTTTTTTTTTTTTATTTTTAATTTGGATTTA
AATAAAAATTTTATTTTTTTTGATTATTTTTTTTCTTGAGAATTAGAATTTGTTATAACT
TTAATATTTATACTAAAATTTCCTATTTATTTTTTACATTTTTGACTTCCTAAAGCTCAT
GTAGAATCCCCTACAACTGGTAGAATATTATTAGCTGGTTTATTATTAAAATTAGGGACT
GGAGGTTTTATACGTTTAATAAATTCTTTAAATTTTCATTTTTTAGGGATTTACTATATT
ATTGCTTTTATTGGTATAATTTTAAGTAATTTTAATTGTATTATGCAAAGTGATTTAAAA
TCTTTAGCTGCTTTTTCTTCTATTAATCATATAAGTTTTGTTCTTCTTTCTTTAATTTTT
TTAAATTCTTTTAGTTTCTTAAGAAGTGTTATTATTATATTTTCTCATGGTTTAATTTCA
GTTTTAATATTTTTTTTTTATTGGGGAGTTTTATCATTTTTCTAATAATCGTTTAATTTAT
TATTATTCTAGAATTTTTCTTAACTCTTTATTTTTATGTTTAATTATTAGATTAACTTGA
CTTTATAATAGGGGGGTACCTTTTTCTTTAACATTTTTTAGAGAATTTGTAAGATTCTTA
ATAATTTTTAATGTTAACAATTATTTCTTTTTTTTTAGGGTTTCTATATTTTTTTGTTTCT
TTTTATTATTGTTTATTTTACATTTCTTTAAATTTTTTAGGTAAAATACAATTAAACATT
AATAATAATATTTTAAATTTTGTTAGATTTTTTATTATTTGTAATTCTAACATTTTTTTT
TTCTTATTTATCTTGTAA--

----TAAATTTTTATTTAAAAGGAGAGACTTACTTAAATTATTTTTTTATAGTTTTAATA
ATTTTTATTTTAAGAATGGTATTTTTAAATTTTAGTAGTAATTATTTTACTATAATTTTA
AGTTGAGATCTTTTAGGAATTTCCAGTTATTTTTTAGTTTTATTTTATAATAATTGAGAT
AGAAATAATGGTTCTATAAATGTTTCTTTAACTAATCGTTTGGGGGATTATTTTATATTT
TTTTTTTTTTCTTTTTTTTTATTTTTTTTAATTATTTAATAAATTTTTTAACTTTTATGAAT
TGAATACTTGTTTTAATTTTAATTTTTATAGGTTTTACTAAAAGTGCTCAGTTTCCTTTT
AGTAGGTGATTACCTAAGGCTATGAGTGCTCCTACTCCTGTTAGAGCGTTAGTTCATAGA
AGAACTTTGGTAACTGCTGGGTTAGTATTATTTATAAATTTTTTAAATTTATTAAATTTT
AGTTTATTTTTAAATATTTTAGTTTATATTGGTTTATTAACAATATTTTTTTCTAGGTTA
ATATCTTTTTTTGAAGAAGATTTAAAAAAAGTTGTAGCTTTAAGAACATTATCTCAAATA
GGTTTTAGAGTTTTTATTTTAGGTTTAGGATTAAATTATTTTAGTTTAATACATCTTATT
AGACATGCACTTTTTTAAAAGTTGTTTATTTATTCAAGTTGGAATTTTAATTTATTATTCA
TTTGGTCAACAAGATGGTCGTTTTTATAGTAATTTAAATTATTTTTTTAGTTTTATTCAT
TGACAAATTTTTTTAACTTTATTTTGTCTTTGTGGATTATTTTTTAGTAGAGGGTTAATT
AGAAAAGATATAATTTTAGAATTTTTTTTTTTTTAATAATTATTCTTTATTAATAGTAATA
ATATTTTTTATTGTTATTTTTATAACTTTTTTTTTATTCTTATCGTCTTTTTTTAGGATTA
ATAAAGTTTAATTTTTCGATTTTCTATTTAAGTTTAATAAATTATAAACTTTTTATATCT
TTTTTTTTATATATTTTTTCTTTATTTGGTTTAATAATTATTTCTAATAATTTTGTTATT
TTTCCGTCTTTAATTCTATTTATAGATTTTTTTAGACCTTTATTTTATTTAATTTTATTT
GTTTTTTTAACTTTTTATTATTTAGTAAAAAAAAAAAGTTTTTTAAAATATAGTTTTATA
GTTGATTATTTGGCTAAAATATTTTCTTTTTTTTATAAAAAAATTTAAAATTTGTAGATTTA
TTAATTAATAAAAAATTTAATAATTATATTTTTTATTAAATTTTAAGATAAATTTTAATTTA
TTAAAGTTAAATTTTAATTTTTTTTTTTTTTATAGTTTTAGTATTGTATTTATTAATAGTG
TTTTAA--
----------TGTTATTTTTTTATACTTTACTTATTTTAATTTTATGTTTATTTAGATTT
TTTTCTTTTGATCCTTTTAAAAGATGTCTACTGATAATTTTAAATTTGTTAATTTTGTCT
TTTTATTTAAGTTTAGGTAATAATATTTGGTACAGGTATTATATTAGTTTATTATTTTTA
AGGGGAATTTTTGTTATTCTTGTGTATTTTTCAAGTTTAAGAAGATTTTATTTTTTTTTTT
TTTAATAAACTATATATTTTTTTATTATTGTTGTTAATTATTTTTGTTAATTTTCAATTT
AATTTTTTTTTTTTAAAAATTAGTTTTTTTTTTTTATAA---------------------

-------TAGGTTTATTTATTTATTTTTCTATAAATTTAATAAGATTTTTATATTTTTAT
TTTTTAGTTTTCAGGGTTATTTCTAGAGTTTTAGGGATAGTAATAATAATTTATTTAGTA
AAAAATTTTGGGTCCGATAAAATTTTATTTTAA
>B_sexdentati_sample22
-------------------------------TAAAATGATTTGAAAGTTCAAATCATAAA
GATATTGGTATATTATATTTTATTTTTGGTTTTTGATCTGGTATATTAGGTACAAGTTTA
TCAATAATTATTCGTTTTGAATTAGCAAAACCTGGATTTTTTTTAAATAATGGTCAATTA
TATAATAGTATTATTACAGCACATGCTTTATTAATAATTTTTTTTATAGTAATACCTTCT
ATAGTAGGTGGTTTTGGTAATTGATTATTACCATTGATATTAGGTTCACCAGATATAAGT
TTTCCACGTTTAAATAATTTAAGATTTTGACTTTTACCAACTTCTTTATTTTTACTTATT

GATGCATGTTTTGTTGATATAGGGGCTGGTACAAGATGAACAGTTTATCCTCCATTAAGA
ACTTTGGGGCACCCAGGGAGTAGAGTAGATTTAGCAATTTTTAGTTTACATTGTGCTGGT
GTAAGTTCAATTTTAGGTGGTATTAATTTTATATGTACTACAAAAAATTTACGTAGGAGT
TCTATTTCTTTAGAACACATAAGTTTATTTGTATGAACAATTTTTGTTACTGTTTTTCTT
TTAGTTTTATCATTACCAGTTTTAGCCGGGGCTATTACTATACTTTTAACAGATCGTAAT
ATTAATACTTCTTTTTTTGATCCTAGAATAGGGGGTAATCCTTTAATTTATCAACATTTA
TTTTGATTTTTTGGGCACCCAGAAGTATATATTTTAATTTTACCAGCTTTTGGTATTGTA
AGTCAAAGTACTTTATATTTAACTGGTAAAAAAGAAGTTTTTGGTACTTTAGGTATGGTA
TATGCAATTTTAAGTATTGGTTTAATTGGGTGTGTAGTGTGGGCTCATCATATGTATACT
GTAGGGATAGATTTAGATTCACGTGCTTATTTTACTGCTGCTACAATGGTAATTGCAGTC
CCTACAGGTGTAAAAGTTTTTAGTTGATTAGCAACTTTATTTGGTTCAGTATTAAAATTC
CAACCTTTATTATTATGAGTTTTAGGTTTTATTTTTTTATTTACTATTGGGGGTTTAACA
GGGGTAGTACTTTCAAATTCTAGTTTGGATATTATTTTACATGATACTTATTATGTTGTT
AGTCATTTTCATTATGTTTTAAGTCTAGGGGCAGTATTTGGTATTTTTACTGGGGTAACT
TTATGATGAACTTTAATTATAGGTTTACTTTATGATAAAGTTTTAATAAGCTCAGTTTTT
ATTTTAATATTTATTGGTGTAAATTTAACTTTTTTTCCTTTACATTTTGCTGGTTTACAT
GGTTACCCACGTAAATATATTGATTACCCTGATATTTACTCTTTTTGAAATATTCTTTCT
TCTTTTGGTAGAATACTAAGTGTTTTTTCATTATTTTTATTTATTTTTTTTTTTAATTGAT
TCTTATAATAGTTTTCGTTTATTTTTAGTAGATTTAACTTTAAATTATAGTTCAGAAAAT
TCTTTTTCTAGTTATATTTTTAATCATAGTTATCAATCTGAGATGTTTTTTATTGTAAAA
TA--
---ATAGATTGATTTCATAGTTTTAATTGTAGTTTAATAATAGGTGTATTAATTTTTGTA
AGTTTATTATTTTTATTTTTAATTTTTAATTTAAATTTTTTTAAAAGTAAAAAAATTGAG
TATCAGTTTGGTGAATTATTATGTAGTTTATTTCCTACTATTATTTTATTATTACAAATA
ATTCCTTCTCTTAGTTTATTGTATTTTTATGGTCTAATAAATATTAATTCTAATTTAACT
ATTAAGGTAATTGGGCACCAATGATATTGAAGTTATGATTATAGAGATTTTGAAGATTTG
GAATTTGATTCTTATATAAAATCTTTAGATCTTTTAGAACTAGGGGATAGTCGTTTATTA
GATGTTGATAATCGTTGTGTACTTCCTTTTAATACTAATATTCGTTTTTGTATTACATCT
GCGGATGTTATTCATGCATGAGCTATTTCTAGATTATCAATTAAATTAGATGCTATAAGT
GGTATTTTAAGAATTTTAAATTATAATTTTCCTTCTGTTGGTTTATTTTATGGTCAATGT
TCAGAGATTTGTGGGGCCAATCATAGATTTATACCTATTGTTTTAGAAGTAACTTTATTT
AATTTATTTAATAATTGATGT---------------------------------------

------------------TTTTTATGATCTAAAGATATTGTATTTGAGGGGTTAAGGGGT
TACCATAATTTTTTTGTTATAGATGGTTTTAAATTTGGTGTTATATTATTTATTTTTAGA
GAATTTATATTTTTTTTTGGTATTTTTTGAACTTTTTTTGATGCATCTTTAGTTCCTAAT
CATGATTTAGGTGAAATATGAAGACCTTATGGTTTAGTATTAGTTAATCCTTTTGGAGTA
CCCTTATTAAATACAATTATTTTATTAAGAAGAGGGGTAACTGTAACTTGGGCTCATTAT
AATTTATTAAGAAATAAAGATAGTATAGTAAGTTTGTTAATTACTATTATTTTAGCTTTA
TATTTTATGTTAATTCAATTAATAGAATATAAAGAAGCAAGATTTTCTATTTCAGATGGT
GTATATGGTAGTTTATTTTTTTTATCTACCGGTTTTCATGGTTTTCATGTATTTTGTGGA
GGATTATTTTTATTATTTAATTTTTATCGTTTATTATTATCACATTTTACTTTTAATCAT
CATTTAGGTTTAGAATTTGCTATTATTTATTGACATTTTGTAGATGTTGTATGATTATTT
TTATTTGTTTTTGTTTATTGATGA------------------------------------

-------------------------------TAGTAGAATTTACTTTAACTTTAGCTTTA
ATTTCTTGATTAAGAACTTTTCTAAGTTTTATTTCAAGAGAGAAAGTATCAATTTATTTT
AGGAAAGGAGGTGATACTTTTTTAAAAACGTTAAGTATGTTATTAGTAGAATTAGTAAGT
GAATTTTCTCGTCCTATTGCTCTTACTGTACGTTTGACTGTCAATATCATAGTTGGTCAT
ATAATCAGAATATCAATTTTTCTTCTTTTAGAG---AGAAGTGGAAATAAATATTTTTTC
TTTTTTATTTTTGCTATTTTAATAGAGTGTTTTGTTTTTATTATTCAAAGTTATATTTTT
TCACGTTTAATTTTTTTATATTTAAATGAATA----------------------------

--AAGTTTGAATAAGA
GGTTTAGTAATTTTTTTATTTATTATAATAGAAGCTTTTATAGGTTATGTTTTAGTTTGA
GCACAAATGAGGTTTTGAGCTTCAGTTGTTATTACAAGTTTATTAAGAGTAATTCCTATT
TGAGGTCCTATAATTGTTTCTTGAATTTGAAGTGGGTTTAGTGTTTCTGGTGCTACAATA
AAATTTTTTTTTGTTTTACATTTTCTTTTACCATGATTTTTTTTGGTGTTGATTTTGGTG
CATTTAATTTTTTTACATGATTATGGAAGAACATCAAAAATTTTTTGTCATGGAGATTAT
GATAAAATTAATTTTTATTCTTTTTATTGATGAAAAGATGGTTATAATATAGTTTTATGG
TTATTTTTTTTTATTTTTAGTTTATTAAAACCATTTGTTTTAGGTGACCCAGAAATATTT
ATTGAGGCTGATCCTATAATAAGACCAGTCCATATTATTCCAGAGTGATATTTTTTTATTT
GCATATGCTATTTTACGAGCAATTCCAAATAAAATTTTAGGTGTTTTATTTTTACTTTTG
AGTATTTTAGTTTTTTTTATT---------------------------------------

```
------------------------------------------------------------
------------------------------------------------------------
----------------------------------TATTTATTTTGAGGTTATTGATAATT
TTGTTTATTATTTTTAGGTATTGCTTTTGTTACATTATTTGAACGTCATATTTTAGGTTTA
AGTCAAAATCGTTTAGGACCAAATAAAGTTTGATTTTTAGGTTTTATTCAAGCTGCTTTA
GATGGAGTGAAGTTAATAAGAAAAGAACAAATTTTACCACTTAAGTCTTCGGATCTTTAT
TTTTTAGTAATCCCTAGTGTATCTTTTTTATTTATATTTTTGGAATGATTAAGTTTACCA
TTTATTTTTTTTTTCTTGAATTTTCAGTTTTCATTTTTATTTTTAATATGTTTGGTTGGT
TTTTCAGTTTATTTTACGATTATTAGTGGTTTATTAAGAAATTCTAAATATTCTTTTTTG
GGGGCTATTCGTTCTAGAAGACAAAGTGTTTCTTTTGAAATTGTTTTTTCTATTTATATT
TTTATTTTTATAATTTTTTTAAATTCTTTGAGTTTTTATAGTAATTTTAATTTTTGTTTA
TTAATTTTATTTTATCCTTTTATTTTAATAATTTTGTCTGAACTTAATCGGGCACCATTT
GATTTTTCTGAAAGAGAAAGAGAATTAGTCAGTGGTTTTAATACTGAACATTCTAGAGTA
AGATTTATTTTTCTTTTTTTAGGAGAATATGGAGTTTTAATTTTTTTTAGTGTTTTAAGA
AGTTGTATATTTTTTTAATTTTAATATTTTAATAATTTTTTTTTATCTTTGTTTATTATTG
TTTATTCGTAGAGTTTATCCTCGTTATCGTTATGATTTTTTGATGGATTTATTTTGAAAA
AAGATTCTTCCTGTTTCAATTTTATTTTTATTTTTTTTTTTTTTGGAAATTTAT-------
------------------------------------------------------------
------------------------------------------------------------
------------------------------------TATTTCCTTATTCAAGAAAGTTTA
GGGTTAATATTTTTTAGTTTTTATTAATTTAAATTTTCAATGGTTAATCTTAATATTTAAG
ATTGGAATTTCACCTTTTATTTTTTGATTATTTTATTTTATTGAAAATTTAAGTAGTTTT
ATAATTTTTTGATTTTTAACTTTTCAGAAAATACCATTCATCCCAATTTTAATTAATTA-
--TTTAAATAATTTTTTTTTTTTTTTTTTTTTTTAGGTTTATTTTTAGTTTATATTTTAATT
TTTATTATTAAAAATAGAAAATTTTTAATTTTTTTAAATTCTGTAGAATCTTTTAATTGG
GTTTTAGTT---------------------------------------------------
------------------------------------------------------------
------------------------------------------------------------
------------------------------------------------------------
------------------------------------------------------------
------------------------------------------------------------
------------------------------------------------------------
------------------------------------------------------------
-------------------------------TATTTAAATCTTTTAGTATTCATTTTTTT
ATTATTATAATTATATTTGTTATTTTTGATTTAGAAATTATTTTTATTATTGGTTTTTTA
GTAGGGGATAGAGAATTAGTTTATTTGTTTTTTGTAATTTTTTTTTTTTATGATTTTAAGT
TTATATTATGAGTGATTTTTGGGCAAATTATTATGGGTTTTTTAG---------------
------------------------------------------------------------
--------------------------------TATAGGACTAATATTTTTTAATACAAGA
ATTTTTTTTTTTTTTATTAATTTTTATAAGTTTATTAATTTTAGGTTTAATTTTTTTTAGT
GAAAAATTATTTATATTAAAATTTTTGAGTTTTTTATTAGTTTTAATTTGTATTTTATTT
TTTTTAAGGAGTAATATTTTTTTTATTTATTTATTTTTTGAATTTTCTTTATTTCCTACT
TTAGTAATAATTTTAGGTTTTGGTTATCAAATTGAAAAAATTAATTCTTCTTATTATTTG
ATATTTTATACTTTATTTTGTTCTATACCTTTTTTTTTTTTTTATTTTTTAATTTAGATTTA
AATTTTATTTTTTTTTTTTTTAATTTTTTTCTTTCATGAGAAATATTAATTTTAATTTCT
TTAATATTTTTAATAAAATTTCCAGTATTTTTTTTACATTTATGGTTACCAAAAGCACAT
GTAGAAGCTTCAACTAGTGCTAGTATACTATTAGCAGGATTATTATTAAAACTTGGTACT
GGTGGTTTTATTCGAATTTTGAATAGTTTAAATTTTAGATTTTTAATTTTTTTATTTTATT
ATTTCTTTTTTAGGGATAGTACTTAGTAATTTTTACTGTATTTTACAGAGGGATCTTAAA
TCTTTGGCTGCATTTTCTTCTATTAATCATATAAGATTTGTTTTATTTTTTATTAGTATTA
ATAAATAATTTCAGAGTTTGGAGAAGTATGATTATTATAATTTCACATGGTTATATCTCA
ATTCTTATATTTTATTTTATTGGTGAGTTTTATCATATTTTTAATACTCGAATAGTATAT
TTTTATTCTGGATTATTTATTAATTCTTTATTTTATAGGATTTTAGTAACTTTAACTTGA
CTTTATAATAGAGGTATTCCTTTTTCTCTTACTTTTTTTTTCTGAATTTAGAGGGTTTTTA
GTTATATTTAATTTTAATTATTTTTTTTTTTTTTTTTAGGTTTATTTTTAGTTT--------
------------------------------------------------------------
------------------------------------------------------------
------------------------------------------------------------
------------------------------------------------------------
------------------------------------------------------------
------------------------------------------------------------
------------------------------------------------------------
------------------------------------------------------------
------------------------------------------------------------
---------------------------------------------------------GAT
AGTAATATTGGTTCAATAAATGTTTCTCTTACTAATCGTTTAGGAGATTTTTTTATATTT
TTTTATTTTAGTATATTTTTTTTTTATAATTTTAGTTTTAGATTTTCTAATTTTTTTTTT
TTTAGTTTAATTTTTATTTTAGTGATTATAGGTTTTACAAAAAGTGCTCAATTTCCTTTT
AGTAGTTGGTTACCTAAAGCTATAAGTGCCCCTACTCCTGTTAGAGCTTTGGTACATAGA
AGAACTTTGGTTACTGCTGGTTTAATTTTATTTATAAACTTTAATTTATTTTTAATAAAT
TTAAATTTTATTAATTTTATATTATTTTTTGGTTTATTAACT------------------
```


-TGTTATTTTTTATTTTTAAATTTTATCGTTTTATTTTTATTTTATTATCTTTTGAGTTT
TTAATAATAGGTTTATTTTATTTTTTTAGTTTTAGATTAAATAATATATTATTTTTTTTT
TTTTTTAATTTTAGTTTAATTTCAAGTGTTTTAGGAATATTAATAATAATTTTTATAATA
AAAAATTTTGGTCAAGATAAAGTTTATTTTTA-
>B_borealis_sample9
----------------------------------AATGATTTGAAAGTTCAAATCATAAA
GATATTGGAATGTTATATTTTATTTTTGGATTTTGATCAGGTATATTAGGTACTAGTCTA
TCAATAATTATTCGTTTTGAATTAGCTAAACCTGGTTATTTTTTAAATAATGGACAATTA
TATAATAGTATTATTACTGCTCATGCTTTATTAATAATTTTTTTCATGGTTATACCTTCA
ATAGTCGGAGGTTTTGGAAATTGAATATTGCCTTTAATACTTGGTTCTCCTGATATAAGT
TTTCCACGTTTAAATAATTTAAGTTTTTGATTATTACCAACTTCTTTAATGTTATTAATT
GATGCTTGTTTTGTAGATATAGGAAGTGGAACTAGATGAACAGTTTATCCTCCTTTAAGA
ACGTTAGGTCATCCTGGAAGAAGAGTGGATTTAGCAATTTTTAGACTTCATTGTGCTGGG
GTTAGTTCAATTTTAGGTGGAATTAATTTTATATGTACTACAAAAAATTTACGTAGAAGA
TCTATTTCTTTGGAACATATAAGTTTATTTGTGTGAACAATTTTTGTTACAGTCTTTTTG
TTAGTTTTATCTTTACCAGTTTTAGCGGGGGCTATCACTATATTATTAACTGATCGTAAT
ATTAATACTTCTTTTTTTGATCCTAGTATAGGTGGTAACCCTTTAATTTACCAACATTTA
TTTTGATTTTTTGGGCACCCAGAGGTTTATATTTTAATTTTGCCTGCTTTTGGAATTGTA
AGACAAAGAACTTTATATTTGACAGGTAAAAAAGAAGTTTTTGGGACTTTAGGAATAGTA
TATGCTATTTTAAGAATTGGATTAATTGGATGTGTAGTATGAGCTCATCATATATATACT
GTAGGAATAGACTTAGACTCACGTGCTTATTTTACTGCTGCTACTATAGTGATTGCTGTA
CCTACAGGTGTAAAAGTTTTTAGTTGATTAGCCACTTTATTTGGTTCAGTTTTAAAATTT
CAACCTTTATTACTTTGAGTCTTAGGTTTTATTTTTTTGTTTACTATTGGGGGTTTAACT
GGGGTAATTTTATCTAATTCTAGGTTGGATATTATTTTACATGATACTTATTATGTAGTG
AGTCATTTTCATTATGTTTTGAGGTTAGGAGCTGTATTTGGAATTTTTACTGGGGTTACT
CTTTGATGAACTTTAATTATAGGACTTTTATATGATAAAGTTTTAATAAGAGCAGTATTT
ATTTTAATATTTATTGGTGTAAATTTAACTTTTTTTCCTTTACATTTTGCTGGGCTTCAA
GGGTATCCACGTAAGTATATTGATTATCCAGATATTTATTCTTTTTGAAATATTATTTCT
TCTTTTGGTAGAATATTGAGAGTTTTTTCTTTATTTTTATTTATTTTTTTTTTTAATTGAT
TCATATAGAAATTATCG---

--TTTTTGTT
TCTTCATTATATTTTTTTTTTTAGTTTGTAATAATTATTTTTTTAAAAGTAAAAAAATTGAA
TATCAGTTTGGTGAGTTATTATGTAGATTTTTTCCCACTATTATTTTAATTTTTCAAATA
ATTCCTTCTTTAAGATTACTTTATTATTATGGTTTAATAAATATTGACTCAAATTTAACA
GTTAAGGTCATTGGTCACCAATGATATTGAAGTTATGATTATAGAGATTTTGAAAATTTA
GAGTTTGACTCTTATATAAAATCTTTAGATCTTTTAAATTTGGGGGAGCAACGGTTATTA
GATGTCGATAACCGTTGTGTTCTACCTGTAGATCTTAATATTCGTTTTTGTATTTCTTCA
GCTGATGTAATTCATGCTTGGGCTTTATCAAGAATATCAATTAAATTAGATGCAATAAGA
GGTATCTTAAGAATTTTAAACTATAATTTTCCTTTAATTGGTTTATTTTACGGCAATGT
TCAGAAATTTGTGGTGCTAATCATAGTTTTATACCTATTGTAGTTGAAGTAACATTATTT
GATTTATTTAAGAATTG---

-------------------------------------TAAATTTATTTTTTTGTGGGGCT
ATCTTATTTTATGTTTCTTTTTTATGATCAAAAGATATTGTTTTTGAAGGTTTAAGGGGG
TATCATAATTTTTTTGTTATAGATGGTTTTAAGTTTGGGGTGATTTTATTTATTTTTAGT
GAGTTTATATTTTTTTTTGGTATTTTTTGAACTTTTTTTGATGCATCTTTAGTCCCTAAT

```
CATGATTTAGGTGAAATATGAAGGCCTTATGGTTTAGTTTTAGTTAATCCTTTTGGGGTG
CCATTATTAAATACTATCATTTTATTGAGAAGTGGGGTTACTGTTACATGGGCTCATTAT
AATCTTTTAAGTAATAAGGATAGGGTAGTAAGTTTATTTATTACTATTATTTTAGCTCTG
TATTTTATGTTAATTCAGTTTATAGAATATAAAGAGGCTAGGTTTTCAATTTCTGATGGT
ATTTATGGAAGATTATTTTTTTTTATCTACTGGTTTTCATGGTTTTCATGTATTTTGTGGT
GGGCTATTCCTATTAATAAATTTTTATCGTTTGCTAAAATCTCATTTTACTTTTAGTCAT
CATTTGGGTTTAGAATTTGCTATTATTTACTGACATTTTGTTGATGTTGTATGATTATTT
TTATTTGTTTTTGTTTATTGATGGTCTTTTTA----------------------------
------------------------------------------------------------
------------------------------------------------------------
------------------------------------------------------------
-------------------------------TAGTAGAATTTACTTTAGTTTTTTCTTTA
ATTTCTTGAATAAGAACATTTATAAATTTTGTTTCTAGAGAAAAAGTTTCAATTTATTTT
AGAAAAGATGGTGATTCTTATCTTAAAACTTTGAGAATATTATTAGTAGAGGTAGTAAGA
GAATTTTCTCGTCCAATCGCATTAACTGTGCGTTTAACAGTTAATATTATAGTTGGACAT
ATAATTAGAATATCGATTTTTTTATTATTAGAA---AGAAGGGGAAATAAATATTTTTTT
TTTTTTATTTTTGCAATTTTAATAGAATGTTTTGTTTTTGTTATTCAAAGTTATATTTTT
TCACGTTTAATTTTTTTATATTTAAATGAGTA--TAAAAGATTTATTTATTTTTGTGAAA
GGTTTATTAGTGAACTTGCCTTCAAGTAAAAGTTTAAGTTTAAATTGAAATTTTGGTAGA
CAATTGGGTTTAATTTTATTATTCCAGATTTTTACTGGTTTATTTTTAGTGTTTTATTAT
TCTGCAGATAGTTTATTAGCTTTCGATAGAGTTCAATATTTAATAATAGATGTAAACTAT
GGTTGAATAGTACGTATTTTTCATTTTAATGGAGCTAGTATATTTTTTATTTTTTTATAC
TTGCATTTTTTTAAGGGCTTATTTTTTAGTAGATACCGTTTGGTAAAAGTTTGAATAAGG
GGGATTGTAATTTTTTTATGTATTATAATAGAGGCTTTTATAGGCTATGTTTTAGTTTGA
GCTCAAATAAGTTTTTGAGCTTCGGTAGTAATTACAAGTTTATTAAGAGTTATTCCTAAA
TGGGGGCCAATAATTGTCTCATGAATTTGAAGTGGGTTTAGAGTTTCAGGTGCAACAATA
AAGTTTTTTTTTGTTTTACATTTTTTGTTGCCTTGATTATTCTTAGTTTTAATATTAGTT
CATTTAATTTTTTTACATGATTATGGGAGGACTTCTAAAGTTTATTGTCATGGAGATTAT
GATAAAATTAATTTTTATTCTTTTTATTGATGAAAAGATGGGTATAATTTAATTTTGTGA
ATATTTTTTTTTTTGTTTAGTTTTTTAAGTCCGTTTATTTTAGGAGATCCAGAAATGTTT
ATTGAAGCAGACCCTATAATAAGGCCTGTGCATATTATTCCAGAGTGGTATTTTCTATTT
GCTTATGCTATCTTACGTGCTATTCCTAATAAAGTTTTAGGAGTCTTATTTTTATTATTA
AGAATTTTATTTTTTTTTATTTTTATTACAAAAAAGAATTATGTAAGAATTTTAAAAATT
AGGAATACTTTTTTAGTTTTTATTTTTTTTTTTTACTGGATTTTTATTAAGTTGGTTAGGT
CAATGTTTAGTAGAGCCTCCCTTTTTGAATTTGAGTATATTAATCTCAATTTTTTATTTT
TTTTTTATTTTTTTACTTTTTTTAAATTATTTATTAAGTTTAGTAATTTTTTTATTTGTA
TTTATTATAATTAA-----------------------------------------TAATT
TTATTTATTGTGTTGAGTATTGCTTTTGTAACTTTATATGAACGTCATATTTTAGGTCTT
AGTCAAAATCGTTTAGGTCCTAATAAAGTTTGAATATTAGGTGTTGTACAGGCTGCATTA
GATGGGGTTAAATTAATAAGAAAGGAGCAAGTTTTACCTTTAAAGTCTTCTGATTTGTAT
TTCTTAGTAATTCCTGGAGTGTCTTTTTTGTTTATATTTATAGAGTGGTTAAGGTTACCT
TTTATTTTTTATTTTTTAAACTTTCAATTTTCTTTTCTATTTTTAATATGTTTAGTTGGA
TTTTCAGTATATTTTACTATTATTAGTGGCTTATTAAGAAATTCAAAATATTCATTTTTA
GGGGCTATTCGTTCTAGTAGGCAGAGGGTGTCTTTTGAAATTGTTTTTTCTTTGTATATT
TATATTTTTATAATTTTTTTAAGTTCTTTAAGTTTTATATGAAGTTTTAATTTATGTTTA
TTAATTTTATTTTATCCTTTTATTTTAATAATTTTATCAGAGTTGAATCGTGCACCTTTT
GATTTTAGAGAGAGAGAAAGAGAGTTAGTAAGAGGTTTTAACACTGAACATTCAAGAGTT
AGGTTTATTTTTTTATTTTTGGGGGAATATGGAGTTTTAATTTTTTTTTAGTGTTTTGAGA
AGTTGTTTATTTTTTTATTTTAATTTTTTTATTATTTTTTTTTTATCTTAGAATATTATTA
TTTATTCGAAGTGTTTACCCTCGTTATCGTTATGATTTTTTAATAGAATTATTTTGAAAA
AAAATTTTACCTGTGACTATCCTAATAATATTTTTTTTTTTCTTGGAGATTTAT------
------------------------------------------------------------
------------------------------------------------------------
------------------------------------------------------------
------------------------------------------------------------
------------------------------------------------------------
------------------------------------------------------------
------------------------------------------------------------
------------------------------------------------------------
------------------------------------------------------------
------------------------------------------------------------
------------------------------------------------------------
------------------------------------------------------------
------------------------------------------------------------
------------------------------------------------------------
-------------------TTTTTATTTTTATTGTAATTAGTTTGTTTTTTATTTTTATT
TTTTTTTTTGTTAATATTTTTTTATCTTTTAAATTTAAAGAGAAAAATAAAGTTTTTTCT
TTTGAAAGTGGATTTTTTAAGGTTAGGAAAAAATAATAAAATCTTTTAGAATTCATTTTTTT
ATTATTATAATTATATTTGTAATTTTTGATTTAGAAATTATTTTTATTTTAGGTTTTTTA
GTTGCTGATCAAGAATTAGTTTTTTTATTTTTTTTTATTTTTTTTATTTATAATTTTAAGT
TTATATTATGAGTGGTATTTAGGAAAATTATTGTG-------------------------
```


-------------------------TAAGTTTAATAATTTTAGGATTAATTTTTTTAAGT
GAGAAAAATTATATTTTAAAATTTTTGAGTTTGTCTTTAGTATTAATTTGTATTTTATTT
TTTTTAAGAAGTAATATTTTTTTTATTTATTTATTTTTTGAGTTTTCTTTATTTCCTATT
TTAGTTATAATTTTGGGGTTTGGATACCAAATCGAAAAAATTAATTCTTCTTATTATTTA
ATATTTTATACTTTGTTTTGTTCTATACCATTTTTTTTTTTTATTTTTAATTTAAATTTA
GATTTTATTTTTTTTTTTTTTAATTTTTTTTTATCTTGAGAAATATTGATTCTAGTGTCT
TTGATATTTTTAATAAAATTTCCTGTTTATTTTTTACATTTATGACTACCTAAGGCTCAT
GTAGAAGCTTCAACTAGAGCTAGTATATTATTAGCTGGTTTATTACTAAAATTAGGGACA
GGTGGGTTTATCCGTGTTTTAAGTAGGTTAAATTTTAGATTTTTAATATTTTATTTTTTG
GTTTCTTTTTTAGGCATAGTTTTAAGAAATTTTTTTTGTATTTTACAGAGAGATTTAAAG
TCTTTGGCCGCTTTTTCTTCGATTAACCATATAAGATTTGTTCTTTTAATATTAGTATTG
CTGAATAATTTTAGGGTTTGAAGGGGGATAGTAATTATAATTTCTCATGGTTATATTTCA
ATTTTAATGTTTTATTTTATTGGAGAGTATTACCATATTTTTAATACTCGTATAGTTTAT
TTTTATTCAGGTTTATTTTTAAATTCTTTATTTTTTAGTTTGTTAGTGACCTTAACTTGA
CTTTATAATAGAGGTATCCCCTTTTCTTTAACTTTTTTTTCTGAATTTAGTGGTTTTTTA
GTTTTATTCAA---

-------------------TTTTCTTTTTTATTTTTATATTTTTAATGTTTTTTTTTTTT
AATTTTTATTTTTATTTTTTTTTTAATTGATTGAAAATTTTTTTTATTAGTTTAAATTTG
AATTTTAGAAAGTTAATTTTTTTTTTATTACTTTTAGTTATTGTAATGAGAGTTTTTCTT
TTTATTAATTTTTATTTAGAGGGTGAAAATTATATTTTTTATTTTATTTTTGTTTTGTTA
ATTTTTGTTTTAAGAATAATTTTTTTAAACTTTAGTAATAATTATTTTATAATAATATTG
AGATGAGACCTATTAGGTATTTCAAGTTATTTTTTAGTTTTATTTTATAATAATTGAGAT
AGTAATATTGGTTCTATAAATGTTTCTTTGACTAATCGTTTAGGTGATTATTTTATATTT
TT--

>B_ussuriensis_sp_n

--TGATCAATTA
TATAATAGGGTTATTACTGCTCATGCTTTATTAATAATTTTTTTTATAGTTATACCTTCT
ATGGTTGGTGGTTTTGGTAATTGAATGTTACCTTTAATGTTAGGGGCACCAGATATAAGA
TTTCCACGTTTAAATAATTTAAGATTTTGGTTATTACCAGCTTCTTTACTTCTTCTTATT
GATTCTTGTTTTGTTGATACTGGTTGTGGTACTAGTTGAACTGTTTATCCTCCTTTAAGA
ACTTTAGGTCATCCTGGTGGTAGAGTTGATTTAGCTATTTTTAGTTTACATTGTGCTGGA
GCAAGATCTATTTTAGGGGGTATTAATTTTATATGTACTACAAAGAATCTTCGTAGAAGA

TGTATTTCTTTAGAACATTTAAGTTTATTTGTTTGAACTATTTTTGTTACTGTTTTTTTA
TTAGTTTTGTCTTTACCAGTTTTGGCTGGGGCCATTACTATGTTATTAACTGATCGTAAT
ATTAATACTTCTTTTTTTGACCCTAGAATAGGGGGTAACCCTTTAATTTATCAACATTTA
TTTTGATTTTTTGGTCATCCAGAAGTTTATATTTTAATTTTACCAGCTTTTGGTGTAATT
AGGCAAAGAGCTTTATTTTTAACTGGTAAAAAAGAAGTTTTTGGTAATTTAGGTATAGTA
TATGCTATTTTAAGAATTGGTTTAATTGGTTGTGTAGTTTGAGCTCATCATATATATACT
GTAGGTATAGACTTAGATTCTCGTGCTTATTTTACAGCTGCCACTATAGTTATTGCTGTA
CCTACAGGAGTAAAAGTTTTTAGGTGACTAGCCACATTATTTGGTTCTGTGTTTAAATTT
CAACCTCTATTATTATGAGTTTTAGGATTTATTTTTTTATTTACTATTGGTGGTTTAACT
GGGGTTATTTTATCTAATTCTAGATTAGATATTATTCTACATGATACTTATTATGTAGTT
AGACATTTCCATTATGTACTTAGTTTAGGTGCTGTATTTGGAATTTTTACTGGGGTGTCT
CTTTGATGACCTTTAATTTCTGGGTTAGTTTATGATAAAGCTTTATTTTCTAGAGTATTT
TTTTTAATATTTATTGGGGTAAATTTGACTTTTTTCCCTCTTCATTTTGCTGGTTTACAA
GGGTATCCTCGTAAATATATTGATTACCCAGATATTTATAGAATTTGAAATATTATTTCT
TCTTTCGGTAGGATACTTAGTGTATTTTCTTTATTTTTATTTTTTTTTATGTTAATTGAT
TCTTTTAATAATTTTCGTTTACTTATTAATGATTCTTTTACTAATAGAGGGGCAGAAACT
TCTTTTTCTAATTATATTTTTTTCTCATGGTTATCAGAGAGAAATTTTTTTTATTGTTAAA
TAAATTTTAAGATTTTTTCAAGGTGTAAATTTATTATTTTCTGGTAGTTTTTTTTCTTTT
TATATAGATTGRTTCCATAGTTTTAATTGTAGTCTTTTATTGGGTGTTTTAATTTTTGTT
AGATTTTTATTTATTTTTTTGATATTAAATAAATTATTTTTTAAAGGAGAAAAAATAGAA
TATCAATTTGGGGAATTACTTTGTAGTGTTTTCCCAACTTTAATTCTTTTTTTACAAATA
TTCCCATCTTTAGGTCTTTTATATTATTATGGTTTAATAAATTTAGATTCTAATTTAACT
ATTAAGGTAACTGGTCATCAGTGATATTGAAGTTATGATTATAGTGGGTTAGATGGTGTT
GATTTTGACTCATATATAGTAATAACTGATTCTTTAGAATTAGGGGGGTTACGTCTTCTT
GATGTAGATAACCGTTGTATTCTTCCTAATGATGTAAATACACGTTTTTGTGTTACTTCT
GCAGATGTTATTCATGCTTGAGCTTTATCTAGTTTATCTATTAAACTAGATGCTATAAGA
GGGGTTCTTAGAATTTTAAATTATAATTTCCCATTAATTGGTGTTTATTATGGTCAATGT
TCAGAGATTTGTGGGGCTAACCATAGATTTATACCTATTGTTATTGAAGTAATTTTATAT
GATATATTTAAATCTTGATGTAATTTATTTTAAATTATTTACAATTTATTTCATAATTTT
CATATTTTAAGGTTATCAGTTTATCCATATTTAATTTTTTTTGTTTCTTTAAGTCTTACT
TCTTCTTTAGTTGTTTTTCTTAAATTTGGTATTGTTTCAGGTTTTTTTTTGAATTTTTTA
TTCTTAATTTATATTTCTTTTTTATGAGGAAAAGATATTGTTTTTGAAGGTTTAAGAGGT
TTTCATAATTTTTTTGTTATAGATGGTTTTAAATTTGGTGTATTATTATTTATTTTTAGA
GAGTTTATATTTTTTTTTAGAATTTTTTGAACTTTTTTTGACGCAGCTTTAGTACCAGTT
CATGATTTAGGTGAGATATGAAGTCCTTATGGTCTTTCTTTAGTTAACCCTTTTGGGGTC
CCACTATTAAATACTATTATTTTGTTAAGTAGGGGGGTTACTGTTACTTGGTCACATTAT
AGTTTATTAAGAAATAAGGAGAGAAGTGTAAGTTTAATTTTCACTTGTTTGTTGGCTTTT
TATTTTATTTTAGTTCAAGGTATAGAGTATAAAGAGGCTAGATTTTCAATTTCTGATGGA
GTTTATGGAAGTTTATTTTTTCTTTCTACTGGGTTTCATGGTTTTCATGTTCTTTGTGGT
GGTCTTTTTCTTTTAGTTAATTTATATCGTCTTACCCTTTCTCATTTTACTTTTAATCAT
CATTTAGGACTTGAATTTGCTATTATTTATTGACATTTTGTTGATGTAGTCTGATTGTTT
TTGTTTGTGTTTGTTTATTGATGATCTTTTTA-ATTCATACTGTTTATTTTTTAGATATT
TTTATTTTTGTTTTTTTGTACCAGTTTGTTTTTTCATTTAATAAGGGTGGTTTGTTTATA
ATTTTCTCATCTTTAAAATCTAATGTTTTTGAAGCTTTTAGTTTTTCAAAGAATTTATTC
CTTAGAAGTATTATTTCTAGAGGTTTATTTTTTTTTACTTCTTTTTATATGTTTTTTAGGA
TATTTTAGGTATTCTTTTAGACCATGTGGTATAATTGAATTTACTCTATTCTTCTCTTTG
ATTTTTTGATTATCAACATTTTTAAGGTTTATTACTAGAGAAAAACTTTCTATTTATTTT
AGTAAAGGTGGTGATTTTTATCTAAAGACTTTAAGAATATTATTAGTAGAAATTGTTAGT
GAATTTTCTCGTCCAATTGCTTTAACTGTACGTTTAACTGTTAATTTGATGGTTGGTCAC
ATAATTAGCTTTGCTTTACATTTATTTTCTATT---TCTTCTTTCTTTACTAGAGTTTTT
TTCTCTTTAGGGGCTATTCTTTTAGAATGTTTCGTCTTTTTTATTCAAAGTTATATTTTT
TCACGTTTGATTTTTCTTTATTTAAACGAGTATTTGGTTGTTATAATTAATTTTGTTAAA
GGTTTACTTGTTAATTTACCCACTAGAAAGAATTTAAGTTTAAATTGAAATTTTGGTAGA
CAGTTAGCTTTAATTTTAGGGTTTCAATTATTTACTGGTGTTTTCTTAGTTTTTTTTTTT
ACTCCATCTGGGGTTGATGCTTTTAATAGGGTACAATATATTATAACTGAAGTTAATGGT
GGTTATTTATTTCGTATCCTTCATTTTAATGGAGCTAGTTTATTTTTTTATTTTTCTTTAT
TTACATTTTTTTAAAGGTTTATTTTTTTCTAGTTATCGTTTAAAAAAAGTTTGAGGTAGT
GGTTTATTATTATTTTTATTAGTTATAATAGAGGCTTTTATAGGTTATGTTCTAGTTTGG
GCTCAAATAAGTTTTTGAGCTTCTGTAGTAATTACAAGATTATTAAGAGTTATCCCTATT
TATGGTAATAGTTTAGTTTCATGAATTTGAGGTGGTTTTAGAGTTAATAGAGCTACACTT
AAATTTTTTTTTGTTCTTCATTTTCTTTTGCCATGATTCTTTTTAGTTTTGGTACTAATT
CATTTAATTTTACTTCATGATACTGGAAGCACAAATAAAATTTATTGTCATGGGAATGAG
GGAAAATTATCTTTTTATTCTTTTTTTTGATGAAAAGATGGGTATAACATAATTATTTGA
TTAACTTTTTTTATCTTTTGTTTTTTTTATCCATTTAATTTAGGGGACCCAGAAATATTT
ATTGAGTCTGACCCTATATTAAGACCTGTTCATATTATTCCAGAGTGATATTTTTTATTT
GCTTATGCTATTTTACGTGCTATCCCTAATAAAATTTTAGGTGTAATTTCATTATTATTA
AGAATTTTAGTATTTTACTCTTTTATTCTTTTAGATGTTAAAAATTCTCTTTTTAAAAAA
AGTAATAGGGGTTTAGTTTTTATTTTTTTATTTATTAGAATTTTCTTAAGATGATTAGGG
CAATGTCTTGTAGAACCACCATTTTTGTTTTTAAGTGGTTTATTTTCGGTTTTATATTTT
TTTTTTATTTCTCTTATTTTTATAAATTTTTTCTTAAGAAGTTTTCTTTTTAATTTTGTA
TTTATTATAAAAAA-ATATTCTTGATTTTTTTAATAATATTATTTTTGTTAATTTTTATT

CTATTTATTATTTTAGGAGTAGCTTTTTGTACTCTTTTTGAACGTCATGTTTTAGGTCTT
AGACAGAATCGTTTAGGTCCTAATAAACTTAGTTTGGTTGGTGTACTTCAAGCTGGTATT
GATGGGGTTAAACTTATATCTAAAGAACAATTATTACCAGTTTATTCTTCTGATTTATTT
TTTTTTTTTACTCCTGTATTCTTTTTTATTTTTATATTTTTAGAGTGGGGATGTTTACCT
TTTAATTATTTTTTTTTAAATTTTCAATTTTCTTTTTTATTTTTTCTTTGTTTAGTAGGG
GTTTCAGTTTACTTTACTATTATTAGGGGGGTTATTAGTAATTCTAAATATTCTTTTCTT
GGTGCTGTTCGTGCTAGGAGTCAAAGTATTTCTTTTGAAATTGCTTTTTCTATTACAATT
TTTTGTTTAATTTTATGTATTTCTTCTTTTAGTTTTATTTCTTTTTTTTCTATTAAATTA
TTATTACTTTTTCCTTTATTTATTTTGTTAATTATAGCAGAATTAAATCGTGCACCATTT
GATTTTGCTGAGGGTGAGAGTGAATTAGTAAGAGGGTTTAATACTGAACATTCAAGTCTA
AGTTTTGTATTTTTATTTTTAGGTGAGTATGGTGTTTTACTTTTTTTTAGAAGTTTTCTG
TCTATGTTAATATTTAATGATTCAATTTTTTTTTGATTTTTTATGTTTTTTTTAATTTTA
AGTATCCGTAGAGCTTTCCCACGTTTTCGTTATGATCTATTAATAGAACTTTTTTGAAAA
GTTATTCTACCATTATCAATTATTTTTTTTTTTATTTTTCTTTT-----------ATTTTT
CTTTCATTTATTTTATTTTTTTTCTTTTATATAGGAATTTTTATATTCAGTTATTTTGTT
AATAATTATGTTATTTGGTGAGGAGTATTTTTAATTCTTAGATTAAGATTTATTTTAATT
AATAAATTAAATGAGGATTCCCTCCCTAATATTATTTATTTTATTGTTCAAGAAAGATGT
GGTAGTTTTTTTCTTCTTTTTTCTTTTTTTAATATTCAATGATTATTTTTATTAATTAAA
GTAGGGGTTTCACCATTTATTTTTTGAGTTTTTTTTTTTTATTGAAAATTTAAAGATAAGT
GGTTTATTCTGATTTTTGACACTTCAAAAATTTCCTTTTATACCTATTTTAATAATATT-
--TTTTTTTAATTTTATTAAAATTTTTTTTTATTGGTGTTTTGTTAGTTTATTTAATTTTT
TTTATAATTAAATCTTTAAARACATTAATTTGTTTAAATTCTATTGAAAGTTTTAGATGA
TTACTTATACTTTTTTATTTTTTTTTATTTTTCTTGTTTTTATTTATTCATTTTTTATATT
TTAAGTTTTTATTTATTAATTTTTAATGACTCTTTTGAATTATCAATTTTTTTATTAAGA
GTACCTTTAACCCCAAGATTTTTTATTAAATTCTTAAGTTTATCTTTTATAAGAGAGTCT
CTATTAATTTTTTGATTTATTTTTATCTATAATATTATTAAGTCTTTTATCTTTATTTTAC
CAATTTGTATGGTTATTTTTAAAGTTTAAAAATAAAAGTATCATAAATAGATTTTTTTTT
TTTTTTTTT-ATTTTAATTTTCTTTTTTTGTATTATTAA--------------------
---TTTAAAATTTTTATTATTATATTTTTATCTTTTTTAAGTTTAGTATTTGTAATTTTA
TTTATTTTTTTAAATATTCTATTATCTTTTAAATTAAATAATACTTCTAAAATTAGATCT
TTCGAGAGAGGGTTTGAAAGAATCGGTAAATTATTTAAATCTTTTAGATTACATTTTTTT
CTTATTATAGTAATTTTTATTGTTTTTGATTTGGAGATTATTTTACTTTTAGGGGGTGTT
CTTTCTACAAATGAGGGTATTATTTCTTTATTTTTTTTTGGTTATTTTTATACTTGGGAGT
TTATATTTGGAATGATTTCTTAAAAAATTATTTTGATCTTATTGT---------------
ATAGTAATATTATTTATTTTTTTGTTTTTTAATTATTATGGTTGATTTTTATTTATAATT
TTTTTTATTATAAAATTTTTTTTTTCTTTTATTAATTGGGGAGTTGGTCTTTTTAATAAT
AATATAGTATTTTTTTTAGTCTATCTTAGTATAATTATTTTAGGAGTTATTTTAATAACT
GAAAAAAATAAAATTTTATCAATTGGAGGGGGGGTATTAATTTTAGTTTGTATATCATTT
TTTTTTAGTAATAATTTATTTTTTATATATTTATTTTTTGAGTTATCTTTTTTACCAGTA
GTTTTAATAATTTTAGGTTTTGGGTATCAAATTGAAAAAATTAATTCTTCTTTTTTATCTA
TTGTTTTATACAGTTTTTTGTTCTATACCATTTTTTATCTTTATTTTTAATATTGATTCT
TCTTTATGTTATCATTTTATTGATTTTTATTGTAGATGAGAAATAACTTTATTAATTATA
TTACTATTTCTTTTAAAATTCCCTGTTTATTTTCTTCATTTATGACTTCCTAAAGCTCAT
GTAGAGGCTCCTACTGTAGCTAGAATATTGTTGGCTGGACTTTTATTAAAATTAGGTACG
GGAGGAGTTTTACGGTGTTTATTTATTTTAAATTTTACATTTATAGGAGGGTTATTTATT
ATTTCATTTTTAGGTATAATTTTAAGAAATTTTATATGTATTGTCCAAAGTGATTTAAAA
GCATTAGCTGCTTTTTCTTCAATTAATCATATAAGATTTGTCCTTTTATTATTAATTTTA
TTGTCTTCAAGTAGAATTTGAAGGGGGGTTATTATTATATTTTCACATGGGTTAATTTCT
TTATTAATATTTTTTTATATTGGTGAGTTCTATCATTCTTCTAATACTCGTATATTATAT
TATTATTCTGGGGGGTTTATTTTTTCATTATTTAATTGTTTGATAATCAGCACAGTATGA
TTATTTAATGGAGGAGTTCCTTTTTCTCTTTCATTTTTTTTCAGAATTTGTTGGGTTTAGT
AATATTTTTTTATATAATTTATTTTTGTTTTTTTTAGGTAGTATTTATTTTTTTCCTTTCT
TTTTATTATAACCTGTTTTTTGTAATAATTAATTTTACTGGGAAAGAAGGAGTTTTAAAT
AATAGTTTTATGGTTACTCTTGGTGGTTGATTTTTAATTATAAATATTAATATTTTTTATT
TTTTTATTTCTTTTTTTAG---ATGAGGGTATTATTTCTTTATTTTTTTTTGGTTATTTTTA
TACTTGGGAGTTTATATTTGGAATGATTTCTTAAAAAATTATTTTGATCTTATTGTAAAT
TTTTTAATTTTTTTATATTTTTTTTAGTATTTTATGTTTATTCATATTTGTAATATTAAAT
GTTTATAGATTTTTTTTTTTTTTATTGTAGATTGAAAGTTTATGTCTTTAATTTTTAATCTT
ATATTCTGAAAAATTTTATTTCTTTTTTTTTTTTATTGATTGTAGTTTTAAGAATTATTATT
TTTATTAATTTTTATTTAGCTGATGAGTTTTATCTAAATTATTTCCTTTTTGTATTAATT
ATTTTTGTTTTAAGTATAATTTTTTTAAACTTTAGTAGTAATTCTTTAAGAATACTTTTA
AGATGGGATATTTTAGGGATTTCTAGTTGTTTTCTAGTTTTATTTTATAATAATTGGGAT
AGAAGAGTTGGGGCTATAAATGTTTCTCTTACTAATCGTTTAGGTGATTATATAATGTTT
TTTTTCTTTTTTAGAAGATTTTTTTATTGATTTTTTTTTTTCTTCTTTCTCTTTTTTAACT
CCGTTTAGAATTTTATTTTTAATTGCTTGTGGTTTTACTAAGAGAGCTCAATTTCCTTTT
AGAAGATGACTTCCTAAAGCTATAAGAGCTCCTACTCCAGTAAGAGCACTTGTGCATAGT
AGTACTTTAGTTACTGCTGGTTTAATTTTATTTATAAATTTTTTTGATATAGTATTTTTT
TTCAATTCTTTAATTATTCTTTCTATTATTGGTGGTGCTACTATAATATTTTCTTCTATT
ATAGCTCTTTTGGAAGAGGATTTAAAAAAAGTTGTTGCCTTAAGTACTTTATCTCAAATA
GGTTTTTCTGTTTTAGTTTTAGGCTGTGGTTTTTATTATCTTTCTTTTGTTCATTTGGTT
AGTCATGCTTTATTTAAAAGAGCACTTTTTATTCAAATAGGTGGTTTAATTTATTTTTCT

TTTGGGAGTCAAGATTCACGT---GGTCTTTCAGGTGGTGGAGGTTTAATAATTTTAAAA
TTTAATTTTCTTATTACTGTTTTTTGTCTTTGTGGTTTATTATTTTTAGGGGGTATATTA
AGAAAAGATTTAATTTTAGAATTATTTTTAACTTCTAATTATTATTATTTTTTTTTTTTT
TTTTTTATCATTATTGTGTTTTTGACTTTTCTTTATAGATATCGTTTATTTAAAGGGGTT
ACTGCATTGAATAGGAGTTTAAATGTTTGGTATATAAATTTGAGTGGTTGTTTATCATCT
TTTTTTTTAATTATTTTTTCTATTTCTTTTTTATGATTTCTGTCAAAAAATTTTTTTTTT
ATCCCAAGACTTTTTATTAATAATGATGTTCTTACTCCTTTATTATATTTATTTATATTT
ATTCTAGTATTTAAGCTTATTATAATTTTAAATTTAAATTATTTTAAATATAGTTTCACA
GTTGACCTTTTAGCTTTAATATTTTCTTTTTTTATTTTATTTTTAAAAACTTTTGATTTT
TTATTAGTTAAATTAATATTAGGGTATAAAATTTTATCTTTAAAAAACCATTCTTTATTT
CTTTTTAATAATTTACGTGGGGTGGTTTTGTTATTTTTAGTTTTTTTGACTATTTTTTTT
TAG---
---------------ATACTTTTGTGTTTTTTTGTGTTTTTAGCTGGAGGATTAAGATTT
TTATCTTTTGACCCTTTTAAAAGTTGTTTAATATTAGTTTTAAGAGTTTTATCTTTTTCT
TTTTTTTTTAGTATTGAATTAGGTGTTTGGGTAAGTTATTTTATTAGGTTATTATTTTTG
AGGGGTATTTTTGTTATATTAGTTTATTTTTCTAGTTTAAGTAGCTTTTTCTTTTTTCTT
TTTAGTAGTAGAATATTTTTATTATGTGGGATTTTTTTTTTTTTACACCTTTATAAATATT
AATTTGAAATTAGGGGTTTCTTATTATTTTTTAAGGTTTTTTAGTTGAGGTTATTTATTA
ATTTTATGTTTATTATTAATATTTCTTTTATTTTTAAGTTTAATATTAAATTTTCAAGGT
GCTATACGAAAATTTTATATTTTTTATATT----ATTTTATATTTTTTATATTTCTTGGT
TTAATTTTTTTTTTCTTTAAATTTTTTCGTTTAATTTTTATTCTTATATCTTTTGAATTT
TTTTTAATAGCGTTATTTTATTGAGTTTCTTCCTTTGTATCTCCTTATTTATTTTTTTTT
TTTCTTAGGGTTAGAATTCTTTCTAGTGTAACAGGGGTTTCTCTTTTAGTTTTATTTATA
AAAAATTTTGGGAATGATAAAGTTTTATTTTAC
>B_willibaldi_CD3664
ATAAATAATTTTAAATTTCAAAAAGGTTATCATTATTGAATTGAAAGTACTAATCATAAA
GATATTGGAATATTATATTTTATCTTTGGTTTTTTTTCTGGTATAATTGGTACAAGTTTA
TCTTTAATTATTCGTTTAGAATTAGCTAAACCTGAATATTTTTTAAGTAATGGTCAATTA
TATAATAGTATAATTACTGCCCATGCTTTATTAATAATTTTTTTTATAGTTATACCTTCT
ATAGTAGGTGGTTTTGGAAATTGAATATTACCTTTAATATTAGGTTCACCTGATATAAGG
TTTCCACGTTTAAATAATATTAGTTTTTGATTGTTACCTATTTCTTTAATTTTATTATTA
CTTGCTTGTTTTGTAGGTTTAGGTGCTGGTACAAGTTGAACAGTTTATCCTCCTTTAAGT
ACTTTAGGGCATCCTGGAGAAAGTGTTGATTTAGCAATTTTTAGACTTCATTGTGCTGGT
ATTAGATCAATCTTAGGAGGAATTAATTTTATGTGTACAACTAAAAATTTACGTAGAAGT
TCTATTTCTTTAGAACATATAGGTTTATTTGTTTGAACTATTTTTGTTACTGTGTTTTTA
TTAGTTTTATCTTTACCAGTTTTAGCTGGGGCTATTACTATATTATTAACTGATCGTAAT
ATTAATACTTCTTTTTTTGATCCTAGAATGGGTGGTAATCCATTAATTTATCAACATTTA
TTTTGATTTTTTGGTCACCCTGAAGTATATATTTTAATTTTACCAGCTTTCGGAATTATT
AGACATAGAATTCTTTTTATAACTGGTAAAAAAGAAGTATTTGGTTCTTTAGGTATAGTT
TATGCTATTTTAAGTATTGGTTTAATTGGTTGTGTAGTTTGAGCTCATCATATATATACT
GTTGGTATAGATTTAGATTCTCGTGCTTATTTTTCTGCTGCTACTATAGTTATTGCAGTT
CCTACAGGTGTAAAAGTTTTTAGTTGGTTAGCTACTCTTTTTGGTTCAATTTTAATTTTT
CAACCTTTATTATTATGAATCTTAGGATTTATTTTTTTATTTACTATTGGTGGTTTAACA
GGTGTAATTTTATCTAATTCAAGTTTAGATATTATTTTACATGATACTTATTATGTTGTT
AGACATTTTCATTATGTTTTAAGTTTAGGTGCTGTTTTTGGTATTTTTACAGGTGTTGCT
TTATGGTGAACAACTATTATAGGAGTACTTTATAATAAAATTTTATTTAGAGCTACATTT
ATTTTAATATTTATTGGTGTAAATATAACTTTTTTTCCTTTACATTTTGCTGGGTTACAA
GGTTATCCACGTAAATATATAGATTATCCTGATATTTATTCTTTATGAAATATTGTTTCT
TCTTATGGTAGAATATTAAGAGTTTTTTCTTTATTTTTATTTATTTTTCTTTTAATTGAA
TCTTTTATTAGTTTTAAATTATTATTAGTAGATTCTAATACTAATTATAGTCCTGAAAAT
TCTTTATCAAGTTATATTTTTAGTCATAGATACCAATCTGAAATATTTTTTGTTGTTAAA
TAAATTTATAGATTTTTTCAAGGTTATAATTTAATATTTCAAAATAGTTTATTTTCATTT
TTTATAGATTGATTTCATAGATTTAATTATAGTCTTTTATTAGGAGTTTTAATATTTGTT
AGATTTTTATTTATTTTTTTAATTTTTAATATTTTTTTATTTAAAAGTAAAAAAATTGAA
TATCAATTTGGTGAATTATTATGTAGACTTTTTCCTACTTTAATTTTATTATTTCAAATG
ATTCCTTCTCTAAGTTTACTTTATTATTATGGTTTAATAAATATTAATTCTAATTTAACT
ATTAAAGTTATTGGTCATCAATGATATTGAAGTTATGATTACAGTGATTTTGAAAATTTA
GATTTTGATTCTTATATAAAATCTTTAGATTTATTAGATTTAGGTGATAAACGTTTATTA
GATGTTGATAATCATTGTATTTTACCAATTGAAACTAATATCCGTTTTTGTATTACTTCA
GCTGATGTTATTCATGCTTGAGTTTTATCAAGTCTTTCTGTTAAATTAGATGCTATAAGT
GGAATTTTAAGTATTTTAAATTATAATTTTTCTTCTGTTGGTTTATTTTATGGTCAATGT
TCAGAAATTTGTGGTGCTAATCATAGTTTTATACCTATTGTTGTTGAAGTTACTTTATTT
AATTTATTTAAAAATTGATGTTTATTATTTTAA---------------------------
---ATTTTAAGTTTATCAATTTATCCTTTTTTAGTTTTTTTATGTGTTTCAGGTGTAACT
TCTTCTTTAGTAATTTTTTTAAAATATGGTTTAATTTCAGGTTTACTAGTTAGATTTTTA
ATTTTATTTAATGTAATTTTTTTATGATCTAAAGATATTTCTTTTGAAGGTTTAAGTGGT
TATCATAATTTTTATGTTATAGATGGTTTTAAATTTGGTGTAATTTTATTTATTTTTAGA
GAATTCATATTTTTTTTTAGAATTTTTTGAACTTTTTTTGATGTTTCTTTAGTACCTACT
CATGATTTAGGTGAAATATGAAGACCTTATGGTTTAATTTTGGTTAATCCTTTTGGAGTA
CCTTTATTAAATACTATTATTTTATTAAGTAGTGGTGTAACTGTTACTTGAGCTCATTAT
AATTTATTAAGAAATAAAGATAGTTTTATTAGTTTATTATTAACTTGTTTTTTAGCTTTT

TATTTTATTTTAATTCAATTTATAGAATATAAAGAAGCAAGGTTTTCTATTTCTGATGGT
ATTTTTGGAAGTATTTTTTTTTTATCTACAGGTTTTCATGGTTTTCATGTTTTATGTGGT
GGGTTATTTTTAATATTTAATTTTTTTCGACTTTTTATTAATCATTTTAATTTTAATCAT
CATTTAGGTTTAGAATTTGCTATTATTTATTGACATTTTGTAGATGTAGTATGATTATTT
TTATTTGTTTTTGTTTATTGATGATCTTTTTA-ATAAATAATGTTTATTTTTTAGATATT
TTAATTTTTATTTTTATTTTTCAATTTATTTTTTTTTTTAAATTTAATCTTTTATTTATT
TTTTTTAAGAAATTTTTATTTTCTTTAAATGAAATTTTTAGTTATAGTAAAAATTTAATT
TTAAGAAGTTTTATTTCAATTTTTATTTTTTTTATTTTTAATTATTTGTTGTTATGGTGGT
TATTTTTGTTATTCTTTTTGTCCTTGTGGTATAATTGAGTTTACTTTAAGTTTATCTTTA
ATTTCTTGATTAAGAACTTTTTTATGTTTTATTTCAAGAGAAAAATTATTTGTTTATTTT
AGAAAAGAAGGTGATCAATATTTAAAAACTTTAAGAATATTATTAATTGAAATTGTGAGA
GAATTTTCTCGTCCTATTGCTTTAACTGTTCGTTTAACAGTTAATATTTTAGTTGGTCAT
ATAATTAGTATATCTATTTTTTTTATTAGTAGAA---AATTTAGGTTATAAATATTTTTTT
GTTACTATTTTAGCAATTTTAATAGAATGTTTTGTTTTTATTATTCAAAGTTATATTTTT
TCTCGTTTAATTTTTTTATATATTAATGAATAAATAGTTTATTTAAAATTATTTATTAAA
AGTTTAGTTATTAATTTACCTACTAGTAAAAGTTTAAGTTTAAATTGAAATTTTGGTAGA
CAATTAGGTTTAATTTTAGTATTTCAAATTTTAACTGGTACATTTTTAGTTTTTTATTAC
ACTCCAGATAGTTTATTAGCTTTTAATAGTGTTCAATATATTATAAGAGAAGTTAATTTA
GGTTGAATTTTTCGTATTTTTCATTTTAATGGTGCTAGTTTATTTTTTTATTTTCATTTAT
TTACATTTTTTTAAAGGTTTATTTTTTTCTAGTTATCGTTTATTTAAAGTTTGAGGAAGA
GGTTTAATTATTTTTTTATTAGTTATAATAGAAGCTTTTATAGGTTATGTTTTAGTTTGA
GCACAAATAAGATTTTGAGCTTCAGTTGTTATTACTAGTTTATTAAGTGTGATTCCTATT
TGAGGAATAAAAAATTGTTATATGAATTTGAAGAGGATTTGGTGTTTCTGGGGCTACATTA
AAATTTTTTTTTGTATTACATTTTCTTTTACCTTGATTTTTATTAGTAGTAATTTTAATA
CATTTAATTTTTTTACATGATTTTGGTAGAACTTCTAAAGTTTTATGTTTAAGAGATTTT
GATAAAATTAATTTTTTTTTCTTTTTATTGATGAAAGGATGGTTATAATTTATTAATTTGA
TTTTTATTTTTTATTTTTAGTTTTATTTTACCTTTTTTTCTAGGTGACCCAGAAATATTT
ATTGAAGCTGATCCTATAATAAGACCAGTTCATATTGTTCCTGAGTGATATTTTTTATTT
GCCTATGCTATTTTACGTGCTATTCCTAATAAGATTTTAGGTGTAATTTTTTTATTATTA
AGTATTTTAATTTTTTATTTTTTTTTTATTATTTCAAAATTATTTAAGTTTATTTAAGAAA
AATAATATTTTTTTTAGTTAATATTTTTATTTTTGTTAGATTAATTTTAAGTTGGTTAGGT
CAATGTTTAGTTGAAGTTCCATTTTTATTTTTAAGAATTTTATTTTCAATTTTATATTTT
TTTTTTATTTTATTTTTATTATTAAATTATTTTTTAAGATGAGTATTATTTAATTTTGTA
TTTATTATAAATTA-------TTGATTTTAATAATTATTTTTAGAAGTTTATTGATAATT
TTATTCTTAATTTTAGGTATTGCTTTTGTTACTCTTTATGAACGTCATTTATTAGGTTTA
AGACAAAATCGTTTAGGTCCTAATAAAAATTTGTTTTTAGGTGTTATTCAAGCAGCCTTA
GATGGAGTTAAATTGATATCTAAAGAACAAATTTTACCTATTTATTCTTCAGATATTTAT
TTTTTAATTATTCCTGGAGTTTCTTTTATTTTTATAATCTTAGAATGGTTATGTTTACCT
TATATTTATTTTTTTTTTAAATTTTCAATTTTCTTTATTATATATTTTAGTTTTAATTGGG
TTTTCTGTTTATTTTACTATTATTAGTGGTTTTATAAGTAATTCTAAATATTCTTTTATT
GGAGTTATTCGTTCAAGAAGACAAAGAGTTTCTTTTGAAGTTATATTTTCTATTTTTATA
GTAATTTTTATATTATTTTTTTAATTCTTTTAAATTATATTTTTTTTTTTAATTTTAGTTTA
TTTTTTTTCTTTTTTATTTTTTTATTTTTAATTTTAGCTGAATTAAATCGTGCACCTTTT
GATTTTTCTGAAGGTGAAAGTGAATTAGTTAGAGGTTTTAATACTGAACATTCTAGAGTA
AGTTTTATTTTTTTATTTTTAGGTGAGTATGGGATTTTAATTTTTTTTAGAAGTTTAAGT
TCAATAATTTTTTTTAATTTTTTTTTTTTTTTTTTTTTTTTGTTACTATAAGTTTATTATTA
TTAATTCGTAGAGCTTATCCTCGTTATCGTTATGATAAGTTAATAAATTTTTTTTGAAAA
TTAATTTTACCTTTAGTTATTATTTTATTTTTTAATAATTTTTATTTTACTAATT---ATT
CTTTCATTTATATTTTTTATTATTTTTTTATTTTTTTTCTTTTTATTTTTTAGTTTAATTTCT
AATAATTATATTTTATGATGAAGAATATTTTTAATTTTGAATATTAGATTTTTTTTTTTA
AATAAAATTTTATTAAATGAATTAAGTAATTTATATTATTTTATTATTCAAGAATTTTTA
GGTGTTTTATTTTTATTTTTTATTTTTTTTAAATTTTCAATGATTAATTTTAATATTAAAA
ATTGGAGTTTCCCCTTTTATTTTTTGAATTTTTTTTTATTATAGAAAATTTAATAAGTTAT
ATAGTTTTTTGATTTTTAACTTTTCAAAAAATGCCTTTTTTACCTTTAATTAAAGATTT-
--TTTTTTTAATTTTTTTTTTTTTTTTTTTTTTTAGGTATTTTAATAATTTATATAATTATT
TTTTATTTAAAAAATTTAAAATTTTTAGTTTTTTTAAATTCTTTAGAATCTTTTAGATGA
ATTTTAATTAATTTATTTTTTTAGTATTATAAATTTTTTTTTTTTTATTTTTTTTATTATTTT
TTTATTTTT-TTATGTTTAATTTTTT--ATTTTTAATTTTAAT---TTAATATTAAAATTT
GAAATTATTTTATTTTTTTTTAAATTTTCCTTTTTTTCCTAGTTTTTTTTTAAAGTTTTTT
AGTTTAAATTTTATTATTAATAATTTTATTTTAATGTTCTTAATTTTATTTTTAATATTT
TTTAATTTAATTAGTTTTATTTATTATTATTTTATTTATTTTTTTAAAAAATTTTAATTTT
AAAAAGGATTATTCTTATATTTCCTTTTTTTTATATTTATATTATAATTTTTTTTTTTTTGT
ATTATTAATTTTTTTTGTAATTTTTATTTATTTTTTTGATTGGTTTAATTTTTGTTTTTTTA
TTTTTTTTTATTAATTTAATTATTTCTTTTAAATTTAATTATAATTTTAAAATTTCTTCT
TTTGAAAGTGGTTTTGTAAGAATTGGAAAAAATATTTAAATCTTTAAGTTTACATTTTTTT
TTAATTTTGATTATATTTGTAATTTTTGATTTAGAAGTTATTTTTTATTATTGGTTTTTTA
GTAAGTGATTTAGAAGTGTTAATTTTATTTATAATAATTTTTTTTTTTTATATTATTTAGA
TTATATTTAGAATGATTTTTAGGTAAATTAATTTGAGTTTTATATATATTATATAATTAT
TTATTTATTTTATTAGTTTTTTTAAAAAATTTTAATATTTTATTTTTTTAATTTAATTTTT
TTATTTTTTTTTATTTTTTTAAAAAAAATTTTTTGTTTAATTTTATATTTTTTTAATATTAGC
ATATATTTAGTAATTTTAGTTTTTATAAGAATTATAATTTTAGGTTTAATTATTTTAAGT

GAATTAAATTTTAATTTAAAATTTTTAAGTATTTTATTAATTTTTGTAAGTTTAATATTT
TTTTTTAGTAGTAATTTATTTTTTTTATATTTTATATTTGAAGTTTCTTTGTTTCCTATT
TTAATTATGATTTTAGGTTATGGTTATCAAATTGAAAAAATTAATTCTTCTTATTATTTA
TTTTTTTTTACTATTTTATGTTCTATACCATTTTTTTTTTTTTATTTTTAATTTAGACTTG
AATAAAAATTTTTATTTTTTAAATTATTTTTTTTTCTTGAGAAATAGAATTAATTTTAAGT
TTAATATTTATAGTTAAATTTCCAATTTATTTTTTTCATTTTTGGTTACCCAAAGCTCAT
GTTGAAGCTCCTACTACTGCTAGAATACTTTTAGCTGGTTTATTATTAAAATTAGGATCA
GGTGGATTTTTACGTTTATTATTTTCTTTAAATTTTAGTTTTTTAGGTTTATATTTATTA
ATTTCTTTTTTAGGGATAATTATTAGTAATTTTAATTGTTTATTACAAAGAGATTTAAAA
TCTTTGTCTGCTTTTTCTTCTATTAATCATATAAGTTTAGTATTATTATTATTAGTTTTA
CTTAATTCTTTAAGAATTAAGAATAGAGTTATTATTATAGTTTCTCATGGTTTTATTTCT
ACTTTAATATTTTATTTTATTGGAGAATTTTATCATCTAAGCTTAACACGTTTAATTTTT
TATTATTCTAGTATTTTTTTAAATTCTTTAATATTTTGTTTAATTATTAGATTAACTTGA
TTATTTAATTCTGGTGTTCCTTTTTCAATTTCTTTTTTTTCTGAATTTATTTCTTTAATT
ATAATTTTTAATTTTAATTTTTTTTTTTTTTTTTTTATATTTTTATATTTTTTTTTAACA
TTTTATTATAGACTTTATTATATTGTAATTAATTTTGTTGGTAAAGAATTAATTAATTTA
AATTATAATCTTATTGTTTATAGTTTATTTTTTTTTAATTATTAATTGTAATATTTTTATT
TTTTTTATTTTTATTTAA--
---ATAAATTTT
TTTTTATTTTTAGTTTTTTTAATATTATTAATTTTTATAATATTTTTTTTATTAATTTTT
AATGGTTTATTAAATTTGAATTTGATAAATTGAAATTTTTTTTGTTTAATTTTAAATTTT
AATTTTTTTAAAATTATATTTTTTATTTTATTATTAATTGTTGTTTTAAGAGTTTTTATA
TTTTTAAATTTTTATTTAGAAGGTGAATTATTTTTTAATTATTTTTTTTTTTATTTTAATA
ATTTTTATTTTGAGAATAATTTTTTTAAATTTTAGAAATAATTATTTAATAATAATTTTA
AGATGAGATTTGTTAGGTATTTCTAGTTATTTTCTAGTTTTATTTTATAATAATTGAGAT
AGAAATATTGGTTCTATAAATGTTACTTTAACTAATCGTCTAGGTGATTATTTTATATTT
ATTTTTTTTTCTTATTTTTATTTTGTTAATTTTAATTTTGATTATTTAAATTTTTATAAT
TATATTTTTTTACTAATATTAATTTTTATAGGTTTTACTAAGAGTGCCCAATTTCCTTTT
AGAAGTTGATTACCTAAGGCTATAAGTGCACCTACTCCTGTAAGAGCTTTAGTTCATAGA
AGAACATTAGTAACAGCTGGTTTAATTTTATTTTTAAATTTTAATTTTTTTATTATTAAAT
TTAAATTTTATTTTTTTTTTTATTATTAACAGGTTTACTAACTATAATAACTGCTAGTATT
ATATCTTTAAAAGAAAAAGATTTAAAAAAAGTAGTAGCTTTAAGAACTTTATCTCAGATA
GGTTTTAGAGTTTTAATTTTAGGTTTAAATTTAAATTATTTTTGTTTAATACATTTAATT
AGTCATGCTTTATTTAAAAGTTGTTTATTTATTCAAGTAGGTTTGTTAATTTATTTTAGT
TATGGTCAACAAGATAATCGATTTTATAGAGGTCTTTCTTTTTTTTTTTAATTTTATTAAA
TGACAATTATTTATTACTTTATTTTGTTTATGTGGATTATTTTTTAGTAGTGGTTTAATA
ACAAAAGATTTAGTATTAGAATTTTTTTTTTTTTTTAATAAAAATTATTTTCTAATATTA
ATTTTTTTTTTTAGTGTTTTTTTAACTTTTTTTTTATTCTTTTATTTTATTTTTTAGGATTT
TTAAAATTAAGATTTAAAATTTATTTATTTTTTTATTAATAATTTTATAGTTTTTATATCT
TTTTTTTTATTTATTTTTTTCTTTAATAAGTTTAATATTAATTAGTAATAATTTAGTTTTA
TTTCCAGTTATAATTTTATTATTAGATTATTATTTTTTTTTTTTTTTTTTTGTTTATATTTTT
TTATTGTTAATTTTTTTTTTTATTATTAAAAAATTTTATTTTTTTTAAAATATAGTTTTATA
GTTGATTATTACGCTAAAATTTTTTCTTTTTATTTAAAAAATTTTAAATTTTTAGATAAT
TTAATTAATTTTAATTTAATTTATTTTTTTTATTTTTAATATAAAAGTTTATAAAAAATTTA
TTATTTTTTAATAATTATTATTATATTGTGTATATTTTATTATTATTTTTTTTTATTTTTT
TAG------------------------ATTAATAATTTTAAAAATTTATTTAAAAATTTA
AAAAGGTTTATTTTTTTTTTATTATTTTTTATTGATTTTTATTTTAAGATTATTAAGGTTT
TTATCTTTTGATCCTTTTAAAAGTTGTTTATTAATAATTTTAGTTTTATTAATTTTTATT
TTTTTTTTAAGTTTAAATAAAAAATATTTGGCTAGGTTATTTTATTTGTTTATTATTTTTA
AGTGGAATTTTTGTAGTTTTAATTTATTTTTCAAGTTTAAGTAGTTTTTATTATTTTTTT
TTTTCAAAAATTTTATTTTTTTTTTTTTTTTTTTTTTTTTTATATATTATAATACATTTTTT
TTATATTTTAATTTAAATTTTAGTTTATTATTTTATTATTTTTATATTTATTATTTTTTA
ATATTTATTTTTTTATTATTTTATTTTTTTATTATTTTTAAGTTTTTTTATTAATATTCAA
GGAGCTTTACGAAAATTTTATTTTTTTTTATATT-ATTTTATTTTTTTTTATATTTTTTTAGT
TTTTTATTTTTTTATTCTAAAATTTTATCGTTTAATTTTTATTTTAATTTCTTTTGAATTT
TTTATAATAGGTTTATTTTATTATTTTTCTTTTAATTTAAATCTTTTTTTATTTTTTTTAT
TTTATTTTTTTTTAGAGTTATCTCAAGTTCTTTAGGTATAATTTTTATAATTATTTTAGTG
AAGAATTTTGGTAATGATAAAGTTTTATTTTTAA
>B_cocophilus_CD3531
ATTAATTATTTTAAATTTCAAGAAGGATATAAGTATTGATTTGAAAGTTCAAATCATAAA
GATATTGGTATGTTATATTTTATTTTTGGATTTTGATCAGGAATGGTAGGGACAAGTTTA
TCTTTAATTATTCGTTTAGAGTTATCTAAGCCAGGAGTATTATTGGATAATGGACAATTA
TATAATAGAGTTTTAACTGCTCATGCTTTATTAATAATTTTTTTTATAGTTATACCTTCT
TTAGTAGGAGGGTTTGGAAATTGGTTAGTGCCATTGATATTAGGATCTCCTGATATAAGA
TTCCCACGTTTAAATAATTTAAGATTTTGATTATTACCTACTTCTTTAATATTAATTTTA
GATTCTTGTTTTGTTGATACAGGGGCAGGTACTAGATGAACTGTTTATCCTCCTTTAAGG
ACGATAGGGCATCCTGGAAGAAGGGTGGATCTAGCTATTTTTAGTTTACATTGTGCAGGA
GTTAGTTCTATTTTAGGAGGAATTAATTTTATATGTACAACTAAAAATTTACGTAGAAGT
TCAATTTCTTTAGAACATATAAATTTATTTGTTTGAACTATTTTTGTTACTGTATTTTTA
TTAGTTTTATCTTTACCAGTATTGGCAGGAGCAATTACAATATTATTGACAGATCGTAAT
ATTAATACTTCTTTTTTTGACCCAAGAATAGGAGGGAATCCTTTAATTTATCAACATTTA

TTTTGATTTTTTGGTCATCCGGAGGTTTATATTTTGATTTTACCAGCTTTTGGTATCATT
AGTCAAAGAACGCTTTATCTAACAGGGAAAAAGGAAGTATTTGGTTCATTAGGAATGGTG
TATGCTATTTTAAGAATTGGTTTAATTGGTTGTGTGGTTTGAGCACATCATATGTATACT
GTTGGCATAGATTTAGATTCTCGTGCTTATTTTACTGCTGCTACAATAGTAATTGCTGTT
CCAACAGGAGTGAAGGTTTTTAGTTGGTTAGCTACTTTATTTGGTTCAGTAATAATTTTT
CAACCTTTATTATTGTGAGTTTTAGGTTTTATTTTTTTATTTACTATTGGTGGGATAACA
GGTGTTGTATTATCTAATTCGAGGTTAGATATTATTCTTCATGATACATATTATGTTGTA
AGTCATTTCCATTATGTTTTAAGATTAGGAGCAGTATTTGGTATTTTTACTGGAGTTTCT
TTATGATGAACATTTATTATTGGGTTAGTATATAATAAAGTTATATATTGTTCTGTATTT
TTTTTATTATTTATAGGAGTAAATTTAACTTTTTTTCCTTTACATTTTGCTGGTTTACAA
GGGTACCCTCGTAAATATACTGATTACCCGGATATTTATTCAATTTGAAATATTGTGTCT
TCATTTGGAAGAATAATGAGTGTATTTGCTTTATTTTTATTTATTTATCTATTGATTGAA
TCTTTTATAAATTATCGTTTGTTTATAATTGATTATAGTACTAATTTTGGTCCAGAAAAT
TCTTTTTCTGGTTATGTTTTAGTCATAGTTATCAAT------------------------
---ATTTATAGTTTTTTCCAAGGTTATAATTTATTATTTTCTCATAGAATTTTTGCATTT
TATATAGATTGATTTCATAGTTTTAATTGTAGGTTATTATTAGGAGTATTAATTTTTGTT
AGTATATTATTTATTTATTTGATAATTAATAATTATTATTTTAAAAGTAAAAAAATTGAA
TATCAATTTGGTGAGTTATTATGTAGGTTGTTCCCTACTCTAATTTTAATTTTTCAAATA
ATTCCTTCTTTAAGAATTTTATATTATTATGGTTTAATAAATATTGATTCTAATTTAACT
ATTAAAGTTATTGGGCATCAATGATATTGGAGTTATGATTATAGGGATTTTGAAGATTTA
GAGTTTGATTCTTATATAAAGTCTATTGATTCTTTAGAAGTAGGTGATTTACGTTTATTA
GATGTAGATAATCGTTGTATTGTTCCTTCAAATTTAAACATTCGTTTTTGTATTACTTCT
GCAGATGTTATTCATTCTTGGGCTTTATCAAGTTTATCTGTAAAATTAGACGCTATGAGA
GGGATTTTAAGAATTTTAAATTATAATTTTCCTTTAGTAGGATTATTTTATGGGCAATGT
TCTGAAATATGCGGGGTTAACCATAGTTTTATACCTATCGTAGTTGAAACTACTTTGTTT
GATTTATTTAAATCTTGATGTTTATTAAATTAG---------ATTTTTTTTCATAATTTT
CATATTTTAAGGTTATCAAGGTATCCTTTCCAAGTTTTTTTATGTGTTACAGGGTTTGCT
TCTTCTTTAGTGATTTTTTTAAAATATGGTTTAATGATAGGATTATTAATTAGATTTATA
GCTTTATTTTATGTTTCTTTTTTATGATCAAAAGATATTTCTTTTGAAGGGTTAAGGGGG
TATCATAATTTTTTTGTTATAGATGGTTTTAAATTTGGTGTAATTTTATTTATTTTTAGA
GAGTTTATATTTTTTTTTGGTATTTTTTGAACTTTTTTTGATGCTTCTTTAGTTCCTAAT
CATGATTTAGGAGAAGTATGAAGTCCTTATGGTTTAACTTTAGTTAATCCTTTCGGTGTA
CCTTTATTAAATACTATTATTTTATTAAGAAGAGGGGTAACAGTAACTTGAGCTCACTAT
AGCCTTTTAAGTAATAAAGATAGAAGAGCAAGACTATTATTAACATGTTTGTTGGCTATT
TATTTTATATTAATTCAGTTAATAGAATATAAAGAAGCTGGGTTTTCTATTTCTGATGGT
ATTTATGGAAGAATTTTTTTTCTTTCAACTGGATTTCATGGGTTTCATGTTTTATGCGGG
GGTTTATTTTTATTATTTAATTTTTATCGTTTAATTAAATCTCATTTTACTTTTAATCAT
CATTTAGGGTTAGAGTTTGGGATCATTTATTGACATTTTGTTGATGTAGTATGATTATTT
TTATTTATTTTTGTTTATTGATGATCATTTTA-ATTAATAGTGTATATTTTTTAGATATT
TTTTTATTTTTTTTTTTACTTCAATTTTTATATTATTTTAATAATAATTTAATTTATATA
TTAGCTAAGAAATTTTTGTTTAGTTTAAATGATGTTTTTAGTTATTCTAAAAATTTAATT
TTAAGTAGTTTTATTTCTTTTTTTATTTTTATTTTTTTTATTAACTTGTTGTTATGGAGGT
TATTTTTGTTATTCGTTTTGTCCTTGTGGAATATTGGAGTTTACTTTATTCTTAGCTTTA
ATTTCATGACTAAGGACTTTTTTATGTTTTATTTCTAGTGAAAAAATTTCTATTTATTTT
AGCAAAGAAGGAGATTTATATTTAAAAACTTTAAGAATATTAGTAGTTGAAATTGTAAGA
GAATTTTCTCGTCCTATTGCTTTAACTGTGCGTTTAACAGTTAATATTATAGTGGGGCAT
ATAATTAGGATATCTCTTTTTATACTAGTTAAT---AGCCTAGGTTATCAATATTGGGTT
TTAACTTTATTTGCTATTTTAATAGAATGTTTTGTATTTTTTTATTCAAAGTTATATTTTT
TCACGTTTAATTTTTTTATATCTTAATGAATAAATAAATAATTTATTTTTGTTTGTTAAA
GGGCTAGTAATTAATTTACCTACTAGGAAAAGTTTAAGTTTAAATTGAAATTTTGGAAGA
CAATTGGCTATAATTTTGAGATTTCAAATTATTACAGGAACATTTTTAGTATTTTATTAT
TCTCCAGAGAGTAGTTTAGCGTTTACTAGAGTTCAATATATTATAATAGAAACTAATTTT
GGTTGAGTTTTCCGTATTTTTCATTTTAATGGTGCAAGTTTGTTTTTTATTTTTTTATAT
TTACATTTTTTTAAAGGTTTATTTTTTAGTAGTTACCGTTTAGTTAAAGTTTGAGGAAGA
GGGTTAATTATTTTTTTTATTTATTATAATAGAAGCTTTTATAGGTTATGTTTTAGTTTGA
GCTCAAATAAGATTTTGAGCTTCAGTAGTTATTACAAGATTATTAAGAGTTATTCCTATT
TGAGGAATAAAAATTGTAATTTGAATTTGGAGGGGGTTTGGAGTTTCTGGAGCTACTTTG
AAATTTTTTTTTGTTTTGCATTTTTTATTACCTTGAATATTATTAGTTTTAATTTTAGTT
CATTTGATTTTTTTACATGATACAGGAAGAACTTCTAAAGTTTATTGTCATAATGATTTT
GATAAAATTAATTTTTATTCTTTTTATTGATGAAAAGATGGTTATAATATACTTTTATGA
TTAATATTTTTTGTTTTTAGATTTTTTTATCCTTTTAATTTAGGTGACCCAGAAATGTTT
ATTGAAGCGGATCCTATAATAAGACCAGTGCATATTATTCCAGAATGATATTTTTTTATTT
GCTTATGCTATTCTTCGTGCTATTCCTAATAAAATTTTAGGGGTTATTTTTTTTACTTTTA
AGAATTTTAATTTTTTATATTTTTTTTTTTAACATAAATTATTTAAGTTTAATAAAAAAA
ATAAATTATATTTTAGTTAATTTTTTTTATTTTTATTAGAGTAATTTTAAGTTGGTTAGGA
CAATGTTTAGTAGAACCTCCGTATTTAATTTTAAGAGGGCTAGTATCTTGTTTATATTTT
TTTTTTATTATTTTAATTTTTTTAAATTATAATTTTAGTTATTTTATTTTTAAATGTATG
TTTATTATAAATTA-------TTGATTTTTTTATCTATTATTATAGAAATTTTATTAATT
TTATTTATTGTATTAAGAATTGCTTTTGTTACATTATATGAACGTCATCTTTTAGGTTTA
AGGCAAAATCGTCTTGGACCTAATAAAGTATTTTTTTTTAGGAGTTGTTCAAGCAGCGTTA
GATGGTGTAAAATTGATATCAAAAGAACAAATTTTACCTATTTTTTTCTTCTGATTTATAT

TTTTTGTTTATCCCTGGGTTAGGATTTATTTTTATATTTTTAGAGTGGATAACCCTACCT
TTTATTTTTTATTTAATAAATTTTCAATTTTCTTTTCTATTTTTAATAGTTATAGTAGGG
TTTACAGTTTATTTTACTATTATTAGAGGTTTGATAAGAAATTCTAAATTTTCTTTTTTA
GGTGCTATCCGTTCTAGAAGTCAAAGTGTATCTTTTGAGATTGGATTTTCTATTTTTTTT
TTGATTTTTATGTTTTTTTTAAATATTATAAATTTAATAAATTTATTTAATTTTAGTTTA
TTATTTTTAATTATTATATTTTTATTAATAATTTTAGCAGAATTAAATCGTGCTCCATTT
GATTTTTCTGAAGGTGAAAGAGAATTAGTTAGTGGGTTTAATACTGAATATTCAAGAGTA
AGATTCATTTTTTTATTTTTAAGTGAGTACGGAGTTTTAATTTTTTTTAGAATTTTAGGA
TCTTTAATATTTTTTGATTTTTCTTTTATTTTTAGATTTTTATTTTTGACTTTAATTTTA
TTAATTCGTAGAGCATACCCACGTTATCGTTATGATAAATTAATAAATTTTTTTTGAAAA
ATAATTTTACCTATTACTATTATTTTATTTTTTAATTATTTTTTTTTTCATTTTT---ATT
CTTTCATTTATATTTTTTTTTTTCAATTTATATTTTAATTTTTTTTTATTTGTATAATTTTT
AATAATTATATTTATTGATGAAGTTTATTTTTATTACTAAGGTTAAGGTTTTTTTTTATT
AATAAATTTTATTTAAATGATTATAGGAATTTATTTTATTTTGTTATTCAAGAAAGTCTA
GGGTTGTTTTTTTTATTTTTTTTTATTTTTAAATTTTCAATGAATAATTTTAATAATAAAA
ATTGGGGTTTCACCTTTTATTTTTTGAGGGTTTTTTATTATTGAAAATTTAAGAAGTTTT
ATATTATTTTGATTTTTAACATTTCAAAAACTTCCATTTTTACCTTTAGTAAAAGATTT-
--TATAATTAATTTTTTTTTTTTTTTTTTTTTTTGGTTTAATTTTAATTTATTTTTTTACTT
ATTTATTTAAAAAAAATTAAATTTTTAATTTTTTTGAATTCTGTAGAGTCTTTTAGATGG
ATTTTAATTAATTTTATTTTTAGGAGAATAAATTTTTTTATGATTTTTTTTTTATTATTTA
ATAAATTTTTTTATTTTTATATTTTTTTTTAAGTAAAAAAAAT---AATAAATTAAATTTA
AATTTAATTTTATTTTTTTTTAAATTTTCCTTTTTTTTTTTAGATTTTATTTAAAATTTTTT
AGTTTAAGTTTAATTAGATTTAATAATATTTTTATTTTATTTATTTTAGGGGTTATAATT
ATAAGTTTATTTTCTTTTATTTATTATTATTTTTTAATTTTTTTAAAATTTATAAAAAAT
AATATTTTTATTAATAATTTTTTTTTTTTATTTTTTTTTTTAAATAA---------------
---ATTAAAATTTTTATTTTATTAAATATTGTTATTATTAGAATAATTTTTGTTATTTTA
TTTTATTGTATTAATATAATTATATCCTTTAAATTTAATATTAACTTAAAAATTAGATCA
TTTGAGAGAGGATTTTTAAGAATTGGAAAGTTATTTAAGTCGTTTAGATTGCATTTTTTT
ATTATTATAATTATATTTGTAGTATTTGATCTAGAAATTGTTTTTGTTTTAGGGTTAATA
GTAAGAGATATAGAAAATTTAATTATTTTTTTTGTTATTTTTTTTCTTTATATTATTTAGA
CTTTATTTAGAGTGATTTTTAAAAAAATTATTATGATTATTATAGATAATTTTAGAATTA
TTATTATTTTTATTTTTATTTATTTTTTAATTTTAAAATTATAATTTATTTATTTTTTAATA
TTAATAATATTTTTATTAGTTAAAAATTATTATATAGGAATATTTTTTTTTAAGAATTAAT
AATTTTTTTTATTTTTTTTAATTTTTATAAGAATTCTAATTTTATTATTAATTTTTTTTAACT
GAATTAAATTATAATTTAAAATTATTAAGATTTTTTTTAATTTTTATTAGTTTAATTTTT
TTTGTTAGTTTGAATATTTTTTATATTTATATTTTTTTTGAACTTTCTTTATTTCCTATT
ATGATTATGATTCTAGGGTACGGGTATCAAATTGAAAAAATTAATTCTTCTTATTATTTA
TTTTTTTTTACTTTAATTTGTTCTATACCTTTTTTTTTTTTTTTATTTTTTAATTTAGATTTA
GTTAAAAGAATAAATTATTTTAATTTATTTTTTTTCTTGAGAGTTAAGTTTAGTAATTTCT
TTAATATTTTTATTAAAATTTCCAGTTTATTTTCTCCATTATTGATTACCTAAAGCTCAT
GTAGAAGCCCCTACTACTGCTAGTATATTATTAGCTGGGTTATTATTAAAAATAGGGACT
GGCGGACTTTTACGTTTTTTATATTCTTTTAATTTTTATTTTTTAGGAGTATATTTTTTT
ATTTCTTTTATTGGTATAATTTTAAGAAATTTTAATTGTATTATTCAGAGAGATTTAAAA
GCCTTAGCTGCTTTTTCTTCTATTAATCATATAAGGTTTGTTCTTCTTTCTATGGTTTTT
TTAAATAAAATCAGTCTTATTAGAAGAGTAATTATTATATTCTCTCATGGTCTAATTTCT
ACTTTAATATTTTTTTTTATTGGAGAGTTTTATCATTATGCTAATAATCGTTTAATTTAT
TATTATTCTAGAGTTTTTTTAAATTCCTTATTTTTTTGTTTATTAGTAAGTTTAACTTGG
TTATATAATGGAGGGGTCCCCTTTTCGTTATCTTTTTTTAGAGAATTTATAAGATTTGTA
ATTTTATTTAATTATAATTTATTTTATATATTTTTTGGAAGATTATATTTTTTTTATTTCT
TTTTATTATTGTTTATTTTTTATTTCTGTAAATTTTGTTGGTAAAAATTATTTTAAAATT
AATTTAAATTTAAATAGTTTTACAAGATTTTTTATTATTTGTAATTCTAATATTTTTTTT
TTTATTTTTTTTAATTTAA--
---------------------ATTTTTAAAAAAATTATTATGATTATTATAGTTAATATT
TTTTTATTTTTAGTATTTTTTTAGATTAATATTAATAGTATTATTGCTAATTTTTATAATA
AATTTTAATATATTTTTTTAATTTTTTAGATTGAAAAATTATATTTTTTTAGTTTAAATTTT
AGTTTATTTAAATTAATATTTTTTTTTTTTTATTAATTATTGTTGTATTAAGAGTAATATTA
TTTTTAAATTTTTATTTAAATGGTGAATTATATTTAAATTATTTTTTTTATAGTATTAACA
ATTTTTATTTTTAGTATAATTTTTTTTAAATTTTAGTAATAATTTTTTTATCAATAATTTTA
AGTTGAGATTTACTAGGAATTTCAAGGTATTTTTTTAGTTTTATTTTTATAATAATTGAGAC
AGAAATAATGGTTCTATAAATGTTTCTTTGACTAATCGCCTAGGTGATTATTTTATATTT
TTTTTTTTTTCTTTTTTTTTTTTTTTATAATTTTAAATTAAATTTTAATTTATATTTAAAT
TGAGTTATATTTTTTATTATTAATTATTATAGGTTTTACTAAAAGTGCTCAATTTCCTTTT
AGTAGTTGACTTCCTAAAGCTATGAGAGCTCCGACTCCTGTTAGAGCTTTAGTTCATAGA
AGAACTTTAGTAACAGCAGGGTTAGTATTATTTTTTTAATTTTAATATAATGTTAAATTTT
TTTTATTTTTTTAATAATTTTAATTTTAATTGGTTTGTTAACTATATTATTTTTCAAGGTTA
ATATCATTAATAGAAGAAGATTTAAAAAAAGTAGTGGCTTTAAGAACTTTATCTCAAATA
GGATTTAGAGTTTTAATTTTAGGTTTAGGTTTAAATTATTTTTGTTTATTACATTTGATT
AGGCATGCTTTATTTAAAAGTTGTTTATTTATTCAGGTTGGTTTATTAATTTATTTTTCT
TTTGGTCAACAAGATGGACGTTTTTATAGAAATTTAAATTTTTTTTTTTAATTTTATTCAT
TGACAAATTTTAATTACTTTATTTTGTTTATGTGGTTTATTTTTTTAGTAGTGGGTTAATA
AGTAAAGATATAATTTTAGAATTTTTTTTATTTTAATAATTTTAAATTTTTATTTTTTTTT

ATATTTTTTATTTCTATTTTTTTAACTTTTTTTTATTCTTATCGTTTATTTAAAGGTTTA
ATAAAAATTAATTTAGTATTTTTTTATATTAATTTAAATAATTATAAAATTTATTTTTCT
TTATTTTTATTTTTATTTTCTCTTATAGGTTTAATAATAATTTCTAATAATATTTTTATT
TTTCCTTCCATAATTTTATTAATAGATTTTTATAGGCCTATATTTTATATTATTTTTATTT
TTTATATTAAATTTTTTTTTTTATTATAAAAAAAAAAAATTTTTTTAAAATATAGTTTTATA
GTAGATTATTATGCTAAAATATTTTCTTTTTATTTAAAAAATATAAAATTTTTAGATTCA
TTAATTAATAAAAGATTATTTTATTTTTTTAATTTAGGATTAAAATTTAATTTTAATTTA
ATTAATATAAATTTAAAKTTTTTTTTTTTTTATATTTATTTTTATATATATATTTATTAGT
TTTTAA------------ATTTCTTATTTTAAATTAAAATATTATTTTTACAAAATAGAG
ATAAAGAAATTTTTGCAAGGTTTGCTTTTTTTAATTTTTTTTTTTATGTTTAATAAGATTT
TTTTCTTTTGATCCTTTTAAAAGTTGTTTATTAATGATTATAAGTTTATTATTTTTTTCT
TTTATTTTTAGTTTAACTTTAAATATCTGAGTTAGATATTTTATTTGTTTATTATTTTTA
AGAGGTATTTTTGTTATTTTAGTTTATTTTTCTAGATTAAGAAGTTATTATTATTTTTTT
TTTAATAAATTAATAGTAGTTTTTTTATTATTATTTTTTATTTTAAATTTAAATTTTAAT
TATTATTATTTTAAAATTAGATTTTTTTATTATAATTTATATATATTTTATTTTTTTATTA
TATATTTTATTATTATTTTATTTTTTTATTATTTTTAAGATTTTATTTAAACTTTAATGGA
GCATTGCGTAAATTTTAA---------------------ATTTTTTTTATATTTTTTTAGT
TTATTGTTTTTTATTTTTAAATTTTATCGTTTTATTTTTATTTTATTATCATTTGAATTT
TTTATAATAGGGTTATTTTATTATTTTTCTTTAAATTTAATAAGTATAATATTTTTTTTAT
TTTTTAATTTTTAGAGTAATTTCTAGAATTTTAGGTATAGTTATGATAATTTATATAGTA
AAATGTTATGGATTTGATAAAATTTTATTTTAA
>B_cocophilus_CD3845
ATTAATTATTTTAAATTTCAAGAAGGATATAAGTATTGATTTGAAAGTTCAAATCATAAA
GATATTGGTATGTTATATTTTATTTTTGGATTTTGATCAGGAATGGTAGGGACAAGTTTA
TCTTTAATTATTCGTTTAGAGTTATCTAAGCCAGGAGTTTTATTGGATAATGGACAATTA
TATAATAGAGTTTTAACTGCTCATGCTTTATTAATAATTTTTTTTATAGTTATACCTTCT
TTAGTAGGAGGGTTTGGAAATTGGTTAGTGCCATTGATATTAGGATCTCCTGATATAAGA
TTTCCACGTTTAAATAATTTAAGATTTTGATTATTACCTACTTCTTTAATATTAATTTTA
GATTCTTGTTTTGTTGATACAGGGGCAGGTACTAGATGAACTGTTTATCCTCCTTTAAGG
ACGATAGGGCATCCTGGAAGAAGGGTGGATCTAGCTATTTTTAGTTTACATTGTGCAGGA
GTTAGTTCTATTTTAGGAGGAATTAATTTTATATGTACAACTAAAAATTTACGTAGAAGT
TCAATTTCTTTAGAACATATAAATTTATTTGTTTGAACTATTTTTGTTACTGTATTTTTA
TTAGTTTTATCTTTACCAGTATTGGCAGGAGCAATTACAATATTATTGACAGATCGTAAT
ATTAATACTTCTTTTTTTGATCCAAGAATAGGAGGGAACCCTTTAATTTATCAACATTTA
TTTTGATTTTTTGGTCATCCGGAGGTTTATATTTTGATTTTACCAGCTTTTGGTATCATT
AGTCAAAGAACGCTTTATTTAACAGGGAAAAAGGAAGTATTTGGTTCATTAGGAATGGTG
TATGCTATTTTAAGAATTGGTTTAATTGGTTGTGTGGTTTGAGCACATCATATGTATACT
GTTGGTATAGATTTAGATTCTCGTGCTTATTTTACTGCTGCTACAATAGTAATTGCTGTT
CCAACAGGAGTGAAGGTTTTTAGTTGGTTAGCTACTTTATTTGGTTCAGTAATAATTTTT
CAACCTTTATTATTGTGAGTTTTAGGTTTTATTTTTTTTATTTACTATTGGTGGGATAACA
GGTGTTGTATTATCTAATTCGAGGTTAGATATTATTCTTCATGATACATATTATGTTGTA
AGTCATTTCCATTATGTTTTAAGATTAGGAGCAGTGTTTGGTATTTTTACTGGAGTTTCT
TTATGATGAACATTTATTATTGGGTTAGTATATAATAAAGTTATATATTGTTCTGTATTT
TTTTTATTATTTATAGGAGTAAACTTAACTTTTTTTCCTTTACATTTTGCTGGTTTACAA
GGGTACCCTCGTAAATATACTGATTACCCGGATATTTATTCAATTTGAAATATTGTGTCT
TCATTTGGAAGAATAATAAGTGTATTTGCTTTATTTTTATTTATTTATCTATTGATTGAA
TCTTTTATAAATTATCGTTTGTTTATAATTGATTATAGTACTAATTTTGGTCCAGAAAAT
TCTTTTTCTGGTTATGTTTTTAGTCATAGTTATCAATT----------------------
---ATTTATAGTTTCTTTCAAGGTTATAATTTATTATTTTCTCATAGAATTTTTGCATTT
TATATAGATTGATTTCATAGTTTTAATTGTAGGTTATTATTAGGAGTATTAATTTTTGTT
AGTATATTATTTATTTATTTGATAGTTAATAATTATTATTTTAAAAGTAAAAAAATTGAA
TATCAATTTGGTGAGTTATTATGTAGGTTGTTTCCTACTTTAATTTTAATTTTTCAAATA
ATTCCTTCTTTAAGAATTTTATATTATTATGGTTTAATAAATATTGATTCTAATTTAACT
ATTAAAGTTATTGGGCATCAATGATATTGGAGTTATGATTATAGGGATTTTGAAGATTTA
GAGTTTGATTCTTATATAAAGTCTATTGATTCTTTAGAAGTAGGTGATTTACGTTTATTA
GATGTAGATAATCGTTGTATTGTTCCTTCAAATTTAAACATTCGTTTTTGTATTACTTCT
GCAGATGTTATTCATTCTTGGGCTTTATCAAGTTTATCTGTAAAATTAGACGCTATGAGA
GGGATTTTAAGAATTTTAAATTATAATTTTCCTTTAGTAGGATTATTTTATGGGCAATGT
TCTGAAATATGCGGGGTTAACCATAGTTTTATACCTATTGTAGTTGAAACTACTTTGTTT
GATTTATTTAAATCTTGATGTTTATTAAATTAG---------ATTTTTTTTCATAATTTT
CATATTTTAAGGTTATCAAGGTATCCTTTCCAAGTTTTTTTATGTGTTACAGGGTTTGCT
TCTTCTTTAGTGATTTTTTTAAAATATGGTTTAATGATAGGATTATTAATTAGATTTATA
GCTTTATTTTATGTTTCTTTTTTATGATCAAAAGATATTTCTTTTGAAGGGTTAAGGGGG
TATCATAATTTTTTTGTTATAGATGGTTTTAAATTTGGTGTAATTTTATTTATTTTTAGA
GAGTTTATATTTTTTTTTGGTATTTTTTGAACTTTTTTTGATGCTTCTTTAGTTCCTAAT
CATGATTTAGGAGAAGTATGAAGTCCTTATGGTTTAACTTTAGTTAATCCTTTCGGTGTA
CCTTTATTAAATACTATTATTTTATTAAGAAGAGGGGTAACAGTAACTTGAGCTCATTAT
AGCCTTTTAAGTAATAAAGATAGAAGAGTAAGACTATTATTAACATGTTTGTTAGCTATT
TATTTTATATTAATTCAGTTAATAGAATATAAAGAAGCTGGGTTTTCTATTTCTGATGGT
ATTTATGGAAGAATTTTTTTTCTTTCAACTGGATTTCATGGGTTTCATGTTTTATGCGGG
GGTTTATTTTTATTATTTAATTTTTATCGTTTAATTAAATCTCATTTTACTTTTAATCAT

CATTTAGGGTTAGAGTTTGGGATTATTTATTGACATTTTGTTGATGTAGTATGATTATTT
TTATTTATTTTTGTTTATTGATGATCATTTTA-ATTAATAGTGTATATTTTTTAGATATT
TTTTTATTTTTTTTTTTACTTCAATTTTTATATTATTTTAATAATAATTTAATTTATATA
TTAGCTAAGAAATTTTTGTTTAGTTTAAATGATGTTTTTAGTTATTCTAAAAATTTAATT
TTAAGTAGTTTTATTTCTTTTTTTATTTTTATTTTTTTTATTAACTTGTTGTTATGGAGGT
TATTTTTGTTATTCGTTTTGTCCTTGTGGAATATTGGAGTTTACTTTATTCTTAGCTTTA
ATTTCATGACTAAGGACTTTTTTATGTTTTATTTCTAGTGAAAAAATTTCTATTTATTTT
AGCAAAGAAGGAGATTTATATTTAAAAACTTTAAGAATATTAGTAGTTGAAATTGTAAGA
GAATTTTCTCGTCCTATTGCTTTAACTGTGCGTTTAACAGTTAATATTATAGTGGGGCAT
ATAATTAGGATATCTCTTTTTATATTAGTTAAT---AGCCTAGGTTATCAATATTGGTTT
TTAACTTTATTTGCTATTTTAATAGAATGTTTTGTATTTTTTTATTCAAAGTTATATTTTT
TCACGTTTAATTTTTTTATATCTTAATGAATAAATAAATAATTTATTTTTTGTTTGTTAAA
GGGCTAGTAATTAAGTTACCTACTAGGAAAAGTTTAAGTTTAAATTGAAATTTTGGAAGA
CAATTGGCTATAATTTTGAGATTTCAAATTATTACAGGAACATTTTTAGTATTTTATTAT
TCTCCAGAGAGTAGTTTAGCGTTTACTAGAGTTCAATATATTATAATAGAAACTAATTTT
GGTTGAGTTTTCCGTATTTTTCATTTTAATGGTGCAAGTTTGTTTTTTATTTTTTTATAT
TTACATTTTTTTAAAGGTTTATTTTTTAGTAGTTACCGTTTAGTTAAAGTTTGAGGAAGA
GGGTTAATTATTTTTTTTATTCATTATAATAGAAGCTTTTATAGGTTATGTTTTAGTTTGA
GCTCAAATAAGATTTTGAGCTTCAGTAGTTATTACAAGATTATTAAGAGTTATTCCTATT
TGAGGAATAAAAATTGTAATTTGAATTTGGAGGGGGTTTGGAGTTTCTGGAGCTACTTTG
AAATTTTTTTTTGTTTTGCATTTTTTATTACCTTGAATATTATTAGTTTTAATTTTAGTT
CATTTGATTTTTTTACATGATACAGGAAGAACTTCTAAAGTTTATTGTCATAATGATTTT
GATAAAATTAATTTTTATTCTTTTTATTGATGAAAAGATGGTTATAATATACTTTTATGA
TTAATATTTTTTGTTTTTAGATTTTTTTATCCTTTTAATTTAGGTGACCCAGAAATGTTT
ATTGAAGCGGATCCTATAATAAGACCAGTACATATTATCCCAGAATGATATTTTTTATTT
GCTTATGCTATTCTTCGTGCTATTCCTAATAAAATTTTAGGAGTTATTTTTTTACTTTTA
AGAATTTTAATTTTTTATATTTTTTTTTTTAACATAAATTATTTAAGTTTAATAAAAAAA
ATAAATTATATTTTAGTTAATTTTTTTATTTTTATTAGAGTAATTTTAAGTTGGTTAGGA
CAATGTTTAGTAGAACCTCCTTATTTAATTTTAAGAGGGCTAGTATCTTGTTTATATTTT
TTTTTTATTATTTTAATTTTTTTAAATTATAATTTTAGTTATTTTATTTTTAAATGTATG
TTTATTATAAATTA-------TTGATTTTTTTATCTATTATTATAGAAATTTTATTAATT
TTATTTATTGTATTAAGAATTGCTTTTGTTACATTATATGAACGTCATCTTTTAGGTTTA
AGGCAAAATCGTCTTGGACCTAATAAAGTATTTTTTTTAGGAGTTGTTCAAGCAGCGTTA
GATGGTGTAAAATTGATATCAAAAGAACAAATTTTACCTATTTTTTCTTCTGATTTATAT
TTTTTGTTTATCCCTGGGTTAGGATTTATTTTTATATTTCTAGAGTGGATAGCCCTACCT
TTTATTTTTTATTTAATAAATTTTCAATTTTCTTTTCTATTTTTAATAGTTATAGTAGGG
TTTATAGTTTATTTTACTATTATTAGAGGTTTGATAAGAAATTCTAAATTTTCTTTTTTA
GGTGCTATCCGTTCTAGAAGTCAAAGTGTATCTTTTGAAATTGGATTTTCTATTTTTTTT
TTGATTTTTATGTTTTTTTTAAATATTTTAAATTTAATAAATTTATTTAATTTTAGTTTA
TTATTTTTAATTATTATATTTTTATTAATAATTTTAGCAGAATTAAATCGTGCTCCATTT
GATTTTTCTGAAGGTGAAAGAGAATTAGTTAGTGGATTTAATACTGAATATTCAAGAGTA
AGATTCATTTTTTTATTTTTAAGTGAGTACGGAGTTTTAATTTTTTTTAGAATTTTAGGA
TCTTTAATATTTTTTGATTTTTCTTTTATTTTTAGATTTTTATTTTTGACTTTAATTTTA
TTAATTCGTAGAGCGTATCCACGTTATCGTTATGATAAATTAATAAATTTTTTTTGAAAA
ATAATTTTACCTACTACTATTATTTTATTTTTTAATTATTTTTTTTTCATTTTT---ATT
CTTTCATTTATATTTTTTTTTTCAATTTATATTTTAATTTTTTTTATTTGTATAATTTTT
AATAATTATATTTATTGATGAAGTTTATTTTTATTATTAAGGTTAAGGTTTTTTTTTATT
AATAAATTTTATTTAAATGATTATAGGAATTTATTTTATTTTGTTATTCAAGAAAGTCTA
GGGTTGTTTTTTTTATTTTTTTTTATTTTTAAATTTTCAATGAATAATTTTAATAATAAAA
ATTGGGGTTTCACCTTTTATTTTTTGAGGGTTTTTTATTATTGAAAATTTAAGAAGTTTT
ATATTATTTTGATTTTTAACATTTCAAAAACTTCCATTCTTACCTTTAGTAAAAGATTT-
--TATAATTAATTTTTTTTTTTTTTTTTTTTTTTTGGTTTAATTTTAATTTATTTTTTACTT
ATTTATTTAAAAAAAATTAAATTTTTAATTTTTTTGAATTCTGTAGAGTCTTTTAGATGG
ATTTTAATTAATTTTATTTTTAGGAGAATAAATTTTTTTATGATTTTTTTTTTATTATTTA
ATAAATTTTTTTATTTTTATATTTTTTTTAAGTAAAAAAAAT---AATAAATTAAATTTA
AATTTAATTTTATTTTTTTTTAAATTTTCCTTTTTTTTTTTAGATTTTATTTAAAATTTTTT
AGTTTAAGTTTAATTAGATTTAATAATATTTTTATTTTATTTATTTTAGGGGTTATAATT
ATAAGTCTATTTTCTTTTATTTATTATTATTTTTTTAATTTTTTTTAAAATTTATAAAAAAT
AATATTTTTATTAATAATTTTTTTTTTTATTTTTTTTTTTAAATAA----------------
---ATTAAAATTTTTATTTTATTAAATATTGTTATTATTAGAATAATTTTTGTTATTTTA
TTTTATTGCATTAATATAATTATATCCTTTAAATTTAATATTAACTTAAAAATTAGATCA
TTTGAGAGAGGATTTTTAAGAATTGGAAAGTTATTTAAGTCATTTAGATTGCATTTTTTT
ATTATTATAATTATATTTGTAGTATTTGATCTAGAAATTGTTTTTATTTTAGGGTTATTA
GTAAGAGATATAGAAAATTTAATTATTTTTTTTTGTTATTTTTTTCTTTATATTATTTAGA
CTTTATTTAGAGTGATTTTTAAAAAAATTATTATGATTATTATAGATAATTTTAGAATTA
TTATTATTTTTATTTTTATTTATTTTTAATTTTAAAATTATAATTTATTTATTTTTTAATA
TTAATAATATTTTTATTAGTTAAAAATTATTATATAGGAATATTTTTTTTTAAGAATAAAT
AATTTTTTTATTTTTTTTAATTTTTATAAGAATTCTAATTTTATTATTAATTTTTTTTAACT
GAATTAAATTATAATTTAAAATTATTAAGATTTTTTTTTAATTTTTATTAGTTTAATTTTT
TTTGTTAGTTTGAATATTTTTTATATTTATATTTTTTTTTGAACTTTCTTTATTTCCTATT
ATGATTATGATTCTAGGGTACGGGTATCAATTGAAAAAATTAATTCTTCTTATTATTTA

TTTTTTTTTACTTTAATTTGTTCTATACCTTTTTTTTTTTTTTATTTTTAACTTAGATTTA
GTTAAAAGAATAAATTATTTTAATTTATTTTTTTCTTGAGAGTTAAGTTTAGTAATTTCT
TTAATATTTTTATTAAAATTTCCAGTTTATTTTCTCCATTATTGATTACCCAAAGCTCAT
GTAGAAGCCCCTACTACTGCTAGTATATTATTAGCTGGGTTATTACTAAAAATAGGGACT
GGCGGACTTTTACGTTTTTTATATTCTTTTAATTTTTATTTTTTAGGAGTATATTTTTTT
ATTTCTTTTATTGGTATAATTTTAAGAAATTTTAATTGTATTATTCAGAGGGATTTAAAA
GCCTTAGCTGCTTTTTCTTCTATTAACCATATAAGGTTTGTTCTTCTTTCTATGGTTTTT
TTAAATAAAATCAGTCTTATTAGAAGAGTAATTATTATATTCTCTCATGGTTTAATTTCT
ACTTTAATATTTTTTTTTATTGGAGAGTTTTATCATTATGCTAATAATCGTTTAATTTAT
TATTATTCTAGAGTTTTTTTAAATTCCTTATTTTTTTGTTTATTAGTAAGTTTAACTTGG
TTATATAATGGAGGGGTCCCCTTTTCGTTATCTTTTTTTAGAGAATTTATAAGATTTGTA
ATTTTATTTAATTATAATTTATTTTATATATTTTTTGGAAGATTATATTTTTTTTATTTCT
TTTTATTATTGTTTATTTTTTTATTTCTGTAAATTTTGTTGGTAAAAATTATTTTAAAATT
AATTTAAATTTAAATAGTTTTACAAGATTTTTTATTATTTGTAATTCTAATATTTTTTTT
TTTATTTTTTTAATTTAA--
---------------------ATTTTTAAAAAAATTATTATGATTATTATAGTTAATATT
TTTTTATTTTTAGTATTTTTTAGTTTAATATTAATAGTATTATTGCTAATTTTTATAATA
AATTTTAATATATTTTTTAATTTTTTAGATTGAAAAATTATATTTTTTAGTTTAAATTTT
AGTTTATTTAAATTAATATTTTTTTTTTTTATTAATTATTGTTGTATTAAGAGTAATATTA
TTTTTAAATTTTTATTTAAATGGTGAATTATATTTAAATTATTTTTTTATAGTATTAACA
ATTTTTATTTTTAGTATAATTTTTTTAAATTTTAGTAATAATTTTTTATCAATAATTTTA
AGTTGAGATTTACTAGGAATTTCAAGGTATTTTTTAGTTTTATTTTATAATAATTGAGAC
AGAAATAATGGTTCTATAAATGTTTCTTTGACTAATCGCTTAGGTGATTATTTTATATTT
TTTTTTTTTCTTTTTTTTTTTTTTATAATTTTAAATTAAATTTTAATTTATATTTAAAT
TGAGTTATATTTTTATTATTAATTATTATAGGTTTTACTAAAAGTGCTCAATTTCCTTTT
AGTAGTTGACTTCCTAAAGCTATGAGAGCTCCGACTCCTGTTAGAGCTTTAGTTCATAGA
AGAACTTTAGTAACAGCAGGGTTAGTATTATTTTTTAATTTTAATATAATGTTAAATTTT
TTTTATTTTTTAATAATTTTAATTTTAATTGGTTTGTTAACTATATTATTTTCAAGGTTA
ATATCATTAATAGAAGAAGATTTAAAAAAAGTAGTGGCTTTAAGAACTTTATCTCAAATA
GGATTTAGAGTTTTAATTTTAGGTTTAGGTTTAAATTATTTTTGTTTATTACATTTGATT
AGGCATGCTTTATTTAAAAGTTGTTTATTTATTCAGGTTGGTTTATTAATTTATTTTTCT
TTTGGTCAACAAGATGGGCGTTTTTATAGAAATTTAAATTTTTTTTTTAATTTTATTCAT
TGACAAATTTTAATTACTTTATTTTGTTTATGTGGTTTATTTTTTAGTAGTGGGTTAGTA
AGTAAAGATATAATTTTAGAATTTTTTTATTTTAATAATTTTAAATTTTTATTTTTTTTT
ATATTTTTTATTTCTATTTTTTTAACTTTTTTTTATTCTTATCGTTTATTTAAAGGTTTA
ATAAAAATTAATTTAGTATTTTTTTATATTAATTTAAATAATTATAAAATTTATTTTTCT
TTATTTTTATTTTTATTTTCTCTTATAGGTTTAATAATAATTTCTAATAATATTTTTATT
TTTCCTTCCATAATTTTATTAATAGATTTTTATAGGCCTATATTTTATATTATTTTATTT
TTTATATTAAATTTTTTTTTTTATTATAAAAAAAAAAAATTTTTTTAAAATATAGTTTTATA
GTAGATTATTATGCTAAAATATTTTCTTTTTATTTAAAAAATATAAAATTTTTAGATTTA
TTAATTAATAAAAGATTATTTTATTTTTTTTAATTTAGGATTAAAATTTAATTTTAATTTA
ATTAATATAAATTTAAATTTTTTTTTTTTTTATATTTATTTTTATATATATATTTATTAGT
TTTTA-------------ATTTCTTATTTTAAATTAAAATATTATTTTTACAAAATAGAG
ATAAAGAAATTTTTGCAAGGTTTACTTTTTTTAATTTTTTTTTTTATGTTTAATAAGATTT
TTTTCTTTTGATCCTTTTAAAAGTTGTTTATTAATGATTATAAGTTTATTATTTTTTTCT
TTTATTTTTAGTTTAACTTTAAATATCTGAGTTAGATATTTTATTTGTTTATTATTTTTA
AGAGGTATTTTTGTTATTTTAGTTTATTTTTCTAGATTAAGAAGTTATTATTATTTTTTT
TTTAATAAATTAATAGTAGTTTTTTTATTATTATTTTTTATTTTAAATTTAAATTTTAAT
TATTATTATTTTAAAATTAGATTTTTTTATTATAATTTATATATATTTTATTTTTTTATTA
TATATTTTATTATTATTTTATTTTTTTATTATTTTTAAGATTTTATTTAAACTTTAATGGA
GCATTGCGTAAATTTA-----------------------ATTTTTTTTATATTTTTTAGT
TTATTGTTTTTTATTTTTAAATTTTATCGTTTTATTTTTATTTTATTATCATTTGAATTT
TTTATAATAGGGTTATTTTATTATTTTTCTTTAAATTTAATAAGTATAATATTTTTTTAT
TTTTTAATTTTTAGAGTAATTTCTAGAATTTTAGGTATAGTTATGATAATTTATATAGTA
AAATGTTATGGATTTGATAAAATTTTATTTTAA
>B_michalskii_CD3643
TTAATAATAAAAATTTTAATTTTTAATTTTATAAAATGATTTGAAAGTTCTAATCATAAA
GATATTGGAATATTATATTTTATTTTTGGTTTTTGTTCTGGTATAATTGGTACAAGATTA
TCAATAATTATTCGTTTTGAGTTAACTAAACCTGGTTTTTTTTTAAATAATGGTCAATTA
TATAATAGTATTATTACAGCTCATGCTTTATTAATAATTTTTTTTATAGTTATACCATCT
ATAGTTGGAGGTTTTGGTAATTGATTATTACCTTTAATATTAGGATCTCCTGATATAAGT
TTTCCACGTTTAAATAATTTAAGTTTTTGATTATTACCAGTTTCTTTAATATTATTATTA
GATGCTTGTTTTGTTGATATAGGGGCTGGTACAAGTTGAACTGTTTATCCTCCTTTAAGA
ACTTTAGGACATCCTGGTAGAAGAGTTGATTTAGCAATTTTTAGTTTACATTGTGCTGGT
GTTAGTTCAATTTTAGGTGGTATTAATTTTATATGTACTACTAAAAATTTGCGTAGTAGT
TCTATTTCTTTAGAGCATATAAGTTTATTTGTTTGAACAATTTTTGTTACAGTTTTTTTA
TTAGTTTTGTCTTTACCAGTTTTAGCAGGAGCAATTACTATATTACTTACTGATCGTAAT
ATTAATACTTCATTTTTTGATCCAAGAATAGGAGGTAATCCTTTAATTTATCAACATTTA
TTTTGATTTTTTGGACACCCTGAAGTTTATATTTTAATTTTACCAGCTTTTGGTATTATT
AGACAAAGTACGTTATTTTTAACTGGTAAAAAAGAAGTTTTTGGTTCTTTAGGTATAGTA
TATGCTATTTTAAGAATTGGTTTAATTGGTTGTGTAGTATGAGCTCATCATATGTATACA

GTTGGTATAGATTTAGATTCTCGTGCTTATTTTACTGCAGCTACTATAGTAATTGCTGTT
CCTACTGGTGTTAAAGTTTTTAGTTGATTAGCTACTTTATTTGGTTCAATTTTAATTTTT
CAACCTTTATTATTATGGGTTTTAGGTTTTATTTTTTTATTTACTATTGGTGGTTTAACT
GGTGTAATTTTATCAAATTCTAGTTTGGATATTATTTTACATGATACTTATTATGTAGTT
AGTCATTTTCATTATGTTTTAAGTTTAGGAGCTGTTTTTGGTATTTTTACAGGAGTTGCA
TTATGATGAACTTTTATTATAGGATTATTGTATGATAAAGTTTTATTAAGTTCAATTTTT
ATTTTAATATTTATTGGTGTTAATTTAACTTTTTTTCCTTTACATTTTTCAGGTTTACAA
GGATATCCTCGTAAATATATTGATTTTCCAGATATTTATTCTTTATGAAACATTGTTTCT
TCTTTTGGTAGTTTATTAAGAGTTTTTTCTTTATTTTTATTTATTTATTTTATATTTGAT
TCTTATTTTAATTTTCGTTTATTTTTAATTGATTTGAGTAGAAATTATAGTTCTGAAGTT
TCTTTAGCAGGTTATACTTTTAATCATAGTTATCAAAGAGAAATATTTTTTGTTGTTAAG
TAAATAAATAATTTTTTTCAAGGCTTAAATTTATTATTTTCTGGTAGTTTTTTTTTCTTTT
TATATAGATTGGTTTCATAGTTTTAATTGTAGTTTATTATTAGGAGTATTAATTTTTGTT
AGAATACTGTTTATATTTTTAATTTTTTGTAATTTTTATTTTAAAGGTAAAAAAATTGAG
TATCAATTTGGAGAATTATTATGTAGTTTATTTCCTACTTTAATTTTATTATTTCAAATA
ATCCCTTCTTTGAGTTTATTATATTACTATGGTTTAATAAATATTGATTCTAATTTAACT
GTTAAAGTTATTGGACATCAGTGATATTGAAGATATGATTATAGTGATTTTAAAGATTTA
GAATTTGATTCATATATAAAATCTTTAGATATATTAGAATTAGGTGAATTACGTTTATTG
GATGTTGATAATCGTTGTGTCTTACCAGTAGATTTAAATATTCGTTTTTGTATTACTTCT
GCAGATGTAATTCATGCTTGGTCATTGTCAAGTTTATCAATTAAATTAGATGCTATAAGA
GGTATTCTAAGTATTTTGAATTATAATTTTGCTTTAGTAGGTTTATTTTATGGACAATGT
TCTGAAATTTGTGGTGCAAATCATAGTTTTATGCCAGTAGTTGTTGAAGTTACTTTATTT
GATTTTTTTAAAAATTGATGTTTATTAAATTAGATTTTTTACATTATTTTTCATAATTTT
CATATTTTAAGTCTTTCTAGATATCCATATCAAGTTTTTTTATGTGTTTTAGGTTTAACT
TCATCTTTAGTAATTTTTTTGAAGTATGGTTTATTATTTGGTTTTTTTTTTAGTTTTATT
ATTTTATTTTATATTTCTTTTCTTTGATCTAAAGATATTTCTTTTGAAGGTTTAAGAGGT
TATCATAATTTTTTTGTTATAGATGGTTTTAAATTAGGAGTTTTATTGTTTATTTTTAGA
GAGTTTATATTTTTTTTTAGTATTTTTTGAACTTTTTTTGATGCTTCTTTGGTTCCTTGT
CACGATTTAGGTGAAATATGGAGACCATATGGTTTGGTTTTGGTTAATCCTTTTGGAGTG
CCTTTATTAAATACTATTATTTTATTAAGTAGAGGTGTAACTGTAACTTGATGTCATTTT
AGTTTACTAAGAAATAAAGATAGTTTTATTGGTTTATTGTTGACTTGTTTTTTAGCTTTA
TATTTTATATTAATTCAATTAATAGAATATAAGGAGGCTAGTTTTTCAATTTCAGATGGA
ATTTATGGTAGGATTTTTTTTCTTTCTACTGGTTTTCATGGTTTTCATGTTTTATGTGGT
GGATTATTTTTATTATTTAATTTATGACGATTATTACTTTCTCATTTTACTTTTAATCAT
CATTTAGGTTTAGAGTTTGCTATTATTTATTGACATTTTGTTGATGTTGTATGGTTATTT
TTATTTGTATTTGTTTATTGATGATCATTTTA-ATTAATAATGTTTATTTTTTAGATATT
TTTTTGTTTTTATTTCTTTTACATTTTATTTTTTATTTAAATTTTAGTTTATTAAAAGAG
TTTTTTTTTATTTTTTTGTTTAGTTTAAATAATGTTTTTAGATATTCTAAGGAATTATTA
TTAAGAAGTTTTATTTCAGTTTTTGTATTTTTGTTTTTATTAACTTGTTGTTATGGGGGG
TATTTTTGTTATTCTTTTTGTCCATGTGGTATAGTAGAATTTACTTTAGCTTTTGCTTTA
ATTTCGTGGTTAAGAACTTTTCTTTGTTTTATTTCAAGTGAGAAAATTTCTATTTATTTT
AGTAAAGGGGGTGATTCTTTTTTAAAGACTTTAAGAATATTAGTAGTTGAAATTGTTAGT
GAGTTTTCTCGTCCAGTGGCTTTAACAGTTCGTTTAACAGTGAATATTATAGTTGGACAT
ATAATTAGGATATCTATTTTTATATTAGTAGAA---TCTTTAGGATATAAGTATTTCTTT
TTTACTATTTTTGCTATTTTAATAGAATGTTTTGTTTTTATTATTCAAAGTTATATTTTT
TCTCGTTTAATTTTTTTATATTTAAATGAATAAATAAAATTTTTATTTTTATTTGTTAAA
GGTTTATTAGTAAATTTACCATCAAGGAAAAGTTTAAGTCTTAATTGAAATTTTGGTAGA
CAGTTAGCTTTTATTTTAATTTTTCAAATTTTAACTGGTACATTTTTTGGTTTTTTATTAT
TCTGCTGATAGTCTTTTGGCTTTTAATAGTGTTCAGTATATTATAATGGAGGTTAATTAT
GGTTGGGTATTTCGAATTTTTCATTTTAATGGAGCAAGTTTATTTTTTTATTTTTTTTGTAT
TTACATTTTTTTAAGGGTTTATTTTTTTTTTAGTTATCGTCTAATAAAGGTTTGAATAAGT
GGTTTATTACTTTTCTTATTGATTATAATAGAGGCTTTTATGGGTTATGTTTTAGTATGA
GCACAAATAAGTTTTTGAGCTTCTGTAGTAATTACTAGTTTATTAAGAGTAATTCCTATT
TGAGGTCCGGTTATTGTTTCTTGAATTTGAAGGGGGTTTAGTGTTTCTGGTGCTACTTTA
AAATTTTTTTTTGTCTTACATTTTTTATTACCTTGATTTTTTTTTAGTATTAGTTATTATT
CATTTAATTTTTTTACATGAAAGAGGGAGAAGATCTAAGGTTTTATGTTGTGGTGATTTT
GATAAAATTAATTTTTATGTTTATTATTGATGAAAAGATGGTTATAATTTTTTTATTGTGA
TTAATGTTTTTTATTTTTAGTTTACTTTTACCTTTTGTTTTAGGGGATCCTGAAATATTT
ATTGAGGCGGATCCTATAATAAGACCTGTACATATTATTCCTGAATGATATTTTTTTATTC
GCTTATGCTATTTTGCGTGCTATTCCTAATAAGATTTTGGGGGTAGTTTTTTTTATTATTG
AGAATTTTAATTTTTTATTTTTTTATTTTAGATTTTAATTATTTAAGTGTTATAAAAAAA
AGTAATAATTTATTAGTTTATTTTTTTATTTTTTTAAGTTTTATTTTGAGTTGATTGGGT
CAATGTTTAGTAGAGTATCCATTTTTATTGTTAAGTGGTTTAGTTTCTTTTATTTATTTT
TTATTAATTTTTATTTTATTATTTAATTATTTATTTTCTAAATATTATTTTAAATATGTA
TTTATTATAATTTA-TATTTCTTGATTGTTCTTATGGTTATTATAGAGTTATTAATAATT
TTATTTATTATTTTAAGAATTGCTTTTATTACTTTATATGAGCGTCATTTATTAGGTTTA
AGTCAAAATCGTTTAGGCCCTAATAAGGTTTTTTTTTTAGGTATAGTACAGGCAGTTTTA
GATGGGGTTAAATTAATAAGAAAGGAACAAATTTTACCAATTTTTTCTTCTGATTTATAT
TTTTTATTAGTACCAGGAGTTTCTTTTATTTTTTATGTTCTTGGAGTGATTAAGGTTGCCA
TTTTTTTTTTTTTTTATTAATTTTCAATTTTCTTTTATTTTTTTAATAAGTTTAGTTGGT
TTTTCTGTTTATTTTACTATTGTTAGAGGTTTATTAAGTAATTCTAAATATTCTTTTTTA

GGGGCTGTTCGTGCAAGTAGTCAAAGTGTTTCTTTTGAAATTGCTTTTTCTATTTATATT
TTTTGTTTTATAGTTTTTTTATGTTCTTTTAATTTTTTAAATTTATTTAATTTTAGTCTT
TTTTTATTATTTTATCCTTTTTTGTTAATAGTTTTGGCTGAATTAAATCGTGCTCCATTT
GATTTTTCAGAGAGTGAAAGTGAATTAGTAAGAGGTTTTAATACTGAGTATTCTAGGGTT
GGTTTTATTTTTTTATTTTTAGGGGAATATGGGGTGTTGATTTTTTTTAGTGTTTTAAGT
AGTGTTTTATTTTTTAATTTTAGTTTTTTTTTTGTTTTTTTTTATTTTAGAATTTTATTA
ATTATTCGTAGAGTATACCCTCGTTATCGTTATGATTTTTTAATAGAATTATTTTGGTTA
AAAATTTTACCAATTTCTATTTTTTTTATATTTTTTTATTTTTTTAATAATTTAA---GTT
CTTTCATTTATTTTTTTTTGTTGTTTTTATTTTCTTATTTTTTTTTGTAGGTTAAGTTTT
AATAATTATATTTATTGGTGACTAATTTTTTTATTACTTAGTATTAGTTTTTTTTTTATT
AATAAATTTTATTTAAATGATTATAGAAATTTAAATTATTTTATTGTACAAGAAAGATTA
GGACTATTTTTTTTAGTTTTTTTTTTTTAGGGGACTACAGTGATTAATTTTAATATGTAAA
GTTGGTATTTCCCCTTTTATTTTTTGATTGTTTTTTGTAGTTGATAATATAAATTATTTA
TCTATTTTTTGATTTTTAACTTTTCAAAAAATACCTTTTTTACCTATTTTAAAAGAATT-
--TTTATTAAATTTTTATTTTATTTTTTTTCTTGGTTTATTAATACTTTATTTAATTTTA
TTATTAGTAAAAAAGGTTAAATTTTTAGTAGTTTTAAATTCTGTAGAAACTTTTAATTGA
CTTTTGATTATTTTTTTTTTTTAATTTTTTTAATTTTTTTTTTTTTATTCTTTTATTATTTT
TTTAGTTTTTTTTTACTTTCAGAAAATTTAATAAATTTAGGA---AATAATATTAATCTA
GAACTTATTATTTTTTTTTTTAAATTTTCCATTTTTCCCGAGATTCTTTTTAAAAATTTTC
AGTTTAAGTTTTATTTCTTTTTTAAATTTTTTTATTATTTTAATTTTGTTAATAATAATT
TTTAGTTCTGTTTCATTAGTATTTTTATTTTTTAAAAAATTTTTATTTTTTTAAAAATAAT
TTTAATATAAAAGTTTTTTTTTTTTTTTT---TTGAATTTAATTTTTGTTTTATTTTTT
TGT------------ATTTTAATAGTTTTTGTGTTTATTAGTAGAATTTTTGTTTTTTTA
TTTTATTTTTTAAATTTATTTTTATCTTTTAATTTTAATTTTTTTAATAAAATTAGTTCT
TTTGAGAGAGGATTTATAAGTCTTGGTAAGATTTTTAAATCTTTTAGTATTCATTTTTTT
TTAATTATAATTATATTTGTAATTTTTGATTTGGAAATTATTTTTATTTTGGGGTTTTTA
GTTTCTGATTTTGAGTGTTTTTTATTATTTTTTTTTTATTTTTTTTGTTTATATTATTAAGT
TTATATATTGAGTGATTTTTAGGTAAATTATTGTGGATTTTTTAA---------------
------------ATATTGTTTTTAAATAATTTTATTTTAATTTTTTTTTTTTTTTTATTTTA
TTATTATTTTATTTATTTTTTTTTTAATTTTTATTTAGGAGTTTTTGTTTTTTTTTTTAGT
GATTTTTTTATTATTTTGTTTTTTATAAGTTTATTAATTTTAGGGCTAGTTTTAATGAGT
GAATTTAATTTTAATATTAAGATTTTAAGGATTTTTTTAATTTTAATTAGATTTTTTTTT
TTTTTTAGTTCTAATATTTTTTTTTTTATATTTTTTTTTTTTGAAATATCAATTTTTCCAATT
TTAATTATAATTTTAGGTTTTGGGTATCAAATTGAAAAAATTAATTCTTCTTATTATATA
TTTTTTTATACTTTTTTTTGTTCTTTACCATTTTTTTTTTTTTTATTTTTAATATTGATTTA
ACTTTTAGTTTTTATTTTTTTGATTTGTTTTTCTCTTGAGAAATAGTTTTTTTTATTAAGA
ATTTTATTTTTAATAAAATTTCCTGTTTATTTCTTACATTTATGATTACCAAAAGCACAT
GTAGAAGCTTCAACTTCTGCTAGAATATTATTAGCTGGGTTATTATTAAAATTGGGTACT
GGAGGTTTTTTTCGTATTTTAGAGTGTTTAAATTTTTTTTTTTTTTAGGTTTTTATTTTTTA
ATTTCTTTTTTTGGGATAGTCTTAAGAAATTTTTATTGTATTTTTCAGAGTGATTTAAAA
TCTTTAGCAGCTTTTTCTTCTATTAATCATATAAGTTTTGTTTTATTTTTTATTAATTTTA
TTTAATATTTATAGTTTATGGAGAAGTGTGGTTATTATATTTTCTCATGGGTTAATTTCT
ACTTTAATATTTTTTTTTTTATTGGAAATTTTTATTATATTAGTTTAACACGTTTGATTTAT
TATTATTCTAGTGTTTTTTTTAAGTTCTTTAATTTTTTTGTTCTATAGTTGTTTTTACTTGA
TTATTTAATGGAGGAATCCCTTTTTCTTTATCATTTTTTTTCTGAATTTTCAGGATTTTTA
GTAATTTTTAATTTTAGTTATTATTTTTTTTTTTTTTTTAGATTTTTATATTTTTTTTATTTCT
TTTTATTATTGTTTATTTTTTTATTTCTTTAAATTTTATTGGTAAGAAAAAAAATAAATTTT
AATTTTTTTTTAGGTTATTATGGGAGATTTCTTTTACTTATAAATTGTAATTTTTTTTTTA
TTTTATTTTTTTTTATTAA---
---------------------------------------ATTATTGTGGATTTTTTAATT
TATATTTTAATTTTTTTTTATTATTATGTTTTTTTTTTTTTTTTTTTTATTTTTTTTTTTTTTTT
AATTTTAATTTTTTTTTTTTATTTTTTTTTGATTGAAAATTTTTTATTGTAAGATTAAATTTA
GGATTTTTAAAAAAAATTTTTTTTTTTTTTGTTATTAATTGTTGTATTGAGAGTTTTATTA
TTTTTAAATTTTTATTTAGAAAGAGAAACTTTTTTAGTTTATTTTATTTTTTATTTTGATA
ATTTTTATTGTTAGAATAATTTTTTTTAAATTTTAGGTTAAATTATTTTTTTAATAATTTTA
AGATGAGATTTATTAGGAATTTCTAGTTATTTTTTTAGTATTATTTTATAATAATTGAGAT
AGAAATATTGGTTCTATAAATGTTTCTTTAACTAATCGGATTGGAGATTATTTTATATTT
TTTTTTTTTTCTTTTTTTTTTGTTTTTAAATTTTAGTATAGATTTTAATATTTTTTTTGAAA
TGAATAATATTTTTTTTTTTTTAATTTTTATAGGTTTTACTAAAAGTGCTCAATTTCCTTTT
AGTAGTTGATTACCTAAAGCTATAAGAGCACCTACTCCTGTTAGTGCTTTAGTTCATAGT
AGAACTTTAGTTACTGCTGGTTTAATTTTGTTTATAAATTTTTTTTTTTTTTTTTTAAATTTT
AATTTTTTAATAGGTTTTTTATTATTTTTAGGTTTAATTACTATGTTTTTTTTCGAGATGT
ATATCTTTACAAGAAGAAGATTTGAAAAAGGTTGTTGCTTTAAGTACTTTATCACAAATA
GGTTTTGCAGTATTTATTTTAGGTCTTGGATTATTATATTTATCTTTAATTCATCTTATT
AGTCATGCTTTATTTAAGAGTTGTTTGTTTATTCAAATTGGTTTATTGATTTATTTTTCT
TTTGGTCAACAAGATGGTCGTTTTTTTAGTGGTTTAAATAATTTTATGTTACTTATTCAT
TTTCAAATTTTTATTACTTTATTTTGTTTATGTGGTTTATTTTTTAGGAGTGGTATAATA
AGTAAAGATTTAGTTTTGGAGAATTTTTTTTATTTAAATTGATTTTGATTGTTTTATTTA
TTTTTTTTTTTTTCAATTTTAATAACTTTTTTTTTATTCTTATCGTCTTTTTAAAGGTTTT
TTAAAATTTAAGAACATTTTGTTTTTAATTGGGGTAGATTTAAAGTTATTTTTATTTTCT
TTTTTTTTAATTTTATTTTCTTTGATTGGTATTTGATTGGTTTTAAAAAATTTATTTATT

TTTGTTAGAAGTTTTTTATTTTTAGATTTTTTTTTTTTGTATTTTTTTTTATTTTTTTTT
TTTATTTTAAAGTTTTAAATATTTTTTTTTTATTTATTATAATAAAATTTAGCTTTATG
GTAGATTATTTTGCTAAAATTTTTTCTTTTTTTATTTTTAATTTTAAATTTAATGATTAT
TTTTTAAATAAATTTTTATTTTTAAATCTAAGTTTTTTAAAAATTTTAAATTTAAATTTT
TTAAATTTTAATTTAAATTTTTTTTTTTTTAAATTTGATTTTTGTATATTTTTTCTTTTTT
ATTTAA------ATTTTTTATTTTAATTTAAAATATCATTTTTACAAGATGAAGATAAAA
AAATTTTTATTTATTAGGTTTTATCTTTTTTTTATTTTTTTAATTTGTTTATTTAGTTTT
TTTTCTTATGATCCTTTTAAAAGTTGTCTATTAATGATTTTTAGTTTATTATTTTTTTCT
TTTTTTTATAGTTTAAGATTTAATATTTGATTTAGTTATTTTATTTGTTTATTATTTTTA
AGAGGTATTTTTGTTATTTTAATTTATTTTTCAAGTTTAAGTTATTTTTATTATTTTAAT
TTTAATAAGGTTTTATTTTTATTATTTTTTTTTTTTTTTTTAGTTTATTTTTTTTTTTT
AAAAATTATTTTTTTTTTTCTTATTTATTTTTTTTTTTTTTATTATTTTATTTTTTTATTA
TTTTTAATAATTTTGGTTTATTTTTTGATGTTTTTAAGTTTTTATTTAAATTTTAGTGGA
GCAATACGTAAGTTTTAA---------------------ATTTTTTTTAAATTTTTTGGT
TTATTATTTTTTTTTTTTTAAGTTTTATCGTTTTATTTTTATTTTAATTTCTTTTGAGTTT
TTAGTAATAGGTCTATTTTATTTTTTTTAGTATTAATTTTAGGGGTATATTTTTTTTTTTT
TTTTTAAGTTTTAGTGTGATTTCAAGTGTTTTAGGAATGTTAATAATAATTTATTTAGTA
AAAAATTTTGGATTTGATAAAGTTTATTTTTAA
>B_fraudulentus_sample10
ATTTTAAAATTTAAATTTCAAGAAGGTTATAAATATTGATTTGAAAGTTCAAATCATAAA
GATATTGGTATATTATATTTTATTTTTGGTTTTTGATCAGGGATATTAGGAACTAGTTTA
TCTATAGTTATTCGTTTTGAATTAGCTAAACCAGGTTATTTTTTAAGAAATGGACAATTA
TATAATAGTATAATTACTGCTCATGCTTTATTAATAATTTTTTTTATAGTTATACCTTCA
ATAGTAGGAGGTTTTGGTAATTGAATATTACCTCTTATACTAGGTTCTCCTGATATAAGT
TTTCCACGTTTAAATAATCTTAGATTTTGGTTATTACCAACTTCCTTATTATTATTATTA
GACTCATGTTTTGTAGATACTGGTGCTGGAACTAGTTGAACTGTTTATCCTCCTTTAAGA
ACTTTAGGGCATCCTGGTGGAAGAGTAGATTTAGCTATTTTTAGATTACATTGTGCTGGT
GCAAGATCAATTTTAGGAGGTATTAATTTTATATGTACTACTAAAAATCTTCGTAGAAGT
TCTATTTCTTTAGAACATATAAGATTATTTGTTTGAACTATTTTTGTTACTGTATTTTTA
TTAGTTCTTTCTTTACCAGTATTAGCAGGTGCAATTACTATATTATTAACTGATCGAAAT
ATTAATACTTCTTTTTTTGATCCT------------------------------------

--GTAATTGCTGTT
CCTACTGGAGTAAAAGTTTTTAGTTGGTTGGCTACTTTATTTGGTTCAATTATGATTTTT
CAACCTTTATTATTATGAGTTTTAGGTTTTATTTTTTTTATTTACTATTGGTGGTTTAACT
GGGGTTATTCTTTCTAATTCTAGATTAGATATTATTTTACATGATACTTACTATGTAGTA
AGACATTTTCATTATGTTTTAAGATTAGGTGCAGTTTTTGGTATTTTTACTGGAGTTGTT
TTATGATGAAATTTAATTATAGGAGTTATTTATAATAAAATATTATTTATTACTATTTTT
ATTTTAATATTTTTTTGGGGTAAATTTAACTTTTTTTCCTTTACATTTTGCAGGTTTACAA
GGTTATCCTCGTAAATATTTAGATTATCCTGATATTTACTCTTTATGAAATATTATTTCT
TCTTTTGGTAGAATATTAAGAGTATTTTCTTTATTTTTATTTATTTTTTTAGTTATTGAT
TCTTTTAATAATTTTAAATTGTTTATTTCAGATTATTTTTTAAATTATAGACCTGAAAAT
AT--
---ATATTTAATTATTTTCAAGGTTATAATCTTAATTTTTCAAGAAGAATTTTTAGTTTT
TATATAGATTGATTTCATAGGTTTAATTGTAGTTTATTATTAGGAGTGTTATTATTTGTA
AGTTTATTATTTTTTTTTTTAATTTTTAATAATATTTATTTTAAAAGTAGAAAAAATTGAG
TATCAATTTGGAGAATTACTTTGTAGAATTTTTCCTACTTTAATTTTGTTGTTTCAAATA
GTGCCTTCTTTAAGATTACTTTATTATTATGGTTTAATAAGAATTGATTCTAATTTAACT
GTTAAAGTAGTTGGTCATCAATGGTATTGAAGATATGACTATAGTGATTTTCAAAATTTA
GAATTTGATTCTTATATAAAGTCTATTGACATATTAGAATTAGGAGAGTCTCGTTTATTA
GATGTTGATAATCGTTGTATTTTACCTTGTGATCTTAATATTCGTTTTTGTGTAACTTCT
GCTGATGTTATTCATGCTTGAACTTTATCTAATTTATATTTTAAATTAGATGCTATAAGT
GGTGTATTAAGAATTTTAAATTTTAACTTTCCTTTGGTTGGTTTATTTTATGGGCAATGC
TCTGAAATTTGTGGTGCGAATCATAGTTTTATACCTATTGTAGTTGAAGTTACTTTATTT
GATTTATTTAAATCTTGATGTTTATTAAGTTAG---------------------------

------------------TTTCTTTGAGGAAAGGATATTTCTTTTGAAGGATTGACTGGT
AGTCATAATTTTTTTGTTATAGATGGTTTTAAATTTGGTGTTATTTTATTTATTTTTAGT
GAATTTATATTTTTTTTTAGAATTTTTTGAACTTTTTTTGATGCTTCTTTAGTCCCTAGA
CATGATTTAGGTGAAATATGAAGTCCTTATGGTGTTAATTTAGTAAATCCTTTTGGAGTA
CCATTATTAAATACTATTATTTTGTTAAGTAGAGGTGTATCTGTTACTTGAGCACATTAT
AATTTATTAAGAAATAAAGATAGTTTAGTAAGTTTATTATTAACAATTTTTTTAGCTTTT
TATTTTATATTAATTCAACTAATAGAGTATAAAGAAGCAGGGTTTTCTATTTCTGATGGT
GTTTATGGAAGAATTTTTTATTTGTCTACTGGTTTTCATGGTTTTCATGTATTTTGTGGA
GGTTTATTTTTATGTTTTAATTTATATCGTCTTATTCTTTCTCATTTTACTTTTAATCAT
CATTTAGGTTTAGAATTTGCTATTATTTATTGACATTTTGTAGATGTTGTTTGATTATTT
TTATTTGTTTTTGTTTATTGATGA---------------------------------ATT
TTTTTATTTGTTTTTATTTTACAATATATTTTTTTTTTTTAATTTAAATTTTGTTTTTATT

TTATTTAAAAATTTTTTTTCTATTTTAAATGAAGTTTTTAGATATTCTAAAAATTTATTT
TTAAGAAGATTTATTTCTATTTTTGTCTTTTTATTACTTTTAACTTTATGTTTTGGTGGT
TATTTTAGTTATTCTTTTAGACCATGTGGAATAATTGAATTTACTTTAGTTTTTTCTATT
TTAACTTGATTAAGAACTTTTTTAAGATTTTTAAATAGTGAAAAAGTGTCTATTTATTTT
AGTAAAGGTGGAGATAGTTTTTTAAAGACTTTAAGTATATTAATTGTTGAAATTATTAGA
GAATTTTCTCGTCCTATTGCTTTAACAGTACGTTTAACAGTAAATATTATAGTAGGACAT
ATAATTAGTTTATCTGTTTATCTTTTAGTTGAA---AGTTTAGGTTATAAATTTTCTTTT
ATTATTATTTTTGCTATTTTAATAGAATGTTTTGTTTTTTTTATTCAAAGTTATATTTTT
TCACGTTTAATTTTTTTATATCTTAATGAATAT---------------------------

---------------------------------GCTTTTATAGGTTATGTTTTAGTTTGA
GCACAAATAAGTTTTTGGGCTTCTGTGGTAATTACAAGTTTATTGAGAGTAATCCCAATT
TGAGGAAATTTAATTGTTTCTTGAATTTGAAGTGGTTTTAGAGTTTCTGGAGCAACTTTA
AAATTTTTTTTTGTTTTACATTTTTTGTTACCTTGATTTTTTATTGTTTTAGTTTTAGTA
CATTTAATTCTTTTACATGATTTTGGAAGAACTTCTAAATTGAGTACTTTAACAGATTTA
GAAAAAGTTAATTTTTATAATTATTTCTGGTGAAAAGATGGTTATAATATTTTTATTTGA
ATTTTATTTTTTTTTTTTTGTTTATTTTTTCCTTTTAATTTAGGTGATCCAGAAATATTT
ATTGAGGCTGATCCTATAATAAGACCAGTTCATATTGTGCCAGAATGATATTTTTTATTT
GCTTATGCTATTTTACGTGCTATTCCTAATAAAGTTTTAGGTGTGATTTTTTTATTATTT
AGAATTTTATATTTTTTTTTTTTTGTTTTAAAATTAAATTTTTATTCTATTTTAAAAAAT
TTTAATTATTTTTTAGTAAATTTTTTTATTTTTTTTAGAATTTTTTTAAGTTGGTTAGGA
CATT--

------GTTAAGTTAATATCTAAAGAACAAATTTTAGTAAAAAATTCTTCTGATTTATAT
TTTTTATTTGTACCAGCTGTTTTTTTTTATTTTTATATTTTTAGAGTGATTAAGTTTACCA
TTTTTTTTTTTTTTTTATAAATTTTCAATTTTCTTTTCTTTTTTTTATTATGTTTAGTGGGG
TTTTCTGTTTATTTTACTATTATTAGGGGTCTTATAAGAAATTCTAAATTTTCTTTTTTA
GGAGCTATTCGTTCTAGTAGTCAAAGAGTGTCTTTTGAAATTGCATTTTCAATTTTTTTT
TTAATTTTTATATTTTTTGTTAATGGTTTTAATTTTGTTAAAATTTTTAATTTTTTTAGGA
TTTTTTATTTTTTACCCATTTTTAATTTTAGTATTATCTGAACTTAATCGTGCACCATTT
GATTTTTTTGAGGGTGAAAGAGAACTTGTAAGAGGTTTTAATACTGAACATTCTAGTGTT
AGTTTATTTTTCTTTTTTTAGGTGAATACGGAGTATTAATTTTTTTTTAGAATTTTAATA
AGTTTGTTATTTTTTAATTTTAGATATATTTTTTGTTTTTTATATTTTACTTTACTTTTA
TTAATTCGTAGAGCTTACCCACGTTTTCGTTATGATTTATTAATAAATTTATTTTGAAAA
ATTATTTTACCTTTGTCTTTA---------------------------------------

------------------------------CTTCTTTTTTTGATCCTATTTTTTTTTTTA
AGAAAAATTAATTTGAATGAAGAACCAAATTTATATTATTTTGTTGTTCAAGAGTTTTTG
GGTTTAATATTTATTTTTTTTTTTTTTTTAAATTTACAATGATTAATTTTATTATTAAAA
GTTGGGGTAGCACCTTTTGTATTTTGAGGTTTTTATTTGTTAGAAAATTTAAATAGGTTT
ATGATTTTTTTGATTTTTAACATTTCAAAAATTACCTTTTATTCCTATGTTAAAGGAA---
----

---ATTAATATTATTTTAATTTTTTTTTTTTATTTTAATCAGTATTTTTTTTGTTGTTGTT
TTTTATTTTTTAAATATAATTTTGTCTTTTAAATTTTTATATGTAAATAAAGTAAGTTCT
TTTGAAAGAGGATTTTTAAGTTTAGGAAAATTATTTAAATCTTTTAGACTTCATTTTTTT
TTAATTATAATTTTATTTGTTATTTTTGATTTGGAAGTAGTTTATGTTTTAGGTATTTTA
ATAAGTGATAGAGAAGTAATTTTTATTTTTATATTAATTTTTTTTTTTTATATTGTTTAGT
TTATATTTAGAGTGATTTTTAGGAAAATTAATTTGGGTTTTTTAA---------------

------------------------TTAAGATTTTTTTTAGTTATAGTAAGATTTTTTTTT
TTTTTTAGTATTAATTTTTTTTTATATTTATTTATTTTTTGAGTTTTCTTTATTTCCTATC
ATAGTAATAATTTTAGGTTATGGTTATCAGATTGAAAAAATTAATTCTTCTTATTATTTA
TTATTTTTTACTTTAATTTTTTCTATACCATTTTTTTTTTTTTTCTTTAATTTAGATTTA
AATATTAATTTAATATTTTTTAATTTATTTTTTAATTGAGAAATTATTTTAATTTTATCT
TTAATATTTATGTTAAAATTTCCAGTTTATTTTTTGCATTTTTGATTACCTAAAGCTCAT

```
GTAGAAGCACCTACTTCTGCTAGAATATTATTAGCTGGTTTATTACTTAAATTAGGAACA
GGTGGATTTTTACGTTTAATAAAAATTTTTGTTTTTTTTAATCTTAATTTTTATTTATTA
CTATCTTTTATTGGGATGATTTTTAGAAGTTTTATTTGTATTTTTCAAAGTGATTTAAAA
TCTTTAGCAGCTTATTCTTCTATTAATCATATAAGTTTA---------------------
------------------------------------------------------------
------------------------------------------------------------
------------------------------------------------------------
------------------------------------------------------------
------------------------------------------------------------
------------------------------------------------------------
------------------------------------------------------------
------------------------------------------------------------
------------------------------------------------------------
------------------------------------------------------------
------------------------------------------------------------
------------------------------------------------------------
------------------------------------------------------------
------------------------------------------------------------
------------------------------------------------------------
------------------------------------------------------------
------------------------------------------------------------
------------------------------------------------------------
------------------------------------------------------------
------------------------------------------------------------
------------------------------------------------------------
------------------------------------------------------------
------------------------------------------------------------
---------TTGTTTAAAAGTTTTTTATTTATTCAAGTAGGATTTTTTATTTATTTTTCT
TTTGGTCAACAAGATGGACGTTTGTATAGTGGTAATCAATATTTTATTTTTATTGTGAAG
TGAAATTTATTTTTAACTTTATTTTGTTTATGTGGTTTGTTTTTTAGAAGTGGTTTAGTA
AGAAAAGATATAATTTTAGAATTTTTTTTTTTTTCTAATTTTTATTTTTTGTTAGTGATA
ATATTTTTTATTTGTGTTTTATTAACTTTTTATTATTCTTTACGTTTATTTATAGGTTTA
GTAAAGTTATCTTTTAATTATTTTAAGATT------------------------------
------------------------------------------------------------
------------------------------------------------------------
------------------------------------------------------------
------------------------------------------------------------
------------------------------------------------------------
------------------------------------------------------------
------------------------------------------------------------
------------------------------------------------------------
------------------------------------------------------------
------------------------------------------------------------
------------------------------------------------------------
------------------------------------------------------------
------------------------------------------------------------
------------------------------------------------------------
------------------------------------------------------------
------------------------------------------------------------
------------------------------------------------------------
------------------------------------------------------------
---------------------------------
>B_fradulentus_CD4140
ATTTTAAAATTTAAATTTCAAGAAGGTTATAAATATTGATTTGAAAGTTCAAATCATAAA
GATATTGGTATATTATATTTTATTTTTGGTTTTTGATCAGGAATATTAGGAACTAGTTTA
TCTATAGTTATTCGTTTTGAATTAGCTAAACCAGGTTATTTTTTAAGAAATGGACAATTA
TATAATAGTATAATTACTGCTCATGCTTTATTAATAATTTTTTTTATAGTTATACCTTCA
ATAGTAGGAGGTTTTGGTAATTGAATATTACCTCTTATATTAGGTTCTCCTGATATAAGT
TTTCCACGTTTAAATAATCTTAGATTTTGGTTATTACCAACTTCATTATTATTATTATTA
GATTCATGTTTTGTAGATACTGGTGCTGGAACTAGTTGAACTGTCTATCCTCCTTTAAGA
ACTTTAGGGCATCCTGGTGGAAGAGTAGATTTAGCTATTTTTAGATTACATTGTGCTGGT
GCAAGATCAATTTTAGGAGGTATCAACTTTATATGTACTACTAAAAATCTTCGTAGAAGT
TCTATTTCTTTAGAACATATAAGATTATTTGTTTGAACTATTTTTGTTACTGTATTTTTA
TTAGTTCTTTCTTTACCAGTGTTAGCAGGTGCAATTACTATATTATTAACTGATCGAAAT
ATTAATACTTCTTTTTTTGATCCTAGAATAGGAGGTAACCCTTTAATTTATCAACATTTA
TTTTGATTTTTTGGTCATCCTGAAGTCTATATTTTGATTTTACCTGCTTTTGGTATTGTA
AGTCAAAGTACTCTTTATTTAACTGGTAAAAAAGAGGTTTTTGGTTCTTTAGGTATAGTA
TACGCAATTTTAAGAATTGGTCTTATTGGTTGTGTAGTTTGAGCTCATCATATATATACA
GTTGGTATAGATTTGGATTCTCGTGCTTACTTTACTGCTGCTACTATAGTAATTGCTGTT
CCTACTGGAGTAAAAGTTTTTAGATGGTTAGCTACTTTATTTGGTTCAATTATGATTTTT
CAACCTTTATTATTATGAGTTTTAGGTTTTATTTTTTTATTTACTATTGGTGGTTTAACT
```

GGAGTTATTCTTTCTAATTCTAGTTTAGATATTATTTTACATGATACTTATTATGTAGTA
AGACATTTTCATTATGTTTTAAGATTAGGTGCAGTTTTTGGTATTTTCACTGGAGTTGTT
TTATGATGAAATTTAATTATAGGAGTTATTTATAATAAAATATTATTTATTACTATTTTT
ATTTTAATATTTTTTGGGGTAAATTTAACTTTTTTTCCTTTACATTTTGCAGGTTTACAA
GGTTATCCTCGTAAATATTTAGATTATCCTGATATTTATTCTTTATGAAATATTATTTCT
TCTTTTGGTAGAATATTAAGAGTATTTTCTCTATTTTTATTTATTTTTTTAGTTATTGAT
TCTTTTAATAATTTTAAATTGTTTATTTCAGATTATTTTTTAAATTATAGACCTGAAAAT
ATTTTATCTGGTTATATTTTTAATCATAGTTACCAATCAGAAATATTTTTTATTGTTAAA
TAAATATTTAATTATTTTCAAGGTTATAATCTTAATTTTTCAAGAAGAATTTTTAGTTTT
TATATAGATTGATTTCATAGATTTAATTGTAGTTTATTATTAGGAGTATTATTATTTGTA
AGTTTATTATTTTTTTTTTTAATTTTTAATAATATTTATTTTAAAAGTAGAAAAAATTGAG
TATCAATTTGGAGAATTACTTTGTAGAATTTTTCCAACTTTAATTTTGTTGTTTCAAATA
GTACCTTCCTTAAGATTACTTTATTATTATGGTTTAATAAGAATCGATTCTAATCTAACT
GTTAAAGTAGTTGGTCATCAATGATATTGAAGATATGATTATAGTGATTTTCAAAATTTA
GAATTTGATTCTTATATAAAGTCTATTGACATATTAGAATTAGGAGAGTCTCGTTTATTA
GATGTTGATAATCGTTGTGTTTTACCTTGTGATTTTAATATTCGTTTTTGTGTAACTTCT
GCTGATGTTATTCATGCTTGAACTTTATCTAATTTATATTTTAAATTAGATGCTATAAGT
GGTGTATTAAGAATTTTAAATTTTAATTTTCCTTTGGTTGGTTTATTTTATGGACAATGT
TCTGAAATTTGTGGTGCGAATCATAGTTTTATGCCTATTGTAGTTGAGGTTACTTTGTTT
GATTTATTTAAATCTTGATGTTTATTAAGTTAGTTTTTTTACATTTTTTTTCATAATTTT
CATATTTTAACTTTATCTAGTTACCCTTATCAAGTTTTTTTAAGAACTTTTGGTTTAACT
TCTTCTTTTGTAATTTTTTTAAAATTTGGTATTTATTATGGAGTATTTATTAGTTTATTA
ATTTTATTTTATATTTCTTTTCTTTGAGGAAAGGATATTTCTTTTGAAGGATTGTCTGGT
AGTCATAATTTTTTTGTTATAGACGGTTTTAAATTTGGTGTTATTTTGTTTATTTTTAGT
GAATTTATATTTTTTTTTAGAATTTTTTGAACTTTTTTTGATGCTTCTTTAGTTCCTAGA
CATGACTTAGGTGAAATATGAAGTCCTTATGGTGTTAATTTAGTAAATCCTTTTGGAGTA
CCATTATTAAATACTATTATTTTATTAAGTAGAGGTGTGTCTGTTACTTGAGCACATTAT
AATTTATTAAGAAATAAAGATAGTTTAGTAAGTTTATTATTAACAATTTTTTTAGCTTTT
TATTTTATGTTAATTCAATTAATAGAATATAAAGAAGCAGGATTTTCTATTTCTGATGGT
GTTTATGGAAGAATTTTTTATTTGTCTACTGGTTTTCATGGTTTTCATGTATTTTGTGGA
GGTTTATTTTTATGTTTTAATTTATATCGTCTTATTCTTTCTCATTTTACTTTTAATCAT
CATTTAGGTTTAGAATTTGCTATTATTTATTGACATTTTGTAGATGTTGTTTGATTATTT
TTATTTGTTTTTGTTTATTGATGATCATTTTA-------------------------ATT
TTTTTATTTGTTTTTATTTTACAATATATTTTTTTTTTTTAATTTAAATTTTGTTTTTATT
TTATTTAAAAATTTTTTTTCTATTTTAAATGAAGTTTTTAGATATTCTAAAAATTTATTT
TTAAGAAGATTTATTTCTATTTTTGTATTTTTATTACTTTTAACTTTATGTTTTGGTGGT
TATTTTAGTTATTCTTTTAGACCATGTGGAATAATTGAATTTACTTTAGTTTTTTCTATT
TTAACTTGATTAAGAACTTTTTTAAGATTTTTAAATAGTGAAAAAGTGTCTATTTATTTT
AGTAAAGGTGGAGATAGTTTTTTAAAGACTTTAAGTATATTAATTGTTGAAATTATTAGA
GAATTTTCTCGTCCTATTGCTTTAACAGTACGTTTAACAGTAAATATTATAGTAGGACAT
ATAATTAGTTTATCTGTTTATCTTTTAGTTGAA---AGTTTAGGTTATAAATTTTCTTTT
ATTATTATTTTTGCTATTTTAATAGAATGTTTTGTTTTTTTTATTCAAAGTTATATTTTT
TCTCGTTTAATTTTTTTATATCTTAATGAATATATTAAATTTTTGTTAGATTTTACAAAA
CAGTTATTAGTTAATTTACCTACTAGAAAAAGATTAAGTTTAAATTGAAATTTTGGTAGT
CAGTTAGCTATAATTTTAGGTTTTCAAATTATTAGAGGTACTTTTTTAGTTTTTTTATTAT
TCTTCTGATAGAATTATAGCTTTTAATAGAGTTCAGTATATTATGATTGAATCTAATTTT
GGTTGACTTTTTCGTATTTTTCATTTTAATGGGGCAAGATTATTTTTTGTTTTTTTTATAT
TTACATTTTTTTAAAGGTTTATTTTTTAATAGTTATCGTTTAAAAAAAGTTTGGGTAAGT
GGTTTATTGATTTTTTTATTTATTATGATAGAAGCTTTTATAGGTTATGTTTTAGTTTGA
GCACAAATAAGTTTTTGGGCTTCTGTGGTAATTACAAGTTTATTGAGAGTAATTCCAATT
TGAGGAAATTTAATTGTTTCTTGAATTTGAAGTGGTTTTAGAGTTTCTGGAGCAACTTTA
AAATTTTTTTTTGTTTTACATTTTTTGTTACCTTGATTTTTTATTGTTTTAGTTTTGGTT
CATTTAATTCTTTTACATGACTTTGGAAGAACTTCTAAATTAAGTACTTTAACAGATTTA
GAAAAAGTTAATTTTTATAATTATTTTTGATGAAAAGATGGTTATAATATTTTTATTTGA
ATTTTATTTTTTTTTTTTTGTTTATTTTTTCCTTTTAATTTAGGTGATCCAGAAATATTT
ATTGAGGCTGATTCTATAATAAGACCAGTACATATTGTTCCAGAATGATATTTTTTATTT
GCTTATGCTATTTTACGTGCTATTCCTAATAAAGTTTTAGGTGTGATTTTTTTATTATTT
AGAATTTTATATTTTTTTTTTTTTGTTTTAAAATTAAATTTTTATTCTATTTTAAAAAAT
TTTAATTATTTTTTAGTAAATTTTTTTATTTTTTTTTAGAATTTTTTTAAGTTGGTTAGGA
CAGTGTTTAGTTGAGATTCCTTTTTTAATATTAAGTGGTTTATTTTCTTTTTTATATTTT
TTTTTTATTTTTATATTAATTTTTAATTATTATTTTAGTTTATTTTTTATTTAAATATGTA
AATATTATAAATTA-TAGTTCTTAATTATTTTAAATTTTATTTTATCTTTTCTTATGATT
TTATTTATTATTTTAAGAATTGCTTTTATTACTTTATACGAGCGTCATTTATTAGGTTTA
AGTCAAAATCGTCTTGGTCCAAATAAAGTTTTATTTTTAGGTGTTTTACAAGCTGGTTTA
GATGGTGTTAAGTTAATATCTAAAGAACAAATTTTAGTAAAAAATTCTTCTGATTTATAT
TTTTTATTTGTTCCAGCTGTTTCTTTTATTTTTATATTTTTAGAGTGATTAAGTTTACCA
TTTTTTTTTTTTTTTATAAATTTTCAATTTTCTTTTCTTTTTTTTATTATGTTTAGTAGGG
TTTTCTGTTTATTTTACTATTATTAGTGGTCTTATAAGAAATTCTAAATTTTCTTTTTTA
GGAGCTATTCGTTCTAGTAGTCAAAGAGTGTCTTTTGAAATTGCATTTTCAATTTTTTTT
TTAATTTTTATATTTTTTGTTAATGGTTTTAATTTTGTTAAAATTTTTAATTTTTTAGGA
TTTTTTATTTTTTATCCATTTTTAATTTTAGTATTATCTGAACTTAATCGTGCACCATTT

GATTTTTCTGAGGGTGAAAGAGAACTTGTAAGAGGTTTTAATACTGAACATTCTAGTGTT
AGTTTTATTTTTCTTTTTTTAGGTGAGTATGGCGTATTAATTTTTTTTAGAGTTTTAATA
AGTTTGTTATTTTTTAATTTTAGATATGTTTTTTGTTTTTTATATTTTACTTTACTTTTA
TTAATTCGTAGAGCTTATCCACGTTTTCGTTATGATTTATTAATAAATTTATTTTGAAAA
ATTATTTTACCTTTGTCTTTATTTATATTTTTTATTTATTGATTAATTTTATTTGCAACT
CTTTCATTTTTATTTTTTGTTTTTATTTATTTTATTTTTTTTTTTTTTATGTTTAAATTTT
TCAAATTTTATTTTATGATGAAGAATTTTTTTGTTAATAAGGATTTCATTTTTTTTTTTA
AGAAAAATTAATTTAAATGAAGAACCAAATTTATATTATTTTGTTGTTCAAGAGTTTTTA
GGTTTGATATTTATTTTTTTTTTTTTTTTAAATTTACAATGATTGATTTTACTATTAAAA
GTTGGAGTAGCACCTTTTGTATTTTGAGGTTTTTATTTGTTAGAAAATTTAAATAGGTTT
ATGATTTTTTGATTTTTAACATTTCAAAAATTACCTTTTATTCCTATATTAAAGGAATT-
--TTTAAGAAATTTTTTTTTTATATTTTTTATTGGAGTTATTTTAATTTATTTTATATTT
TTTATTATTAAAAAAAAATAAGTTTTTTATTTTTTTAAATTCTGTAGAATCTTTTAATTGA
GTTTTAATTAATTTATTTTTTAGTTATATAAATTTTTTTTATTTATTTATTTTTTTATTTT
TTTTCATTTGTTTTTTTAATTTTTTATTTTTATTTTTTTAGAT---AGTAATTTTAGTTTA
GTTTTAATTTTATTTATATTAAATTTTCCTTTTTCTTTAAGATTTTTTTTAAAGTTTTTT
AGTTTATCATATTTTAGTTTTCTAAATTTTTTTTTCTTTAATTATTTTATTTTTTAATAATT
TTA---

---ATTAATATTATTTTAATTTTTTTTTTTTTATTTTAATTAGTATTTTTTTTTGTTGTTGTT
TTTTATTTTTTAAATATAATTTTATCTTTTAAATTTTTATATGTAAACAAAGTAAGTTCT
TTTGAAAGAGGATTTTTAAGTTTAGGAAAATTATTTAAATCTTTTAGACTTCATTTTTTT
TTAATTATAATTTTATTTGTTATTTTTGATTTGGAAGTAGTTTATGTTTTAGGTATTTTA
ATAAGTGATAGAGAAGTAAATTTTATTTTTATATTAATTTTTTTTTTTTTATATTGTTTAGT
TTATATTTAGAGTGATTTCTAGGAAAATTAATTTGGGTTTTTTAAAAAGTTTTTTTATTT
TTGTTGTTTAGTCTAATTTTATTTTTTGATTTGAAATTAAGATTTTTGTTATTATTATTA
GTTGTGTTGTACACCAAAAAATTTTTTTATTTATTCAAGTGCTATTTTAGAAAATTTAAAT
ATTTTTTTATTTATTTTGGTTTTAATAAGTTTATTTATTTTGAGAAATATTTTTTTGAGT
GAACTTAATTTTAATTTAAAAATTTTAAGATTTTTTTTAGTTATAATAAGATTTTTTTTT
TTTTTTAGTATTAATTTTTTTTTATATTTATTTATTTTTTTGAGTTTTCTTTATTTCCTATT
ATAGTAATAATTTTAGGTTATGGTTATCAAATTGAAAAAATTAATTCTTCCTATTATTTA
TTATTTTTTACTTTAATTTTTTCTATACCATTTTTTTTTTTTTTTTTTTAATTTAGATTTA
AATATTAATTTAATATTTTTTAATTTATTTTTTTAATTGAGAAATTATTTTAATTTTATCT
TTAATATTTATGTTAAAATTTCCAGTTTATTTTTTTGCATTTTTGATTACCTAAAGCTCAT
GTAGAAGCACCTACTTCTGCTAGAATATTATTAGCTGGTTTATTACTTAAATTAGGAACA
GGTGGATTTTTACGTTTAATAAAAATTTTTGTTTTTTTTAATCTTAATTTTTATTTATTA
TTATCTTTTATTGGGATAATTTTTAGAAGTTTTATTTGTATTTTTCAAAGTGATTTAAAA
TCTTTAGCAGCTTATTCTTCTATTAATCATATAAGTTTAGTTTTATTAAGTTTAATTTTA
GTTAATTCTTTTAGAATTATTAATAGTGTTTTAGTTATACTTTCACATGGTTTTATTTCA
GTTTTGATATTTTATTATATTGGTGAATTTTATCATTTTAGTCAAACTCGTCTTGTTTAT
TATTATAGTAGTTTGTTTTCTTTTTCTGTAAGATTTGCGGTTTTTATTACTTTTGTTTGA
CTTTATAATAGAGGAGTTCCTTTATCTTTAACTTTTTTTGCAGAATTTAATATTTTTTTA
TCTGTTTTAAACTTTAACATTTTTTATTGATTTTTATTATTTTTATATTTTTTTTTAACT
TTTTATTATTGTTTATTTTTTATTTCTTTAAATTTTTTAGG-------------------

---------------------GAAGTAAATTTTATTTTTATATTAATTTTTTTTTTTTATA
TTGTTTAGTTTATATTTAGAGTGATTTCTAGGAAAATTAATTTGGGTTTTTTAATTTAAT
TTTTTATTTTTTTTTAGTATTTATTTTTTTTTTTTATTTTTTTTTTTATGATAATTTTTTTAAAA
GAATTTTTTCTTATATTTTTTTTTTTATTAATTGAAGATTTTTTAATTTGTTTTTTAATTTT
TTTTTAATAAAGATTTTATTTTTTATTTTATTAATATTGGTAGTTGTCAATGTTTTAATA
TTTATAAATTTTTATTTAGAGGGAGAATTGTATTTTAGTTATTTTTTTAATAATTTTAGTA
GTTTTTATTTTTAGAATAATTATACTTAATTTTAGTTTAAATTATTTATTATTAATGTTG
AGATGAGATGTTTTAGGTATTTCAAGTTATTTTTTAGTATTATTTTATAATAATTGAGAT
AGAAATATTGGTTCTATAAATGTTTCTTTAACTAATCGTTTAGGAGATTTTTTTTATATTT
TTTTTTTTTTCTTTTTTTTTTATATTTTAATTATGTTTTGGATTTTAATTTGTATTTAAAT
TTTATTTTTTTATTTTTTATTAATTGTAATAGGTTTTACTAAGAGTGCTCAATTTCCTTTT
AGAAGATGATTGCCAAAGGCAATAAGTGCTCCTACTCCTGTAAGAGCATTAGTTCATAGA
AGAACTTTAGTAACGGCTGGTTTAATTTTATTTTTTTAATTTTAAATTTTTATTAATGAAT
TTATATTTTTTAAAATTTCTTTTTTTTATTTGGTTTATTTACTATGATATTTTCAAGTTTA
ATAGCTTTGTTAGAAGAAGATTTAAAAAAAGTAGTAGCTTTAAGAACTTTATCTCAGATA
GGTTTTAGAGTTTTAATTTTAGGTTTAAGACTTTATTATTATAGTTTAATTCATTTAATT
AGGCATGCTTTGTTTAAAAGTTTTTTATTTATTCAAGTAGGATTTTTTTATTTATTTTTCT
TTTGGTCAACAAGATGGACGTTTGTATAGTGGTAATCAATATTTTATTTTTTATTGTGAAG
TGGAATTTATTTTTAACTTTATTTTGTTTATGTGGTTTGTTTTTTAGAAGTGGTTTAGTA
AGAAAAGATATAATTTTAGAATTTTTTTTTTTTTTCTAATTTTTATTTTTTTATTAGTGATG
ATATTTTTTGTTTGTGTTTTATTAACTTTTTATTATTCTTTACGTTTATTTATGGGTTTA
GTAAAGTTATCTTTTAATTATTTTAAGATTAGATTGTTAAATTGAATAATGTATGTTAGT
TTTTTATTATTTTTTTTTTTCTTTGGTTGGTATTTTTTTTTTTAAGTAATAATTTTTTATTA
TTTCCTAGTTTAATTTTATTTGAGGATTTTTTTAGTCCTATTTTTTTTTTTTAGTTATTTTT
TTTATTTTTTTTTTTAAATTTTATTTTTAAGTTTGTAATATTTAAATATAGATTTATAGTT
GATTATTATGCTAAGATTTTTTCTTTTTTTTTAAAAAATTTTAAATTTATAGATTTATTT

```
TTTAATAAGAATTTATTATTATTTTTTTTTTTTTTTTAAAAATATTTTAATAAAATTTATA
TTGTTAAATATTAATTATTGATTTTTTGTAGTTTTAGTAATATATTTTTTCTTATTTTTT
TAAAAG---ATTCTTTATTTTAATTTAAAATTTTATTTTTACAAGATAAAGATTATAGAA
TTTATATTAGTTTTAAATTTTTTTATTTATTTTGTAATAATGATTTGTTTTTTAAGTTTT
TTAAGTTTTGATCCTTTTAAAAGTTGTTTATTATTAATTTTTAGTTTACTTATTATAACT
TTAATATTTAGATTTAGAAATTATTTATGAATTAGTTATTATATTTGTTTGTTATTTTTA
AGTGGTGTATTTGTTATTTTAATTTATTTTTCTAGTTTAAGAAGATTTTTTTATTTTTTT
AGAAATATTATTTTTATTTTTTTAATTTTTTTTTTAAGTTTTTATATTTATTTTATTTTT
AAAATTTATTTTTATGATTTAATTTTTTTTTATTTTGTTTTTTATATATTTTTTTTTTTT
TTTTTTGTAGTGTGTTTGTTTTATTTTTTATTTTTTGTAAGT------------------
----------------------------------TTTTAATTTTTTTTATTATTTTTAGT
TTTTTATTTTTTATTTTAAAATTTTATCGTTTTATTTTTATTCTTTTGAGTTTTGAATTT
TTTGTTATTGGTTTATTTTATTATTATTCTTTTATTTTATCAGGATTTATATTTTTTTTT
TTTTTATCTTTTAGAGTTTTATCAAGAGTTACTGGGATATTAATGATAATTTATTTAGTA
AAATCTTTTGGTAAAGAAAAAGTTATTTTTTAA
>B_arthuri_sample8
------------------------------------------------------------
------------------------------------------------------------
------------------------------------------------------------
---------------------------------------------------ATACCATCT
ATAGTTGGTGGTTTTGGAAATTGAATAGTACCTTTAATATTAGGTTCTCCTGATATAAGA
TTTCCTCGTTTGAATAATTTAAGTTTTTGATTATTACCTATTTCTTTAATATTATTACTG
GACTCATGTTTTGTAGATATAGGTGCAGGCACAAGGTGAACTGTTTATCCACCTTTAAGA
ACTTTAGGTCACCCTGGTAGAAGAGTTGATTTAGCAATTTTTAGGTTACATTGTGCAGGT
GTAAGTTCTATTTTAGGTGGTATTAATTTTATATGTACTACTAAAAATTTACGTAGAAGA
TCTATTTCTCTTGAACATATGGGTTTATTTGTTTGAACAATTTTTATTACAGTTTTTTTA
TTAGTTCTTTCTTTACCAGTTTTAGCCGGTGCTATTACTATATTATTAACTGATCGTAAT
ATTAATACTTCATTTTTTGATCCTAGTATAGGAGGTAACCCTTTAATT------------
------------------------------------------------------------
------------------------------------------------------------
------------------------------------------------------------
------------------------------------------------------------
------------------------------------------------------------
------------------------------------------------------------
------------------------------------------------------------
------------------------------------------------------------
------------------------------------------------------------
------------------------------------------------------------
------------------------------------------------------------
------------------------------------------------------------
------------------------------------------------------------
------------------------------------------------------------
---ATTTATAGTTTTTTTCAAGGTTATAATTTATTATTTCAAAATAGAATTTTTTCTTAT
TATATAGATTGATTTCATAGTTTTAATTGTAGTTTAATGTTAGGAGTATTAATTTTTGTA
AGTTTACTTTTTATTTTTTTAATTATTAATATTTTTTTTTTTAAAAGTAAAAAAATTGAA
TATCAATTTGGTGAATTAATATGTAGTTTATTTCCTACTTTAATTTTATTATTCCAAATA
ATTCCATCTTTAAGTTTATTATATTTTTATGGTTTAATAAATATTAATTCTAATCTTTCA
GTAAAAGTTATTGGGCATCAATGATATTGAAGTTATGATTATAGTGATTTTGAAGATTTA
GAATTTGATTCTTACATAAAATCTTTAGATATTTTAGATTTAGGTGAAAGACGTTTATTA
GATGTTGATAATCGTTGTATTTTACCTTTTAATACTAATATTCGTTTTTGTATTACTTCT
GCTGATGTTATCCATGCTTGAGCTTTATCTAGTTTATCGATTAAATTAGATGCTATAAGA
GGTATTTTAAGAATTTTAAATTATAATTTTTCTTCTATTGGGTTATTTTATGGACAATGT
TCAGAAATTTGTGGTGCCAATCATAGTTTTATACCTATTGTTCTTGAAGTAACTTTATTT
GATTTATTTAATAATTGATGTTTATTATTTTAG---------------------------
------------------------------------------------------------
------------------------------------------------------------
------------------------------------------------------------
------------------------------------------------------------
------------------------------------------------------------
------------------------------------------------------------
------------------------------------------------------------
------------------------------------------------------------
------------------------------------------------------------
------------------------------------------------------------
------------------------------------------------------------
------------------------------------------------------------
---------------------------------ATTAATAATGTTTATTTTTTAGATATT
TTTTTATTTATATTTTTATTACAATATATTTTTTTTTTTAAAACAAAATTTATTATTTTCT
TTTTTTAAAATAATTTTAAATAATTTAAATGATGTTTTTAGTTATAGAAAAAATTTAATT
TTAAGAAGTTTTATTTCTCTTTTTAGATTTTTATTTTTATTAATTTGTTGTTATGGTGGT
TATTTTAGTTATTCTTTTAGTCCATGTGGAATAATTGAATTTACTTTAAATTTAGCTTTA
```

ATTTCTTGGTTAAGTACTTTTCTTTGTTTTATTACTAGAGAAAAAGTTTCTATTTATTTT
AGAAAAGAAGGTGACTCTTTTTTAAAAACTTTAAGAATATTAATTGTTGAAATTGTGAGT
GAGTTTTCTCGTCCTATTGCTTTAACAGTTCGTTTAACTGTTAATATTATAGTTGGTCAT
ATAATTAGTATATCAATTTATATATTAGTAGAA---AGTTTAGGTTTAAAATATTTTTTT
TTTACTATTTTTGCTATTTTAATAGAATGTTTTGTTTTTATTATTCAAAGTTATATTTTT
TCTCGTTTAATTTTTCTTTATTTAAATGAATAA---------------------------

---------------------------------ACAAGGTTGTTAAGTGTAATTCCTTTT
TTAGGTCCTAAAATTGTTGTATGAATTTGAAGGGGTTTTAGTGTTTCAGGTGCTACTTTA
AAATTTTTTTTTGTTTTACATTTTATTTTACCTTGATTATTTTTAATTTTAATTTTATTA
CATTTAATTTTTTTACATGATTTTGGTAGAACTTCTAAAATAGTTTGTCATGGTGATTAT
GATAAAATTAATTTTTTTTTCTTTTTATTGATGAAAGGATGGGTATAATATAATTATTTGA
TTTTTATTTTTTATTTTTAGTTTTTTTTATCCATTTATTTTAGGTGATCCAGAAATATTT
ATTGAAGCTGACCCTATAATGAGACCTGTTCATATTATTCCTGAATGATATTTTTTATTT
GCTTATGCTATTTTACGTGCTATTCCAAATAAAATTTTAGGTGTAATTTTTCTTTTATTA
AGAATTTTAATTTTTTATATTTTTATTTTTAAATTAAATTATTTAAGTTTATTGAAAAAA
AATAATTTATTTTTAGTTAATTATTTTATTTTTTTTAGAATTATTTTAAGTTGATTAGGT
CAATGTATAGTTGAACCGCCTTATTTATTTTTAAGTGCTTTATTTTCATTTTTATATTTT
ATA---

TTTTTAATTATTCCAGGAGTTTCTTTTATTTTTATAATTTTGGAATGGTTAAGTTTACCT
TTTTTATTTTTTTTTTTAAATTTTCAATTTTCTTTATTTTTTTTTAATATGTTTAATTGGG
TTTTCTGTTTATTTTACTATTATTAGAGGTTTAATAAGAAATTCTAAATATTCTTTTATT
GGTGCCATTCGTTCTAGTAGTCAAAGTGTATCTTTTGAAATTATATTTTCTATTTATATG
ATTAGTTTTATATTATTTTTAAATTCTTTAAATTTATTTTTTTTTATTTAATTTTAGTTTA
ATTATTTTATTTTATCCTTTTTTATTAATAATTTTAGCTGAATTAAATCGTGCTCCATTT
GATTTTTCTGAGGGTGAAAGAGAATTAGTTAGAGGTTTTAATACTGAACATTCTAGTGTA
AGTTTTATTTTTTTATTTTTAGGAGAATATGGAGTTTTAATTTTTTTTTAGAATTTTAAGA
AGTTTAATTTTTTTTAATTTTTCTTTTTTTTTTTATTTTTTTTTTATTTTTCTTTACTTTTA
TTAATTCGTAGATCTTACCCTCGTTATCGTTATGATAAATTAATAAATATATTTTGAAAA
ATAATTTTACCTTTAATTATTTATGTTTTTTTATTTTAT---------------------

--GTAATTTT
TTTTTTAGTTTTAATATTTTTTTTTTTATATTTAAGTTTTGAATTATCTTTATTTCCTATT
ATTATTATAATTTTAGGATATGGTTATCAAATTGAAAAAATTAATTCTTCTTACTATTTA
TTTTTTTTTACTATTATATGTTCTATACCATTTTTTTTCTTTATTTTTAATTTAAATCTT
AATAATTTTTTTATTTATTTTTTATATTTTTTTTCTTGAGAAATAGAATTAATTTTAACT
TTACTTTTTTTAGTTAAATTTCCAGTATATTTTTTACATTTTTGATTACCTAAAGCTCAT
GTAGAAGCACCTACTACTGCGAGAATATTATTGGCAGGGTTACTTTTAAAATTAGGAACT
GGAGGATTTTTACGTTTAATTAATTCTTTAAATTTTCAATTTTTAGGGTTGTATTTTTTA
ATTGCTTTTTTAGGTATAATTTTAAGAAATTTTAATTGTTTAATTCAAAGTGATTTAAAA

TCTCTTTCAGCTTTTTCTTCTATTAATCATATAAGATTTGTTTTA---------------

---------------------------------------AATTATTTTATAATAATTTTA
AGATGAGATATTTTAGGTATTTCAAGATATTTTTTAGTTTTATTTTATAATAATTGAGAC
AGAAATATTGGTTCTATAAATGTTTCTTTAACTAATCGTTTAGGGGATTTTTTTATATTT
TATTTTTTTTCTTTTTTTTTTTTTTATTAATTTAAATTTTAATTTTTTAAATTTTTATTAT
TTTTTATTAATTTTTATAATTTTAGTTATAGGTTTTACTAAAAGTGCACAATATCCTTTT
AGTAGTTGGTTACCTAAAGCTATGAGTGCACCTACTCCTGTTAGAGCTTTAGTTCATAGA
AGAACTTTGGTTACTGCTGGTTTAATTTTATTTTTGAATTTTGAT---------------

>B_tuscia_sample24A
---------------------------------AAATGATTTGAAAGTTCAAATCATAAA
GATATTGGAATATTGTATTTTATTTTTGGTTTTTGATCTGGTATATTAGGTACTAGTTTA
TCTATAATTATTCGTTTTGAGTTAGCTAAACCTGGATATTTTTTAAATAATGGTCAATTG
TATAATTCAGTTATTACTGCTCATGCTTTATTAATAATTTTTTTTATAGTTATACCTTCT
ATAGTGGGGGGTTTTGGTAATTGATTATTACCTCTTATATTGGGTTGTCCTGATATAAGA
TTTCCTCGTTTAAATAATTTAAGTTTTTGATTACTTCCTACTTCTTTAATATTGTTACTA
GATGCTTGTTTTGTTGATTCAGGAAGAGGTACTAGTTGAACAGTGTACCCACCCCTAAGT
ACTTTGGGGCATCCTGGTAGAAGTGTTGATTTAGCTATTTTTAGTCTTCATTGTGCAGGG
GCAAGTTCTATTTTAGGTGGTATTAATTTTATATGTACTACTAAGAATTTACGTAGAAGT
TCTATTTCTTTAGAACATATGAGTTTATTTGTTTGAACTATTTTTGTTACTGTTTTTTTA
TTAGTT------------------------------------TTATCA------------
------------TTTTTTGATCCTAGAATAGGAGGAAATCCTTTAATTTATCAACATTTA
TTTTGATTTTTTGGTCATCCAGAGGTGTATATTTTAATTTTACCTGCTTTTGGTATTGTA
AGTCAAAGTACTTTATTTTTAACAGGTAAAAAAGAAGTTTTTGGTTCATTAGGAATAGTT
TATGCTATTTTAAGAATTGGATTGATTGGTTGTGTTGTTTGGGCCCATCATATATATACT
GTAGGTATAGATTTAGATTCTCGTGCTTATTTTACTGCTGCCACAATAGTAATTGCTGTA
CCTACTGGTGTTAAAGTTTTTAGT------------------------------------

--ATTTTAATTTTTCAAATA
ATTCCTTCGTTAAGCTTACTCTATTATTATGGTTTAATAAACTTAGACTCTAATTTAACT
GTAAAAGTTATTGGTCATCAATGATACTGAAGATATGACTATAGTGATTTTGAAGAGTTA
GAATTTGATTCGTATATGAAATCTATTGATTCTTTAGATTTGGGGGATCTGCGTTTATTA
GATGTAGATAATCGTTGTGTCTTACCTGTTGATTTAAGTATTCGTTTTTGTATTACTTCA
GCAGATGTTATTCATGCTTGATCTTTATCAAGTTTGTCTGTTAAATTAGATGCTATAAGA
GGTGTTCTTAGTATTTTAAATTATAATTTTCCAGTGGTTGGTCTTTTTTTTGGGCAATGC
TCAGAAATTTGTGGAGCTAACCATAGTTTTATGCCTATTGTAGTTGAAGTTACTTTATTT
GATTTATTTAAAAATTGATGTTTTTTAGTATAA---------------------------

--------------------GGATGGTTTTAAGTTTGGGGTGTTATTATTTATTTTTAGT
GAGTTTATATTTTTTTTTGGTATTTTTTGAACTTTTTTTGATGCTTCTTTAGTCCCTACT
CATGATTTGGGTGAAATATGAAGTCCGTATGGTTTAAGTTTGGTTAATCCTTTTGGAGTG
CCTTTATTAAATACTATTATTCTTTTAAGAAGAGGAGTAACTGTTACCTGAGCACATTAT
AGTCTGTTAAGAAATAAAGATAGTTTGAGTAGTCTTATTATTACTTGTTTTTTAGCAGTT
TATTTTATTTTAATTCAGTTAATAGAATATTCTGAAGCTAGTTTTTCTATTTCTGATGGA
ATTTATGGTAGAATTTTTTTTT--------------------------------------

-------------------------------TAGTGGAATTTACTTTATTATTTGCTTTG
ATTTCTTGGTTGAGAACTTTTTTAAGTTTTATTTCTAGAGAAAAAATTTTTATTTATTTT
AGAAAGGGGGGTGATTCATTTTTAAAAACTCTTAGAATATTAGTAGTTGAAATTGTAAGA
GAATTTTCTCGTCCAATTGCTTTGACAGTTCGTCTTACTGTTAATATCATAGTTGGTCAT
ATGATTAGATTATCTTTATTTTTATTAATCGAA---AATTTAGGTTATTTTTATTTCTTT
TTTTCTATTTTTGCTATTTTAATAGAATGTTTTGTTTTTATTATTCAAAGTTATATTTTT
TCTCGTTTAATTTTTTTATATTTAAATGAATA-----------TAGTGTTTTTTATTAAA
GGTTTATTAGTTAATTTACCGTCTAGTAAGTGTTTAAGTTTAAATTGAAATTTTGGTAGT
CAATTGGCTTTTGTTCTTTTATTCCAATTAGTAACAGGGGTGTTTTTAGTTTTTTATTAT
TCAGCTGATAGTTTATTGGCATTTAATAGTGTACAGTATATTATAATGGAGGTGAATTAT
GGTTGGTTATTTCGTATTTTTCATTTTAATGGAGCAAGATTTTTTTTTATTTTTTTTATAT
TTACATTTTTTTAAAGGATTATTTTTTTTTTAGTTATCGTTTAATTAAAGTTTGAATAAGA
GGAATTTTTATTTTTTTAATAGTTATAATGGAGGCTTTTATGGGTTATGTTTTAGTTTGA
GCACAGATAAGATTTTGAGCTTCTGTTGTTATTACTAGGTTGTTAAGTGTGATTCCTATT
TGAGGGCCTTTAATTGTTTCTTGGATTTGGAGTGGATTTAGGGTTTCAGGGGCAACATTG
AAATTTTTTTTTGTTTTACATTTTTTGTTGCCTTGGTTTTTTTTAGTTTTGGTTTTAGTT
CATTTAATTTTTTTGCATAGTACAGGTAGAAGTTCTAAAATTTTATGTCATGGAGATTAT
GATAAAATTAATTTTTATTATTTTTATTGATGAAAAGACGGGTATAATTTTATTTTGTGA
TTATTTTTTTTAGTTTTTAGTTTTTTTTTTCCATTTTTATTAGGGGACCCTGAAATATTT
ATTGAAGCAGATCCTATGTTGAGTCCTGTGCATATTATTCCTGAGTGATATTTTTTATTT
GCTTATGCAATTTTACGTGCTATTCCTAATAAGATTTTGGGGGTGATTTTTTTATTGTTG
AGAATTTTAATTTTCTATTCGTTTATTTTAAAGTTAAATTATTTAAGTTTGTTTAAGAAA
AGAAATTACTTATTTGTTTTTATTTTTTTTTTTATCAGAATTATTTTAAGTTGATTGGGT
CAATGTTTAGTGGAGCCTCCTTTTTTATTTTTAAGAGGGTTGGTGTCTTTGTTATATTTT
GTTTTAATTTTATTAATTTTATTTAATTAT------------------------------
---TAGAGTTGTTAATGTTA
TTATTTATTGTTTTAAGAATTGCTTTTGTAACTTTGTATGAGCGTCATTTGCTAGGTTTA
AGTCAAAATCGTTTAGGTCCCAATAAAGTTTTTTTTTTGGGAGTAGTACAAGCAGCATTG
GATGGTGTTAAATTACTTAGTAAAGAGCAGATTTTACCTGTTTTTTCTTCTGAAATTTAT
TTTTTATTAATTCCTGGTGTTTCTTTTATTTTTATATTTTTAGAATGAATAAGGCTGCCT
TTTATATTTTTTTTTATAAATTTTCAGTATTCTTTTTTATTTTTAATATGTTTAGTGGGA
TTTTCTGTTTATTTTACTATTATTAGTGGATTGATAAGTAACTCTAAATATTCTTTTTTA
GGTGCCATTCGTTCAAGTAGTCAGAGTGTGTCATTTGAAATTGCTTTTTCGATTTATATT
TTTAATTTTATATTATTTTTGAATAGGTTTTCTTTTTTTTTTTTTAATTAATTTAAGTTTA
TTTTTGATATTTTACCCTTTTTTATTAATAGTTTTGGCTGAATTGAATCGTGCTCCGTTT
GATTTTTCAGAAAGAGAGAGTGAATTAGTGAGTGGGTTTAATACGGAACATTCTAGGGTA
AGTTTTGTATTTTTATTTTTAGGGGAATATGGAGTTTTGATTTTTTTTAGTGTTTTAAGT
AGTTGTTTATTTTTTTATTTTTCTTTTATTTTTATTTTTTTTATACTTAAGGCTATTATTA

TTGATTCGTAGGGTGTACCCTCGTTTTCGTTATGATTTTTTAATAGAAGTTTTTTGAAAA
AAGATTTTGCCTCTTTCTTTATTTATGTTATTTTTTTATTTTTTTAATGATTTA-------
----------------------TGTTGTATTTGTTAATATTTTTTATAAGGCTAAGTTTT
AATAATTACATATATTGGTGAGTAATTTTTTTACTAATGAGCTTGAGTTTTTTTTTTTTA
AATAAATTTTATTTTAATGATTATAGCAGGGTTAATTATTTTATCATTCAAGAGAGGTTG
GGTTTGTTTTTTTTAGTTTTTATTGTGAGAGATCTGCAGTGACTTTTATTGATATGTAAA
ATAGGTGTCTCACCTTTTTTTTTTTGATTATTTTTAATTGTTGATAACTTGAGTAGTGTT
ATATTTTTTTGATTTTTAACTTTTCAAAAGTTACCTTTTATTCCTATTATTAAAGATTT-
--TTTTTTTAATTTTTACTATATTTTATTCTTAGGTTTATTTATAATTTATTTATTAATT
TTTTGTTTGAAAAAAGTTAAGTTTTTAATTTTTTTAAATTCCACAGAATCTTTTAGCTGA
ATTTTAATTAATTTATTTTTCAGGGGTTCAAATTTTTTGTTTTTATTTTTCTATTATTTG
TTAAGAATATTATTTTTAATAGAAATTTGAGGAGCACAT------AATATTTTAAATTTA
GACTTAGTATTATTTTTATTAAATTTTCCCTTTTTTCCTAGGTTTTTTATTAAATTTTTT
AGTCTAAGATTTTTATCTTTTAATAATTTTTTTTTGTTATTTTTTCTTTTCTTTATAGTT
TTGAGTAGATTTTCTTTAGTTTTTATTTTGTTTAAAAAAGTCATATTTTTAAACTTTCAA
TATTATAAAAACATTTTTTTTTTTTTTTTTTTTTTTGAATTTAAATTTTGTGTTATTTTTT
TGTAAAGTTTATGTTTTATTATTTATATTT---ATTTTAAGGTTTATTTTTGTTTTGTTA
TTTTTTTTTATTAATTTTATTTTATCTTTTAAGATTTTGAATTTTGTAAAAGTTAGTACC
TTTGAAAGAGGTTTTGTAAGGCTAGGAAAAGTTTTTAAGTCTTTTGGGATTCATTTTTTT
TTAATTATAATTATATTTGTGGTTTTTGATCTGGAGATTGTTTTTATTTTGGTTCTTTTA
ATTTCAGACATTGAGGGTTATTTTTTTTTTTTTTTTAGTTTTTCTTTTTATAATTTTAAGT
TTGTATTATGAGTGATTTTTAGGAAAATTGATTTGAGGTATTTAG---------------

----------------------TGTTATTTTTTTTTTTTTTGTTAGCCATATTATTTTTT
AATTTTAATATTTTTTTTATTATTTTAGATTGGAAATTATTTTTTTTGGGGTTAGATTTA
TATTTTTATAAAAAAATTTTTTTTATTTTGTTGGTTTTTGTAGTTATGAGAGTGTTATTA
TTTTTAAATTTTTATTTAGATTCTGAAAATTATTTGACTTATTTTGTAGTTATTTTAATA
ATTTTTGTTTTTAGAATAATTTTTTTTAAATTTTAGTATAAATTATTTTGTTATATTGCTA
AGTTGAGATTTGCTAGGTATCTCTAGATATTTTTTAGTATTATTCTATAATAATTGAGAT
AGGAATATTGGGTCTATAAATGTTTCTCTTACTAATCGTTTAGGTGATTATTTTATATTT
TTTTTTTTTCTTTTTTTTTATTTATTAATTATAGTTTTAGTTTTATGGTTTTTTTAAAG
TTTTTATTATTTTTTTTTTTTAGTATTAATAGGTTTTACTAAAAGAGCACAGTTTCCTTTT
AGAAGTTGACTTCCTAAAGCCATAAGTGCCCCTACTCCTGTTAGTGCGTTAGTTCATAGA
AGTACATTGGTAACTGCGGGGTTAGTTTTATTAATAAATTTTTATTTTTTAGTTAATTTT
TTTTATTTAATAGTGGTGTTGTTGTTATTTGGTTTGTTAACAATATTTTTTTCTAGTATT
ATGTCTTTACAAGAAGAAGATTTAAAAAAAGTAGTTGCTCTTAGTACTTTGTCACAAATA
GGTTTTGCTTGTTTAATTTTAGGTTTAGGTTTAAATTTTCTTTCATTATTTCATTTAATT
AGGCATGCTTTGTTTAAGAGTTGTTTATTTATTCAAATTGGAGTTTTAATTTTTTTTTCT
TTAGGACAACAGGATGGTCGTTTCTTTAGGGGTCTTAATAAATTTAGTAATTTAATTCAT
TTTCAAATTTTTATTACTTTGTTTTGTCTTTGTGGTTTATTTTTTAGTAGTGGTATAATG
ACTAAAGATTTAGTATTGGAATTTTTTTTTTATAAATAATTGGGGAGTATTTTTTTATTTT
TTTTTTTTTGTTCAATTTTATTA-------------------------------------


```
------------------------------------------------------------
------------------------------------------------------------
------------------------------------------------------------
------------------------------------------------------------
------------------------------------------------------------
------------------------------------------------------------
------------------------------------------------------------
------------------------------------ATTATATTTTTTTTATTTTTTGGT
TTATTATTTTTTTTTATAAAATTTTATCGTTTTATTTTTATTTTAATTTCTTTTGAATTT
TTGGTTATGGGTTTATTTTATAATTTGAGATTTAATTTTTCGGGGTTGTATTTTTTTTTT
TTTTTAAATTTTAGAGTTTTATCAAGAGTTATGGGAATGTTGATGATAATTTATTTATTA
AAAAATTTTGGTTCAGATAAAATTTTTTTTTGC
>B_fungivorus_sample11
------------------------------------------------------------
------------------------------------------------------------
------------------------------------------------------------
------------------------------------------------------------
------------------AATAGATTAGTACCTTTAATATTAGGTTCTCCAGATATAAGT
TTTCCTCGTTTAAATAATTTAAGATTTTGATTATTGCCAACATCTCTGATATTACTTTTA
GATGCTTGTTTTGTTGATATAGGTGCTGGTACTAGTTGAACTGTTTATCCACCTTTAAGT
ACTTTAGGTCATCCAGGTAGTAGAGTTGATTTAGCAATCTTTAGTCTTCATTGTGCAGGT
GTAAGTTCAATTTTAGGTGGTATTAATTTTATATGTACAACTAAAAATCTTCGTAGGAGT
TCTATTTCCTTAGAACATATAGGTTTATTTGTTTGAACAATTTTTATTACAGTTTTTTTA
TTAGTTTTATCTTTACCTGTATTAGCAGGTGCTATCACAATATTATTGACTGATCGTAAT
ATTAATACATCTTTTTTTGATCCTAGAATAGGAGGTAACCCTTTAATTTATCAACATTTA
TTTTGATTTTTTGGACATCCAGAAGTTTATATTCTAATTTTACCAGCGTTTGGTATTATT
AGACATAGAATTTTATATTTAACCGGTAAAAAGGAAGTTTTTGGATCTTTAGGTATAGTG
TATGCTATTTTAAGAATTGGTTTAATTGGCTGTGTAGTGTGGGCTCATCATATATATACT
GTCGGTATAGATTTAGATTCTCGTGCTTATTTTACTGCAGCTACAATAGTTATTGCAGTT
CCTACTGGAGTAAAAGTTTTTAGTTGATTGGCTACTTTATTTGGCTCTGTAATAAAGTTT
CAACCTTTATTACTTTGAGTTTTGGGGTTTATTTTTTTATTTACTATTGGTGGTTTAACT
GGTGTAATTTTATCTAATTCTAGTTTAGATATTATTTTGCATGATACTTATTATGTAGTA
AGTCATTTTCATTATGTTTTAAGTCTAGGTGCTGTATTTGGAATTTTTACAGGAGTTTCT
TTATGATGAACTTTTATTATAGGTTTAGTTTATAATAAAATTTTATTTAGAGCAGTTTTT
ATT---------------------------------------------------------
------------------------------------------------------------
------------------------------------------------------------
------------------------------------------------------------
------------------------------------------------------------
---------AGTTATTTTCAAGGTTATAATTTATTATTTCAGAATAGAATTTTTTCTTTT
TATATAGATTGATTTCATAGTTTTAATTGTAGTTTAATAATAGGTGTATTAATTTTTGTA
AGTTTATTATTTTTATTTTTAATTTTTAATTTAAATTTTTTTAAAAGTAAAAAAAATTGAG
TATCAGTTTGGTGAATTA------------------------------------------
------------------------------------------------------------
------------------------------------------------------------
------------------------------------------------------------
------------------------CCAGTTAATACTAATATTCGTTTTTGTATTACATCT
GCGGATGTTATTCATGCATGAGCTATTTCTAGATTATCAATTAAATTAGATGCTATAAGT
GGTATTTTAAGAATTTTAAATTATAATTTTCCTTCTGTTGGTTTATTTTATGGTCAATGT
TCAGAGATTTGTGGGGCCAATCATAGATTTATACCTATTGTTTTAGAAGTAACTTTATTT
AATTTATTTAATAATTGATGT------------------------------CATAATTTT
CATATTCTTACTCTTTCTAGTTTTCCTTATCAAGTATTTTTATGTGTTTCTGGGTTAACA
TCTTCTTTAGTAATTTTTTTTAAATATGGAATTTTAAGAGGAGTTTTTTTAAGTTTTTTA
ATTTTATTCTATGTTTCTTTTTTTATGATCTAAAGATATTTCTTTTGAAGGTTTAAGAGGT
TATCATAATTTTTATGTTATAGATGGGTTTAAATTTGGTGTGATTCTATTTATTTTTAGA
GAATTTATATTTTTTTTTTAGTATTTTTTTGAACATTTTTTGATGCTTCTTTAGTACCCTCT
CATGATTTAGGTGAAATGTGAAGACCCTATGGTTTTAAT---------------------
------------------------------------------------------------
------------------------------------------------------------
------------------------------------------------------------
------------------------------------------------------------
------------------------------------------CATTTTAATTTCAATCAT
CATTTAGGTTTGGAGTTTGCTATTATTTATTGACATTTTGTTGATGTTGTTTGGTTGTTT
TTATTTGTTTTTGTTTATTGATGATCA------ATTCATAATGTATATTTTTTAGATATT
TTTTTATTTATTTATTTATTACAATTTATTTTTTTTTTTAAATTCTAATTTATTATTTTTA
TTATTTAAAAATTTTTTGTTTATATTAAATGACATTTTTAGATATAGAAAAAATTTAATT
TTAAGAAGGTTTATTTCTGTTTTTTGTTTTTTATTTTTATTAACTTGTTGTTATGGAGGT
TATTTTAGTTATTCTTTTAGTCCTTGTGGTATAATTGAGTTTACTTTAAGTTTAGCCTTA
ATTTCATGGCTAAGAACTTTTTTATGTTTTATTTCCAGAGAAAAAGTTTCAATTTATTTT
AGAAAGGAAGGGGATTCTTTCTTAAAAACTTTAAGAATATTAGTAGTGGAGATTGTTAGA
GAATTCTCACGTCCTATTGCTTTAACAGTTCGTCTGACTGTTAATATTATAGTTGGGCAT
```

ATAATTAGAATGTCTATTTATATACTTGTTGAA---AGGTTAGGATTTAAATATTTTTTT
TTAACTTTATTTGCTATTATAATAGAATGTTTTGTTTTTATTATTCAGAGATATATTTTT
TCACGTTTAATTTTTCTTTATTTAAATGAGTAA---------------------------

---------------AGAATTGCTTTTATTACTTTATATGAACGTCATTTATTAAGTTTA
AGTCAAAATCGTTTGGGGCCTAATAAAGTTTCTTTTATGGGAGTATTACAGGCAGCTTTA
GATGGAGTTAAATTAATATCCAAAGAACAGATTTTACCTTTTAATTCTTCAGATTTATAT
TTTTTAATTATTCCAGGGGTATCTTTTATTTTTATAATCTTAGAATGAGTGAGATTACCT
TTTTATTTTTATTTTTTGAATTTTCAATTTTCTTTTTTTTTTTTTAATAAATTTAATTGGG
TTTTCAGTTTATTTTACTATTATTAGTGGGTTAATAAGAAATTCTAAATATTCTTTTATT
GGAGCAATTCGTTCTAGAAGTCAAAGAGTATCTTTTGAAATTATATT-------------

---ATT
CTTTCATTTTTAATTTTTGTTTTAATTTATTTATTAATTTTTTTTTTTTCTTTAATTTTT
AATAATTATATTCTTTGATGAAGAATTTTTTTAATTTTGAATATTAGTTTTTTTTTTATA
AATAAAATTTTTATAAATGATTTAAGAAACTTATATTATTTTATTATTCAAGAACTATTA
GGTATATTATTCTTATTTTTTTATTTTTATAAATTTTCAATGATTGATTATTATGTTAAAA
ATTGGTGTGTCACCTTTTTTTTTTTGAGTTTTTTTTATTTTGGAAAATTTAGTGAGTTTA
ATAATATTTTGGTTTTTGACTTTTCAAAAATTACCTTATTTACCTTTAGTAAAAGATTT-
--TTTTTTAAATTTCTTGTTTTTTTTTTATTTTAGGTGTTTTATTAATTTATTTTATAATT
TTTTTTATAAAGAAAATTAAATTTATAATTCTCTTAAATTCATTTGAATCTTTTAGATGA
ATTCTTATTAATTTGTATTTTAGAAATTTCAATTTTTTTATTTTAATTTTATTTTATTTG
ATAGTGTTTAATCATCCATCAATTGCCTATAAGGGAGTAAGT---TGTAGTAAAGTAAGA
GTAAAGGAATTTGTTCTTAGAAAAAAACTAA-----------------------------

------------------ATTTTGTCATTTAAATTTAATTTGCAATTTAAAATTTCTTCT
TTTGAAAGTGGTTTTGTTAGAATTGGTAAGATGTTTAAATCTTTAAGTTTACATTTTTTT
ATAATTTTAATTATATTTGTTGTTTTTGATTTGGAAATTATTTTTATTTTTGGGTTTTTA
ATAAGGGATATGGAAAGAATAAATATTTTTTTTGTAATTTTTTTTTTTTATAATTTTGAGT
TTATATTTAGAGTGATTTTTGGGTAAATTATTATGATTGTTATAT---------------

--ATTATTTT
TTTTTTAGTATTAGTATTTTTTTATTTATATTTAAGTTTTGAATTATCATTATTTCCTATT
CTAGCTATAATTTTAGGTTATGGTTATCAAATTGAAAAGATTAATGCATCTTATTACTTA
CTAATATTTACTGTAATTTTGTCTATACCTTTTTTTTTTTTTTATTTTTAATTTAGATTTA
AATAAAAATTTTTTAAATTTTTATTTTTTTTTTTCTTGAGAAATAGAATTGATTATGATT
TTAATATTTATAGTTAAATTTCCTGTTTATTTTCTACATTTTTGGTTACCTAAAGCGCAT
GTAGAAGCCCCTACAAGAGCTAGAATATTACTAGCAGGTTTATTATTAAAATTAGGAACA
GGAGGATTTTTACGTTTTTTAAATTCTATTAATTTTCATTTTTTAGGTCCAATAATTTTT
ATTTCATTTTTTGGAATAATTTTGAGAAATTTTAATTGTATAATTCAAAGTGATTTAAAA
TCTTTAGCAGCATTTTCTTCAATTAATCATATAAGATTTGTTTTATTAATTTTAGTATTA
TTAAATATTATAAGAGTTAAAAGAAGAGTAATTATTATATTATCTCATGGGTTTATCTCT
ACTCTTATATTTTATTATATTGGTGAATTTTATCATTTTTCTTTAACTCGTTTAATTTAT

TATTATTCAAGAATTTTTTTAAATTCTTTATTTTTTTGTTTAATGATTAGACTAACTTGA
TTATTTAATTCCGGTATCCCATTTTCTATTACATTTTATTCAGAGTTTATTTCTTTTTTA
GTAATTTTTAATTTT---

------------------------ATTGATTGAAAATTTTTTATTATAAATTTGAATTTT
TCATTTTTTAAAATAATATTTTTTTTTTTTGTTGTTAATTGTTGTGATTAGAATTTTTTTA
TTTTTAAATTTTTATTTATCTAATGAATTATATTTTATTTATTTTTTTTATGATTTTATTA
ATTTTTATTTCTAGAATAATTTTTTTAAACTTTAGAAATAATTATTTTATAATAATTATT
AGTTGGGATATATTAGGTATTTCTAGATATTTTTTAGTTTTATTTTATAATAATTGAGAT
AGTAATATTGGTTCAATAAATGTTTCTCTTACTAATCGTTTAGGAGATTTTTTTATATTT
TTTTATTTTAGT--

---------------------------AAAGTTGTGGCTTTAAGAACTTTATCTCAGATA
GGTTTTAGTGTAATAATTTTAGGTTTAAATTTAAATTATTTTTGTTTAATACATTTGATT
AGGCATGCTTTATTTAAAAGTTGTTTATTTATTCAATTAGGTTTAATTATTTATTTTATA
TATGGACAACAAGATAATCGCTTTTATAGAGGTTTAAAATATTTTTTTAATTTTATTAAA
TGACAATTTTTATTAACTTTGTTTTGTTTGTGTGGTTTATTTTTTAGTAGTGGTATAATA
AGAAAAGATATAGTTTTAGAATTTTTTTTTTTTTTTAATAAAAGTTTATTTTTAATATTA
TTTTTTTTTTTTAGGGTTTTAATAACTTTTTTTTATTCT---------------------

--ATAAAGATAAAA
AAGTTTATAATTTTTTATTTATTTTTTATAATAATTTTTATTATAAGTCTTTATAGATTT
TTTTCTTTTGATCCTTTTAAGAGTTGTTTAATAATAATTTTAATTTTATTAATTTTTTCT
TTTTATTTGAGATTAAATAAAAATATTTGATTAAGTTATTATATTTGTTTATTATTTTTA
AGAGGTATTTTTGTTATTTTAATTTATTTTTCTAGATTAAGAAGATTTTTTTTTTTTTAT
TTTAGTAAAATTTATATTTTTTTTTTTTTTATTTTTTTTTTTTTTATAGTTTTTATTTCAAA
TTAAATTTTAATTTAAATTTAGTTTTATATTTCATAAATTTTTTTATTTATTATTTTTATT
ATATTTGTTTTTATTTTGTTTTATTTTTTTATTTTTTTATTAGATTTAATTTAAATTTTCAA
GGAGCTTTACGTAAAATTTAA---------------------------------------

>Panagrolaimus_detritophagus_OZ076443
ATAAATAAGTTTAAATATTATAGTGGTATTTCTAATTGATTAGAAAGTTCTAATCATAAA
GATATTGGAACTTTATATTTTATTTTTGGTCTTTGGTCTGGTATGTTGGGTACTAGAATG
TCTATAATTATTCGTTTTGAATTAGCTAAACCTGGTTATTTTTTAGGTAACGGTCAAGTC
TATAATTCTATTTTAACTGCTCATGCTTTATTAATAATTTTTTTTATAGTTATACCTACA
ATGATTGGTGGTTTTGGTAATTGAATATTACCTTTAATATTGGGTTCTCCAGATATAAGT
TTTCCACGTTTGAATAATTTAAGTTTTTGATTATTGCCTACTTCAATTTTATTATTATTA
GATGCTAGTTTTGTTGATATGGGTTGTGGTACAAGTTGGACTGTATACCCTCCTTTAAGT
ACTATAGGTCATCCTGGTAGAAGAGTTGATTTAGCAATTTTTAGCTTGCATTGTGCTGGG
GCTAGTTCTATTTTAGGTGGTATTAATTTTATATGTACAACTAAAAATTTACGTAGAAGT
TCTATTTCTTTAGAACATATAAGTTTATTTGTTTGAACAGTTTTTGTAACAGTTTTTTTA
TTAGTTCTTTCTTTACCTGTTTTAGCTGGTGCTATTACTATATTATTAACTGATCGTAAT
ATTAATACTTCTTTTTTTGACCCAAGGGCTGGAGGTAACCCTTTAATTTATCAACATTTA
TTTTGGTTTTTTGGACATCCTGAAGTTTATATTTTAATTTTACCAGCTTTTGGTATTATT
AGTCAAAGGGCTATATATTTGACCGGTAAAAAAGAAGTTTTTGGTACTCTAGGTATAGTT
TATGCTATTTTAAGTATTGGTTTAATTGGTTGTGTTGTTTGAGCTCATCATATATATACT
GTTGGTATAGATTTAGATTCTCGTGCATATTTTACTGCTGCTACTATGGTTATTGCTGTT
CCTACAGGAGTAAAAGTTTTTAGATGATTAGCTACTTTATTTGGTACTAAGATAAATTTT
CAACCTTTATTATTGTGGGTTTTAGGTTTTATTTTTCTATTCACTATTGGAGGGATTACT
GGTGTTGTTTTATCTAATTCTAGATTGGATATTATTTTACATGATACTTATTACGTTGTA
GCTCATTTTCATTATGTTTTAAGTTTAGGAGCTGTTTTTGGTATTTTTACTGGAGTTTCT
TTATGGTGAAATTATGTTACAGGCCTAGTTTATGATAAAACTATAATAAGAGCAGTTTTT
ATTTTAATATTTGTTGGTGTAAATTTGACTTTTTTTCCTCTTCATTTTGCTGGTTTACAT
GGTTTTCCTCGTAAATATATTGATTATCCTGACACATATTCTGTATGAAATATTATTTCT
TCTTATGGTTCTATAATTAGAGTTTTTTCTTTATTTTTGTTTATTTATGTAATATTAGAA

```
TCTTTTATAAAATTTCGTCTTTTATTAGTTGATTACAATATTAATAGAGGTCCAGAGGGT
GCTTTAAGAAGTTACATTTTTGGTCATAGTTATCAAAGTGAGGTTTATTTTAATGTAAGT
AGAATTACAAATTATTTTAATGGTTACAATTTGAATTTTTCAGGAAGTTTATTTTCTAGT
TATTTGGATTGATTTCATAGTTTTAATTGTAGATTAATATTAGGTGTTTTAATTTTTGTT
AGTTTGATATTTTTATATTTAATATATGGTAATTTTTATATAAAAAGTAAAAAAAATTGAA
TATCAATTTGGTGAATTGTTGTGTAGAGTTTTTCCAACTATTATTTTATTAATTCAAATG
GTTCCATCTTTAAGTTTATTATATTATTATGGGTTAATAAATTTAGACAGTCAAATTACT
GTAAAAGTAACTGGTCATCAATGGTATTGAAGATATGAAATTTCTGATATCCCAGGACTA
GAATTTGATTCTTATATAAAATCTTTAGATATATTGGAATATGGAGAGCCTCGTTTATTA
GAGGTAGATAACCGTTGTGTAGTTCCTTGTGATACAAATCTTCGTTTTTGTATTTCTTCT
GCTGATGTTATTCATGCTTGGGCTCTTTCAACTATATCTGTTAAACTTGATGCTATAAGG
GGTGTTTTGAGTATTTTAAATTATAATTTTCCTTTAGTTGGAGTTTTTTATGGTCAGTGT
TCAGAAATTTGTGGTGCTAATCATAGTTTTATACCTATTGCTTTAGAAGTAACTCTTTTT
GATTCTTTTAAAAGTTGATGTTTAGCTATATTAAAAAAGTGTATTATATTTCATAATTTT
CATATTTTAACTTTATCTGTTTTCCCTTATTTAATTTTTATATGTTCTTTTGGCCTTACT
ACTTCTATTGTTTATTTTTTTAAGTTTGGCTTTTATTGAAGTTTATTGTTTTGTTTGTCT
GTTTTGTTTTATGTTAGTTATTGTTGGTCAAAAGATATTGTTATAGAGGGTTTGAGTGGT
TACCATAATTTTTTTGTTATAGATGGGTTTAAGTTTGGGGTAGTTTTATTTATTTTTAGA
GAGTTTATATTTTTTTTTAGAATTTTTTGAACTTTTTTTGATGCTGCTTTGGTCCCAGCT
CATGATTTAGGTGAAACTTGATCTCCTTTTGGTATAGTTTTAGTTAATCCTTTTGGTGTT
CCTTTGTTAAATACAATTATTTTATTAAGTAGAGGTGTAACTGTTACTTGAGCTCATTAC
AGTTTATTAAGAAATAAATCTTGTACTTCCGGTATATTTTTGACAGTTGTTTTAGCTATT
ATATTTACTGCTATTCAAGGTATAGAATATAAAGAAGCTGGATTTTCAATAGCTGATGGG
ATTTACGGTAGAGTTTTTTATTTGGCTACAGGTTTTCATGGTATACATGTTCTTTTTGGT
GGTTTGTTTTTATTTTTTAATTTAATTCGTCTTCTTTACTCTCATTTTAATTTTAGACAT
CATTTGGGTTTAGAATTTGCTATTTTATATTGACATTTTGTTGACGTTGTTTGGTTGTTT
TTATTTGTTTTTGTATA----------------ATTAATAATGTATATTTTTTAGATATT
TTTATATTTATTTTTGTTTTACAATTTATTTTTTATATAAAAGAGGGTTTATTAAATACT
TTAAATAAAAATTTTTTATCTAGTTTAGTAGGAGTTTTTAGTTATAGTAGAAACTTACCT
TTAAGCTCTTTTATTTCAGTTTTTACTTTTCTAATTTTATTAACTTGTTGTTTTGGAGGT
TATTTCTGTTATTCTTTTTGTCCTTGTGGTATAGTAGAATTTACTTTTGTTTATGCTATA
TTAGCTTGAATGAGGGCTTTTTTAACTTTTATTTCTAGAGAAAAGTTTTCGATTTATATA
AGAAAATCTGGAGATAAGTTTTTAAAAACTTTAAGTATATTAGCTGTTGAACTGGTAAGA
GAGTTTTCTCGTCCTATTGCTTTAACTGTTCGTTTAACAGTTAATATTATAGTTGGTCAT
TTAATTAGAATAATAATTTATCAGGCTTTAGAATTAAGTTTAGGAGATTTTTATATTTGA
ATTACTATTTTTGCTATTATAATGGAGTGTTTTGTATTTTTTTATTCAAAGTTATATTTTT
TCACGTTTAATTTTCTTATATATTAGAGAATAAATAAAACAATTATTATATTTTGTAAAT
TCTTTATTAATTTCTTTACCTAGAAGGAAAAGTTTAAGTATTAATTGAAATTTCGGTAGT
ATGTTAGGTATAGTTTTAGTATTCCAATTATTAACTGGTACTCTTTTAGCTATTTATTAT
TCTGCTGATAGAAGTATTGCTTTTAATAGAGTTCAATATATTATGTATGAAGTTAATTTT
GGATGAATTTTTCGTATTTTTCATTTTAATGGTGCAAGTTTATTTTTTTATTTTTTTTATAT
TTACATTTTTTTAAAGCTTTATTTTTTAGGAGTTATCGTTTAAAATTTGTTTGAGCCACA
GGTTTAACTATTTTTCTTTTAGTTATAATGGAAGCTTTTATAGGTTATGTTTTAGTTTGG
GCTCAAATAAGATTTTGGGCTTCTGTTGTTATTACTAGTTTGTTGAGGGTTATTCCTATT
TGAGGTCCTTCTCTAGTTGTTTGAATTTGGAGTGGATTTACTGTTTCAGGAGCCACATTA
AAATTTTTTTTTGTTTTACATTTTCTTGTACCTTGATTTATTTTAGTAGTGGTTTTGGTA
CATTTAATTTTTTTGCATGATACAGGTAGTACTTCTAAAGTGTTTTGTCATGGTGATTAT
GATAAAATTAGATTTTTTCCTTATTATTGAATTAAAGATTCTTACAATATTGTTTTTTGG
TTAATTTTTTTAGTATTTTCTTTAATTTTACCTTTTGATCTTGGTGATCCTGAAATGTTT
ATTGAGGCTGATCCTATAATAAGACCTGTCCATATTGTTCCTGAATGATATTTTTTATTT
GCTTATGCTATTCTTCGTGCTATCCCAAATAAAATTTTAGGTGTTTTGGCTTTGTTAATA
AGAATTGTTAGATTTTATTTTTTCATCTTAAATTCGAGGTACCATGCTGTTCTTGATAAA
TTAAATAAATTTTTGGTTTTTATTTTTATTTTTGTTTCTTTAATTTTAAGTTGATTAGGA
CAGTGTTTAGTTGAGCCCCCTTATGTTTTTTTAAGAGCTCTATTTTCTATTTTTTATTTT
TTATTAGTTTATATTTTGCTTTTTATTTATAATTTTATAAATTTTTTATTTAAATGTACA
CATATTATAAGGAA-------------ATTTTTAGATTAATTCATATTTTAATTATTTTT
ATTTTTATTTTACAAGCTATCGCTTTTATTACTCTTTATGAGCGTCATTTTTTAAGTATT
AGTCAGAATCGTTTAGGACCTACTAAAGTTAGTTTTGGTGGTATTTTACAAGCTTTTATG
GATGGTGTAAAATTGTTAAAAAAAGAACAAATTATACCTTTTCACTCTTCTTATTTTTTA
TTTATTTTTGTACCTGGAATTTCTTTCATTTTGATACTTTTAGAATGGTTTGTCCTACCT
TATAATTATTATTTTTTTGAATTTTGAATTTAGTTTAATATTTTTTCTTTGTTTGGTGGGG
TTTTCTGTTTACGGTACTTTAATTAGAGGTGTGGTAAGAAAATCTAAGTATTCCGTTATC
GGCTCTATTCGTGCTAGAAATCAAAGTGTATCTTTTGAAATTGCTTTTTCTATTTTTATT
TTTTGTATTATGTTCTATATTAATAAAGTTTGTTTTGAATCTTTTTTTAATTTAAATTTA
ATTTTTATTATTGTCCCTTTTATTTTAATGCTTTTAGCTGAATTAAACCGGGCTCCTTTT
GATTTCGCAGAGGGGGAGAGAGAGTTGGTTAGAGGTTTTAATGTAGAATATTCTAGAGTT
GCTTTTGTTTTATTATTTTTGAGAGAATACGGTGTTATAATTTTTTTTAGAGTTTTGTTT
TCTAGAATATTTATGGATTTTAGAATCGTTATTGTTTTTTTTACTTTTAGTTTATTTATT
GCTTTACGTAGATCTTACCCTCGTTATCGTTATGATTTTTTAATATCTTTTTTTTGATTT
AAACTTTTACCTTTATCTATTATTTTACTTTTTTTTTATTATACTATGAATTTT------
------ATTTATTTTTTTGGTTTATTAATATATATTTTTTTTTTGATAAATTTATATGTA
```

AGAAATTTAATTTTTTGATGGAGAGTTTTTTTAATTATAACTTTAATTTTTTTATTTTTA
GGTAAGTCTAAAAGTAATTTTTCTAGTATGATAAATTATTTTATTATTCAAGAATTTTTA
GGTTTAATATTTCTATTAATAGGTTTTATAATATTACAAGTTTTAATTTTATTTATTAAA
ATTGGTGTTTCACCTTTTCATTTTTGGGTTTATTCTATTATTTATAGTTTAGATGGTTTT
ATATTAGTTTGATTTTTAACTTTTCAAAAGTTACCTTTTATTTCTGTTTTAATTTATTAC
AATTTTAGATGTTTTTTTTTAATTTTAGTTTTAGGAATTTTATTTTGCTATATACAAGTT
TTTATATTAAAAAATTTTAAATTAATATTTTTAGTTTCTTCTACTGAAAGTTTTAGTTGA
ATTTTATTTGGTTTTTTAGGAGGTTTATTGAGTTTAGGTTTTATTTTTTTTTATTATTTA
ATAAATATATTAATTTTAATAAGATATTTAAACTTAAACGGTTCAAGTTTTTTAGGGTTA
GAAACAATTTTAGTCTTTCTTAATATTCCTTTAAGAGTTACTTTTATAGTTAAAATTTTT
ATATTGTTTTTATTAATAAATATTTTTGATTTTTTAGTTTTATTTTTTATTAATATTAATA
TTTTTGTCTTCGCTAGGTTTAATATTTTGGTTTAGTTCTTTTAGAATATTATTAAAAAAT
TCTTATAAGGATTTTTATTTAAATACTTATTTTATAATTTATTTTTTGTTTTTATTAATT
A-----------ATTTATTTATTTTTTTGTTTTTTAATTTCTCTTTTTTTAGTTATAGTT
TTTTATTTATTAAATTTTTTTTTGAGTTTAAAATTTTTTTCTGAAGTTAAAATATCTTCT
TTTGAAAGAGGTTTTGAAAGTATTGGAAAAAATTCATAGTTCTTTTAGAATTCATTTTTTT
ATTATTATATTAATATTTGTAATTTTTGATTTGGAAGTAGTTATATTAATAGGTTTTTTA
TTAAGAGATTTAAGATTTTTTTTAAATTTTTTATTAATTTTGGTTTTTGTTTTGGGGGGC
TTTTATATAGAGTGATTTTTTTGGTAAATTGGTTTGAATTATTTAAGCTGTTTTTATATAT
TTTTTTCCTGTTTTATATTTTATTTTAGATTTTAATATTTCTTATTTTTTTTTTATTAATT
TGATCTTTTTTATTTTTTTATTATATACTCTTGAAGAGGTTTATTTTTTTGTAGGGGACTCT
ATAATGTTTGTTTTACTTGTTTTAATATCTATTTTTATTATAGGTTTGGTTTTTATTAGT
GAGATAAATTTTAATATACTTTTTTTATCTTATATTTTAGTTATTGTAAGAATTTTTTTC
TTCTTTTCTTTTAATATTATTATGTTGTATATTTTTTTTGAACTTTCTATGTTTCCTATT
ATAATTATAATTTTAGGTTATGGTGCTCAGATTGAAAAAATTAATTCTTCTTATTATTTA
ATATTTTATGCTTCTTTTTGTTCTTTTCCTTTTTTGTTTATTTATATATATTATAATTTA
AACTTATCTTTTGTTTATTTTGATAAAATTCTTTCTTGAGAATTGGTTTTTTTTTTACTT
TTAGGATTTTTAATAAAGTTTCCTGTTTATTTTTTACATTTATGGTTACCTAAGGCTCAT
GTTGAGGCTCCTACTACTGCTAGAATGTTGTTGGCTGGTCTTTTATTAAAATTAGGTACA
GGTGGATTTTTACGTGTTTTAAAAACTTTTTCATTTTTACATTCAAATTTTTGATTTTTT
ATTTCATTTTTAGGAATAATTTTAGGTTCTTTTTCTTGTATTTTTCAAAGTGATTCTAAA
TCTTTGGCAGCTTATTCTTCAATTACTCATATAGGCTTTTTATTAATATGTTTGTTATTT
ATTTCTATAGAGGGTAAACTCTCTAGTTTAATATTAATATTGTCTCATGGTTATACTTCT
ACTTTAATATTTTATTTTATTGGTGAACTTTATCATATTTCAGGGAGCCGTATAGTTTAT
TATATCAACAGACTTTTCAATATTAGTTTTATAGTAGGTATTTTTTTTTGTTTTAACTTTT
TTAAGTAATGCTGGCGTTCCGCCTTCATTAAGTTTTTTTTCTGAGTTTATTTCAATTAGT
TTTGGTTTAGGTTTAATAAATTTTATATTTTTAATCTTATTTTTATATTTTTTTTTTGCT
TTTTATTATTCAATTTATTTTATTACTAATGTCGTTATAGGAAAAAAATTTCTTTAATATT
CTTAATTTTAGCGGTTATTACTCAATATTTTTTATTTTTATAATATTTAATTTTTTTTGA
TTTAGTGTTTTTTTTTGA--
--ATTATA
ATTTCTATTTTTTTGATATTTTTTTTTTTTATTGTTAATAGTTTTTTTATTTTATTTTTTA
AGAGGTTTATTAAATTTTTATATTTTAGAATGATTATTTTTTTCTTTAAAATTTAATTTT
TATTTTAATAGTTTGGTTTTTTCTTTCATTTTATTATTTGTAAGACTTAGTGTTTTAATT
TTTAGAAGTTATTATTTATCTGGAGAATTAAATTTTTCTTATTATTTTATAGTTTTAGTT
ATTTTTATTTTTAGAATATTTAGATTAAATTTTAGATATAGGGCTGTTACAATATTATTA
AGTTGAGATTTGTTAGGTATTTCAAGATTTTTTTTAGTTCTTTTTTATAATAATTGAGAT
AGTTGTAGAGGATCCATAAATACAGTATTAACTAATCGTTTGGGTGATTTTTTTTTTGTTT
GTTTTTTTTAGAAGTTCTATTTTTGGGAGTATAAGTTTTTTAAGATATAGGATATTTTTT
TGGGGTTCTTTTTTATTTTTAATTTTAACTGCTTTTACAAAAAGGGCCCAATTTCCTTTC
AGTGGTTGATTACCTAAGGCTATAAGGGCTCCTACTCCTGTTAGTTCTTTAGTTCATAGA
AGTACTTTGGTAACTGCCGGTTTACTTTTAATTTTTAATTTTAGTTTAATATTAATAAAC
AATTATATTATATCTTATATTTTTTTTACTGGGTTATTTACTACTTTATTTTCAAGTTTA
AGGGCTGTTGTAGAAGAAGATTTGAAAAAAGTAGTGGCTTTAAGAACTTTATCTCAAATA
GGTTTTTCTATAATAACTTTTGGTTTAGGAATTTCTTTTTTATCATTAATACATTTGTTA
AGTCATGCTTTGTTTAAAAGCTGTTTATTTATACAGGTTGGTTATTTGATTCATTGTTCT
TTCGGTCAACAAGATGGACGTTTTTATGGTTTTTTAAAGTTTATCCCTAATTTTATCCAA
ATTCAATTATTAGTAACTTTATTTTGTTTATGTGGTTTGTTTTTTTACTAGAGGTTTGGTG
AGGAAGGATTTAATTTTAGAATTTTTTTTTTTTTAATAGAAATTATTTTTTTAGTATGTTTA
ATTTTTTTTATTACTGTTTATATAACTTTTTTTTTATAGTTATCGTTTGTGAAAAGGTTTA
TTTATAAATTTTTCAAATTTTTTTTATTCATTATAGAAGGAGTTATTTAATTAATTTTTTA
AGTATTTTTCTTTGTTTAAATTCTATTTTTTTTTATTTGATGAATAACTTTAAATCTTTGT
TATTTGCCCTCATTTTTTGTTTTTGTTGGTTTTTATACTCCTCTTTTATATTTATTTATA
TTTTTTTTTATTATATATTTTTTTTTAAAATTTTTAATTTCAGATTTTACATATAAATTT
TTAGGTGATTATTTACCTAAATTAGGAGTTTTTAATATAAAATCTTTTAAATATTTTGAA
AATTTTTTATATAATTTAAGATCTTTTTTTATTAATTTTTTCACTGGTTTGAGAAATTAT
TTTAATTTTTTTAATTATAGGAGTTTCTTTAATTCAATTGTTTTAATAGTTTTTTTATTT
TTTATTATA------------------------AGAATTTTTCACTTACAATGAAAGGAT
TGAAGCTTTTTATTTTATGTTTTTATTTTAACTTCTTTAGTATTTAGATTTTATTGTTAT
TTAAGTATTGACCCTATAAAGAGTTGTCTTTATTTGGTTTTAAGTTTATTATCTATTTCT
CCACTTTTATCTTTTGGGGTTCAAATTTGGTTTAGATATTTTGTATGTTTAATTTTTTTA

AGTGGAATTTTTGTAATTTTAGTTTATTTTTCTAGTCTTTCAAAATATAATTTTTATAAA
TTTTCTTTTTCTTTATTGATTTTTATAGGTTTGTTTTTTTTACCATTTTTTTTTTATATTT
TTACAAAAAATTATTTATTTAGCCTTTATTTTATA-AATTTTATATATTTATTTATATAT
ATTATTTTTATATTACTTTTTTTTATAAATTTTACTAGTTATTTTTTAAATTTTTCTGGA
GCTTTACGTAAAATT-------------------TTAAAATTATATTTATATTTTTTAGA
TTATTATCTTTAATATTTAAATGACAACGTTTA-TTTTTATTATTATTTCTTTAGAATTT
CTAGTTTTAAGTTTATTTTTAAAATTTAGATATATTTAAAATAGTTATATATTTTTTTTT
TTTATATGTTTTACTGTAATTTCAAGGGTTATAGGTATAGTCTTAATAGTGGGAGGAGTA
AAATTTTTTGGTTCTGATTTAAGTGTTTTTTGC
>Panagrolaimus_superbus_OZ038360
TTATTTATAAAAAAATATTATAATGGTGTTTCTGTTTGATTAGAAAGTTCAAATCATAAA
GATATTGGAACTTTATATTTTATTTTTGGATTGTGATCTGGTATAATCGGTACTAGTTTA
TCAATAATTATTCGTTTTGAGTTAGCTAAACCTGGTTATTTCTTAGGTAATGGTCAACTT
TATAATTCTGTTATTACTGCTCATGCTTTATTAATAATTTTTTTTATAGTTATACCAACT
ATAATTGGTGGGTTTGGTAATTGAATATTACCTCTTATACTAGGTTCTCCTGATATAAGA
TTTCCACGTTTAAATAATTTAAGATTTTGATTATTACCAACTGCAATATTACTTATTTTA
GATGCTTGTTTTGTAGATATAGGAGCAGGAACTAGTTGAACAGTTTATCCTCCTTTAAGA
ACTTTAGGGCATCCTGGAAGAAGTGTAGATTTAGCTATTTTTAGTCTTCATTGTGCAGGT
GTTAGTTCTATTTTAGGAGGAATCAATTTTATGTGTACAACTAAAAATTTACGTAGTAGT
TCTATTTCTTTAGAACATATAAGTTTATTTGTTTGAACAGTTTTTGTAACTGTTTTTTTA
TTAGTTCTTTCTTTACCTGTTTTAGCTGGAGCTATTACTATATTATTAACTGATCGTAAT
ATTAATACTTCTTTTTTTGACCCTAGAGCTGGAGGAAATCCTTTAATTTATCAACATTTA
TTTTGATTTTTTGGACATCCAGAAGTTTATATTTTAATTCTTCCTGCTTTTGGTATTGTT
AGTCAAAGTACTATATATTAAACAGGTAAGAAAGAAGTATTTGGAACTTTGGGTATAATT
TATGCTATTTTAAGTATTGGTTTAATTGGATGTGTAGTTTGAGCACACCATATATATACT
GTTGGTATAGATTTAGATTCACGTGCTTATTTTACTGCAGCAACTATAGTTATTGCAGTT
CCTACTGGAGTAAAAGTTTTTAGTTGATTAGCAACAATATTTGGTATAAAAATAAAATTT
CAACCTTTACTTTTATGGGTTTTAGGTTTTATTTTTTTATTTACTATTGGAGGTTTAACA
GGTGTTATTTTATCTAATTCTAGTTTAGATATTATTTTACATGATACATATTATGTTGTT
AGACATTTTCATTATGTATTAAGATTAGGTGCTGTTTTTGGTATTTTTACAGGTGTTTCT
TTATGGTGAAATTATGTTACTGGGTTAATATATGATAAAACTTTAATATCTGCTGTATTT
ATTTTAATATTTATTGGTGTTAATTTAACTTTTTTTCCTTTACATTTTGCTGGTTTACAC
GGTTTTCCTCGTAAATATATAGATTACCCAGATGCTTATTCAATTTGAAATGTTTTATCT
TCTTATGGTTCAATAATTAGTGTTTTTGCTTTATTTGTATTTTTATATACTTTATTGGAA
TCATTTATATCTTTTCGTGTTTTATTATTTGATTATAGAATTAATAATGGTCCTGAAAGA
AGATTGAGTAATTATATTTTTGGACATAGGTATCAAAGAGAAATTTATTTTAGTGTAAGA
CATTTAAATAACTATTTTCAAGGTTATAATTTAAATTTTTCTTATAGTCTTTTTTCTAGA
TATATAGATTGATTTCATAGTTTTAATTCTAGATTAATAATCGGTGTATTGTTGTTTGTT
TCTTCTATATTTATTTATTTAATTTTTTCTAGTTTTTATTTTAAAAGAAAAAAAATTGAA
TATCAGTTTGGAGAACTTTTATGTAGAATTTTTCCAACAATTATTTTATTAGTACAAATA
GTTCCTTCTTTAAGTTTATTATATTATTATGGATTAATAAATATTGATAGTCAAATTTCT
GTAAAAGTAACAGGTCATCAATGATATTGAACTTATGAAGTAGGAGATATTCCTGGTTTA
GAATTTGATTCTTATATAAAATCTTTAGATTTATTAAATTATGGTGAACCTCGTTTATTA
GAAGTTGATAATCGTTGTATTATTCCAATAGATACTAATATTCGTTTTTGTATTTCTTCT
GCAGATGTTATCCATGCTTGAGCTCTTTCAAATTTATCTATTAAATTAGACGCTATAAGA
GGTATTTTAAGTATTTTGAGATATAATTTTCCTGTAGTTGGAGTTTTTTATGGACAATGT
TCGGAGATTTGTGGTGCAAATCATAGATTTATGCCTATTGCTCTTGAAGTAACATTGTTT
GATTTTTTTAAAAGTTGATGTTTATTAATATTA------------------CATAATTTT
CATATTTTAAGTTTATCAACTTTTCCTTATTTAGTTTTTATAGTTACTTTTTCTCTTACT
ACTTCAATTGTAATATTTTTTAAATTTGGTAATTTTTATTTTTTACTTTTAAGTTTAATT
ATTTTAATTTATATCTCTTTTTGTTGATCTAAAGATATTGTAATAGAAGGATTAAGAGGT
TATCATAATTTTTTTGTTATAGATGGTTTTAAATTTGGAGTTGTTTTATTTATTTTTAGT
GAATTTATATTTTTTTTTAGTATTTTTTGAACTTTTTTTGATTCAGCTCTTGTACCTGCT
CATGATTTAGGAGAAATATGGTCTCCTATTGGTTTAGAATTAGTTAATCCTTTTGGAGTA
CCTTTATTAAATACTATTATTTTATTAAGAAGAGGTGTAACTGTAACTTGAGCTCATTAT
AGTTTATTAAGTAATAAATCTTGTTCTTTAAGTATAATTTTAACTGTGTTTTTAGCTATT
TTATTTACTGGTATTCAAGGAATAGAATATAATGAGGCTAGTTTTTCTATATCTGATGGA
ATTTTTGGTAGATGTTTTTATCTTGCTACAGGTTTTCATGGTCTTCATGTACTTTTTGGG
GGTTTATTTTTATTTTTTAATATATTACGTTTATGAATATCTCATTTTAACTATAATCAT
CATTTAGGCTTAGAATTTGCTATTATTTATTGACATTTTGTGGATGTTGTTTGATTATTT
TTATTTGTTTTTGTTTATTGATGA------------------------------------

------------------------------------TTATTAACTTGTTGTTTTGGAGGT
TATTTTTGTTATTCTTTTTGTCCTTGTGGAATACCTGAATTTACTTTTATTTTTGCTTTA
ATAGCTTGAATAAGTGTTTTTATTACTTTTATTTCTTCAGAAAAATTTTCTATTTATATA
AGTAAATCAGGTGATAAATTTTTAAAAACTTTAAGAATATTAGCTGTAGAATTAGTAAGA
GAGTTTTCACGTCCAGTAGCTTTAACTGTTCGTTTAACAGTTAATATTATAGTAGGACAT
TTAATTAGAATAATATTATATCAACTTTTAGAACTAAGTTTAGGTAATTTTTATTTATGA
ATTATTGTATTAGCTATTTTAATAGAATGTTTTGTATTTTTTATTCAAAGTTATATTTTT
TCTCGTTTAATTTTTTTATATTTAAGTGAA---TTAAAACAATTAATTTATTTTGTTAAT

TCTTTATTGATTTCTTTACCAAGAAGAAAAGCTTTAAGTTTAAATTGAAATTATGGTAGA
ATACTGGGTATAGTTTTAATTTTTCAAATTATTACTGGTACTTTATTAGCAATTTATTAT
TCTGCAGATAGTTTATTGGCTTTTAATAGAGTTCAATATATTATGTATGAAGTTAATTAT
GGTTGAATTTTTCGTATTTTTCATTTTAATGGGGCTAGTTTATTTTTATTTTTTTTGTAT
TTACACTTTTTTAAAGCTTTATTTTTTTATAGTTATCGTTTAAAATTTGTTTGAGCTTCA
GGTTTAACTATTTTTTTATTTGTTATAATAGAAGCTTTTATAGGTTATGTTTTAGTTTGA
GCTCAAATAAGTTTTTGAGCTTCAGTAGTAATTACTAGTTTATTAAGAGTTATTCCAATT
TGAGGGCCTATTTTAGTTGTTTGAATTTGAAGAGGTTTTACTGTTTCTGGTGCTACACTT
AAATTTTTTTTTGTTTTACATTTTTTAGTACCTTATTTAATTTTAGTTGTAATATTAGTA
CATTTAATTTTTTTACATACTACAGGTAGTACTTCGAAATTATTTTGTCATGGAGATTAC
GATAAAATTAGATTTTTTCCTTATTATTGATTAAAAGATTCTTATAATTTAATTTTTTGA
TTGTTATTTTTTTCTTTTTCTCTTCTTTATCCTTTTAATTTAGGAGATCCAGAAATATTT
ATTGAAGCTGATCCAATAATAAGTCCTGTACATATTGTTCCTGAATGATATTTTTTATTT
GCATATGCTATTCTACGTGCTATTCCTAATAAAATTTTAGGAGTTATAGCTCTTTTAATA
AGAATTGTAAGATTTTATTTTTTTATTTTAGTTGAAAGTTATCATTCTGTTTTAAAAACT
TTAAATAAATTTTTAGTGTATTTTTTTATTTTTATTTCTATTATTTTAAGTTGATTAGGA
CAATGTTTAGTTGAAGATCCTTTTGTATTTTTAAGAGCTATTTTTTCAATTCTTTATTTT
TTCATAGTA---
--TTAATT
GTTTTTGTTTTACAGGCAATTGCTTTTATTACTTTATATGAACGTCATTTATTGGGTTTG
TCTCAAAATCGTATAGGACCAACTGTAGTAAGTTTTAAAGGTATTTTACAAGCTTTTATA
GATGGTTTAAAATTAATTAAAAAAGAACAAATTTTACCAATAAATTCTTCTATATTTTTA
TTTTTATTTGTTCCTGGTATATCTTTTGTTATAATATTAGTTGATTGAATGGTTTTACCT
TATATATTTAATTTTATTACTTTTCAATATTCTTTATTATTTTTTTTATGTTTAGTTGGA
TTTAGAGTATATACTACTATAATTAGTGGTGTAGTTAGAAAATCTAAATATCCTATATTA
GGTTCTGTTCGTTCAAGAAGACAAAGAATTTCTTTTGAAATTGCTTTTTCAATTTATATA
TTTTGTATTATTTTTCATTTAAATCAGTATTCTTTTTTAAATTTTTTTAATTTAAATTTA
ATTTTTTTATTGATTCCATTTTTATTAATAATATTAGCTGAACTAAATCGTGCACCTTTT
GATTTTTCAGAAGGTGAGAGAGAATTAGTAAGAGGTTTTAATGTAGAGTATTCAAGAGTT
GGATTTGTTTTTTTATTTTTAAGAGAGTATGGAATATTGATTATATTTAGAGTTTTGTTT
TCTGTTTTATTTTTAAATTTTTCTTTAAGGGGTTATTTTTTAATATTTAGATTATTAATT
TTTATTCGTAGTTCTTATCCTCGTTATCGTTATGATATATTAATGAGATTTTTTTGATTT
AAACTGTTACCTCTTTCTATTATTTTTTTATTTTATTATTTT------------------

---AATTTATTAATTTGGTGAAGAGTTTTTGTTATAATAACAATAACTTTTATTTTATTA
AATAAAATTCAAGGAGATTTTGGAAATAGATTGAATTATTTTTTATTACAAGAATTTTTA
GGTTTTTTATTTCTTATTTTTTTTTCTACTATATTTCAATATATTTTATTAATAATAAAA
GTAGGAATGAGTCCTTTTCATTTTTGAGTAACATCTGTTTTTTTAAATTTAGAAAATTTT
ATATTATTATGGTTTTTAACTTTTCAAAAACTTCCTTTTATTCCTATTTTAATGTTATTA
TTTAAATTTATTTTTTTTTTATTTAATTTTATTTGGAGTGGTATTTTGTTATTTTCAATTG
TTAAATATAAAAAACTATAAAATTATATTTATATTGTCTTCTACTGAATCTTTTAATTGA
CTTTTAATAGGTTTATTAATAGGAATTTGAGGGATAATATTAATATCTTTTTATTATATA
TTAAATATATTATTTTTAATAAATTATCAAATTTTATTTAATAATATAAATTATTCTTTA
GAACTTTTTTTAGTTTTTCTTAATATTCCTTTATCTTTTACTTTTTTTTTAAAAATTTTT
ATATTAAAT---

------------------------------TTGTTTTTTTCATTTATTCTTGTTTTTATT
TTTTATTTATTAAATTTTTTTTTAAGAGAAAAAAATTTTTATTTAATTAAAAATTCTTCT
TTTGAAAGAGGTTTTGAAAGAATTGGAAAAATTCATAATTCTTTTAGTATTCATTTTTTT
ATTATTATATTAATATTTGTAATTTTTGATTTAGAAGTTGTAATGCTTTTAGGTTTTTTA
ATTTCTTCATTTTATTCAATTTATACTTTTTTTTTAATTTTTTTTATTTATTTTAGGAGGG
TTTTATATAGAGTGATTTTTTGGAAAATTACTTTGA------------------------

---GTTATATTT
TTTTTTTCTTCTAATATTTTAAGTTTATTTATTTTTTTTTGAATTATCTATATTTCCTATT
ATTATAATAATTTTAGGTTATGGTTCTCAAATTGAAAAAATTAATTCTAGATATTATTTA
ATATTTTATGCTTCTTTTTGTTCTTTTCCTTTTTTATTTATTTATTTTTATTTTGATTTA
AGATTTCATTATGTTTATTTTTTTAAAATTTTTAAGTTGAGAGTTGGTTTTTTTTTTTAACT
TTAGGATTTATAATAAAATTTCCTGTATATTTTTTACATTTATGATTACCTAAGGCTCAT
GTTGAAGCTCCTACTTCAGCAAGTATATTATTGGCAGGTTTACTTTTAAAACTTGGAACT
GTAGGTTTTTTTCGTATTATAAAAGTTTTAACTTTTATTCATTTAAATTTTTGATTTTTT
TTAGCTTTTTTGGGTATAATTTTTTCTTCTTTTAATTGTGCTTTTCAAAGAGATTCTAAG
TCTTTAGCAGCTTATTCTTCTGTAACTCATATGGGTTTTACTTTATTATGTTTAATTTTA
TTAAGAATTGAAAGTAAAATTTCTAGTTTAATAATAATATTAGCACATGGTTTTACTTCA
ACTTTAATATTTTTTTTTATTGGAGAATTTTATCATAAAAGTAGAAGACGTATAATATAT
TATTTTAATAGTTTTATAAATATTTCTATTATATTAACTATTTTATTTACTTTAACAATA
ATTAGTAATGCTGGAGTACCCCCCTCTTTAAGTTTTTTATCTGAATTTTTAACTATTTTA
ATTTTATTAAATTTTAATAAAATATTTTTTATTGTTTTAATAGTATATTTTTTTTTTGCA

```
TTT---------------------------------------------------------
------------------------------------------------------------
------------------------------------------------------------
------------------------------------------------------------
------------------------------------------------------------
---------------------------------------------TTAAAATATAATTTT
TATTTAAATTCCTTGGTTTTTTCTTTAATTTTATTAATTGTTTCTTTAAGTGTTTTAATT
TTTAGTTCTTATTATTTATCTGGAGAATTAAATTTTTATTATTATTATTTTTTATTAATA
ATATTTGTTTTAAGAATGTTTAGTTTAAATTTTAGTTTAAATAGTTTTAGAATATTATTA
AGTTGAGATCTTCTTGGTATTACAAGATTTTTTTTAGTTCTTTTTTTATAATAATTGAGAT
AGAAATAGAGGTTCAATAAATACAGTTTTAACTAATCGTTTAGGTGATTTTTTTTTTATTT
GTATTTTTTTTCTTTTTCTTATTTTCTTAATATAAATTTTTATTCTTATATGTTTATATTG
TGAGGGCCGTTATTGTTTTTAATTTTTACTGCTTTTACAAAAAGTGCTCAATTTCCATTT
AGAGGTTGATTACCTAAAGCTATAAGTGCTCCTACTCCTGTAAGATCTTTAGTACATAGA
AGAACTTTAGTAACTGCAGGTTTATTATTACTTTTTAATTTTTCATTAATATTAATAAAT
ATAAAAGTTATAATAGTTATTTTTTTTTATTGGTTTATTGACTACTTTTTTTTTCTAGTTTA
ACTGCTGTTGTTGAAGAAGATATAAAAAAAGTTGTAGCTCTGAGTACTTTATCTCAAATA
GGTTTTTCTATAATTACTTTAGGTTTAGGCTATTATTTAGTAGCTCTTATTCATTTATTA
AGTCATGCTTTATTTAAAAGATGTTTATTTATACAAATCGGTTATTTAATTCATTGTTCA
TTTGGTCAACAAGATGGACGTTTTTATAATAAATTAAATTTTATTCCTTTTTTTTATTCAA
ATTCAATTATTATTAACTTTATTTTGTTTATGTGGTTTATTCTTTTATAGGGGTCTTGTA
AGTAAAGATTTAATTTTAGAGTATTTTTATTCTAATAATTGATACTTTCCTGTAATATTT
TTATTTTTTTTTACAATTTATTTAACTTTTTTTTATAGTTATCGTTTA------------
------------------------------------------------------------
------------------------------------------------------------
------------------------------------------------------------
------------------------------------------------------------
------------------------------------------------------------
------------------------------------------------------------
------------------------------------------------------------
------------------------------------------------------------
---------------------TTTTTATTTCTTTCTTTATTAATATCTTTTTTTTGTTAT
CTTTCTATTGATCCTATAAAAAGTTGTTTATATCTTGTATTAAGACTTATTTTATTAACT
CCTTTTTTATCTATAGTTAATGAGACTTGATATAGTTATTTTATTTGTATAATTTTTTTA
AGTGGTATTTTTGTTATTCTTGTTTATTTTACAAGAATATCAAAATATATTTTTCATAGG
TTAAGTTTAAGTTTTTTTATTTTTTTAGGATTATTTTTTTTACCTATTTTGTTTTTAAAT
TATAATTTTTCATTAAATTCTATTTATTATTTAGAATATTTATATTTTTTTTTTATTTTTA
ATTTTAAATTTAACTTATTTTATGAATTTTTCTAGTTATTTTTTAAATTACTCAGGTGCT
TTACGTAAAGCT---------------------------ATAATATATTTATTTTTTAGA
TTAATCTCTATAATATTTAAATGACAACGTTTAATTTTTATTTTAATTTCTTTAGAGTTT
TTAGTAATAAATCTTTTTTTATTTTTTTTCTTTTTATTTATCTTTTATTATATATTTTTTT
TTTATATCTTTTACTGTTATTTCAAGAGTTTTAGGAATAATTATAATAGTTGGAAGTCTA
AAATTTTATGGAAAAGATAAAAGATTGTTT---
```
