# Supplementary figures and images for: Wood-Inhabiting Nematode, Bursaphelenchus ussuriensis sp. n. (Nematoda: Aphelenchoididae) from David Elm, with Molecular Phylogeny of the Genus Based on Partial Mitochondrial Genomes
Source: Plants (Basel). 2024 Dec 31;14(1):93. doi: 10.3390/plants14010093 (PMC11722724; doi:10.3390/plants14010093)

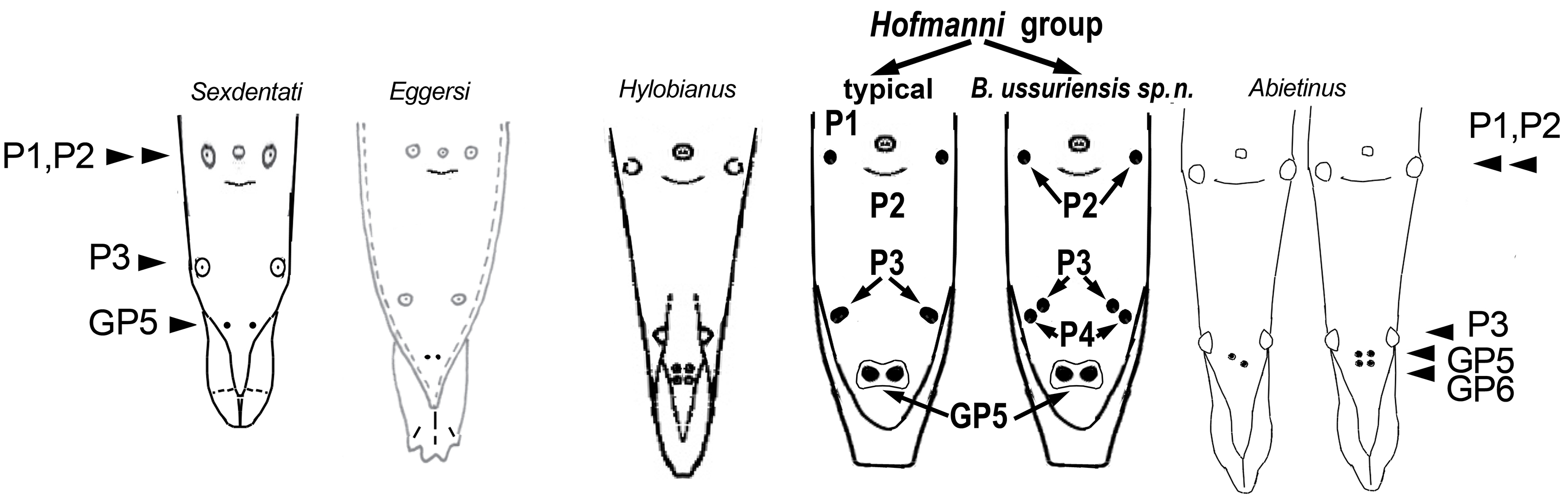

Supplement: Supplementary file 1 [file plants-14-00093-s001.zip › Fig S1. Supplement_ Male papillae.tiff]
